# Supplementary material for: Balancing elementary steps enables coke-free dry reforming of methane
Source: Nat Commun. 2023 Nov 18;14:7514. doi: 10.1038/s41467-023-43277-0 (PMC10657353; doi:10.1038/s41467-023-43277-0)
Supplement: Supplementary file 1 — Supplementary Information [file 41467_2023_43277_MOESM1_ESM.pdf]

Supplementary Information

Balancing elementary steps enables coke-free dry reforming of methane

Jiaqi Yu[1,§], Tien Le[2], Dapeng Jing[3], Eli Stavitski[4], Nicholas Hunter[5], Kanika Lalit[1], Denis Leshchev[4], Daniel E. Resasco[2], Edward H. Sargent[6,7,*], Bin Wang[2,*], Wenyu Huang[1,*]

[1]Department of Chemistry, Iowa State University, Ames, Iowa 50011, United States

[2]School of Sustainable Chemical, Biological and Materials Engineering, University of Oklahoma, Norman, Oklahoma 73019, United States

[3]Materials Analysis and Research Laboratory, Iowa State University, Ames, Iowa 50010, United States

[4]National Synchrotron Light Source II, Brookhaven National Laboratory, Upton, New York 11973, United States

[5]Department of Mechanical Engineering, Iowa State University, Ames, Iowa 50011, United States

[6]Department of Chemistry, Northwestern University, Evanston, Illinois 60208, United States

[7]Department of Electrical and Computer Engineering, Northwestern University, Evanston, Illinois 60208, United States

[§]Present address: Department of Chemistry, Northwestern University, Evanston, Illinois 60208, United States

[*]Correspondence to: whuang@iastate.edu, wang_cbme@ou.edu, ted.sargent@northwestern.edu

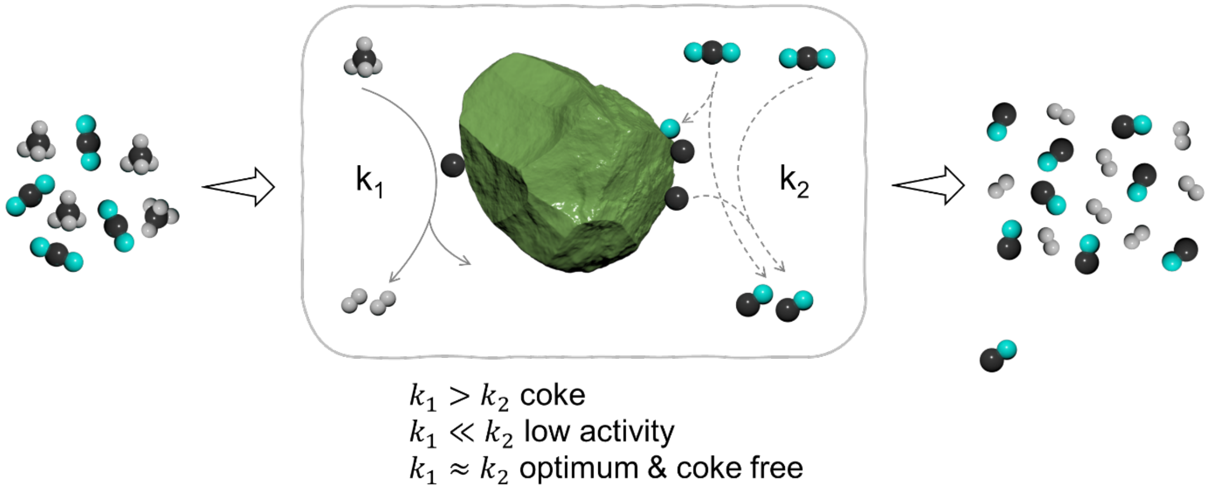

Supplementary Fig. 1 | Schematic illustration of the design concept of coke-free dry reforming of methane catalyst through balancing kinetics for thermodynamic optimization. The optimized catalyst has the balanced kinetics between CH_4 dehydrogenation rate (k_1) and coke removal rate (k_2). We considered both CO_2 activation followed by C* removal with O* route and reverse Boudouard reaction route as CO_2-assisted coke elimination mechanism. Black ball: carbon; grey ball: hydrogen; blue ball: oxygen; and green stone: $CoAl_xGa_{(1-x)}O_4$-R.

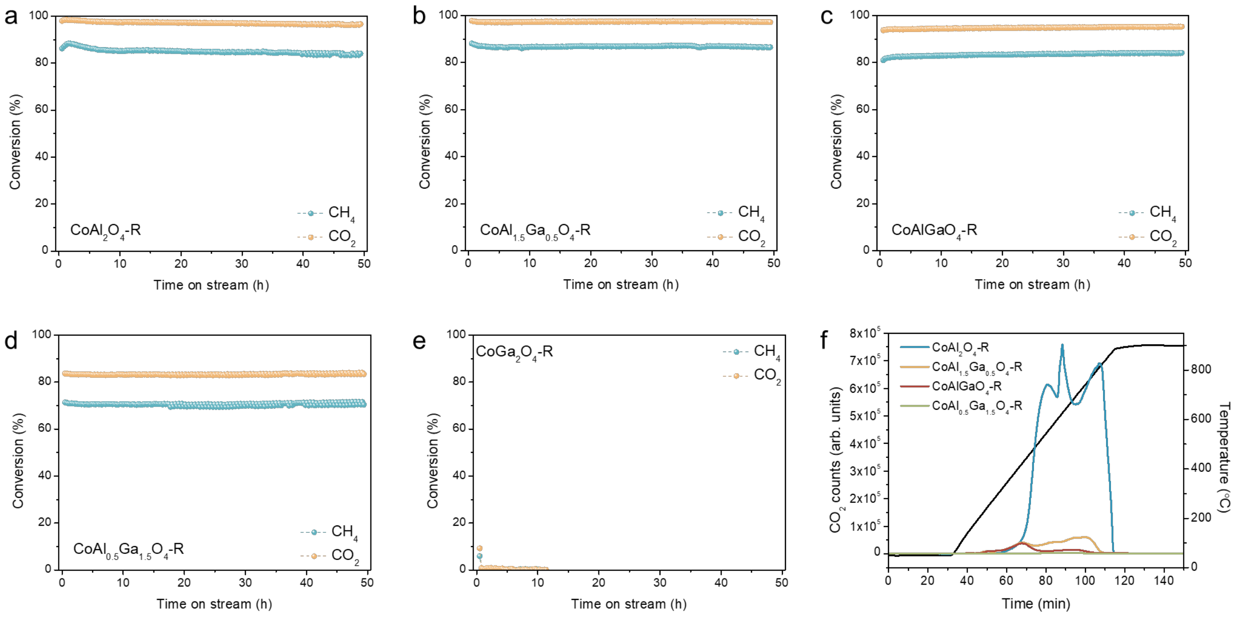

Supplementary Fig. 2 | DRM performance of $CoAl_xGa_{(2-x)}O_4$-R (x = 2, 1.5, 1, 0.5, and 0) catalysts. (a-e) 50 h test at 800 °C with 300 L g_{cat}^{-1} h^{-1} reaction gas feeding (CH_4:CO_2:He=1:1:8); and (f) O_2-TPO after 50 h reaction. At the elevated temperature, such as 800 °C, the conversion of Co-spinel derived catalysts reaches equilibrium conversion. Equilibrium conversions of CH_4 and CO_2 at 800 °C are 95.0% and 97.3%, respectively(*1, 2*).

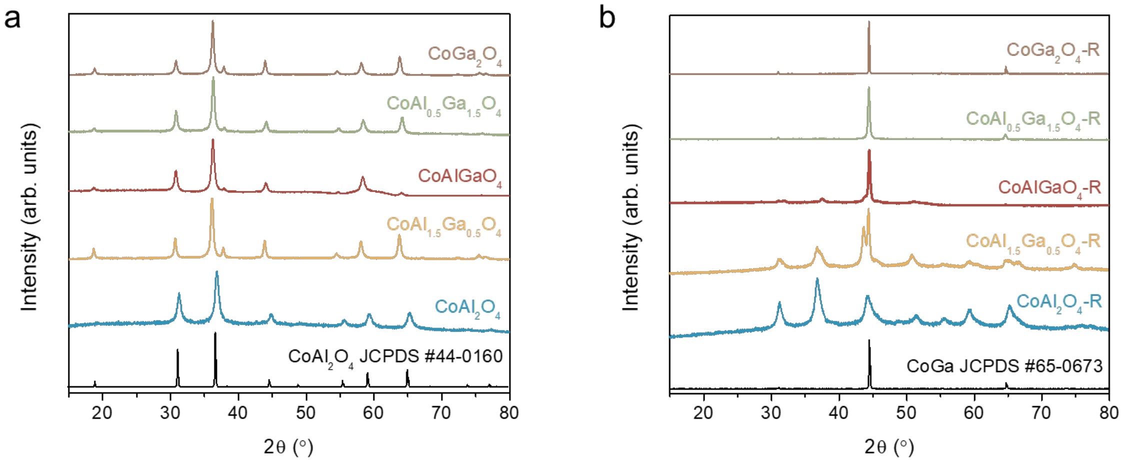

Supplementary Fig. 3 | PXRD pattern of $CoAl_xGa_{(2-x)}O_4$ spinel oxides (a) before and (b) after reduction. Black lines: standard diffraction patterns of $CoAl_2O_4$ (JCPDS #44-0160) and CoGa intermetallic compound (JCPDS #65-0673).

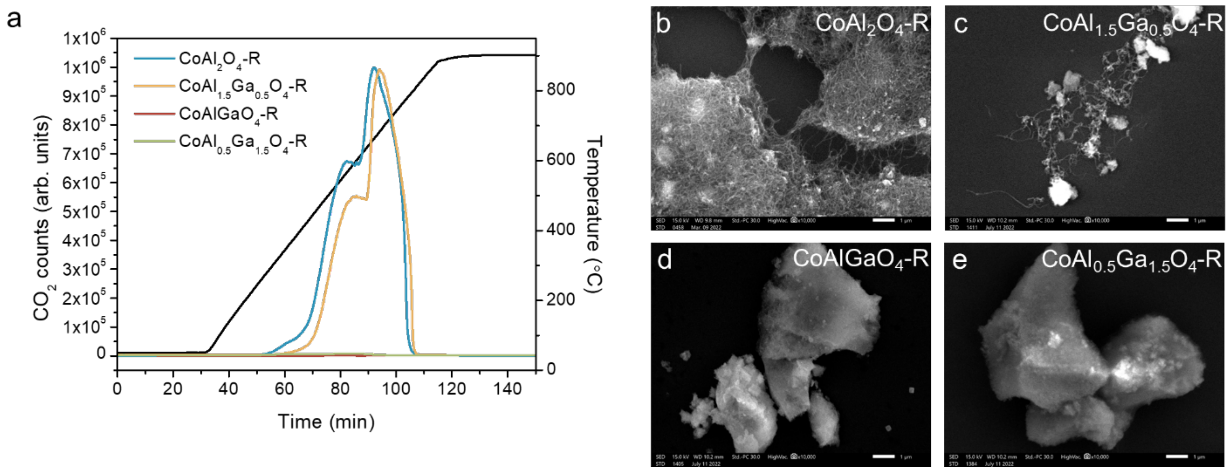

Supplementary Fig. 4 | Characterization of $CoAl_xGa_{(2-x)}O_4$-R catalysts after 100 h stability test at 700 °C. (a) Post-reaction O_2-TPO with CO_2 mass signal (m/z=44) recorded. (b-e) SEM images of spent catalysts, showing the presence of carbon nanotubes in spent $CoAl_2O_4$-R and $CoAl_{1.5}Ga_{0.5}O_4$-R catalysts. Scale bar: 1 μm.

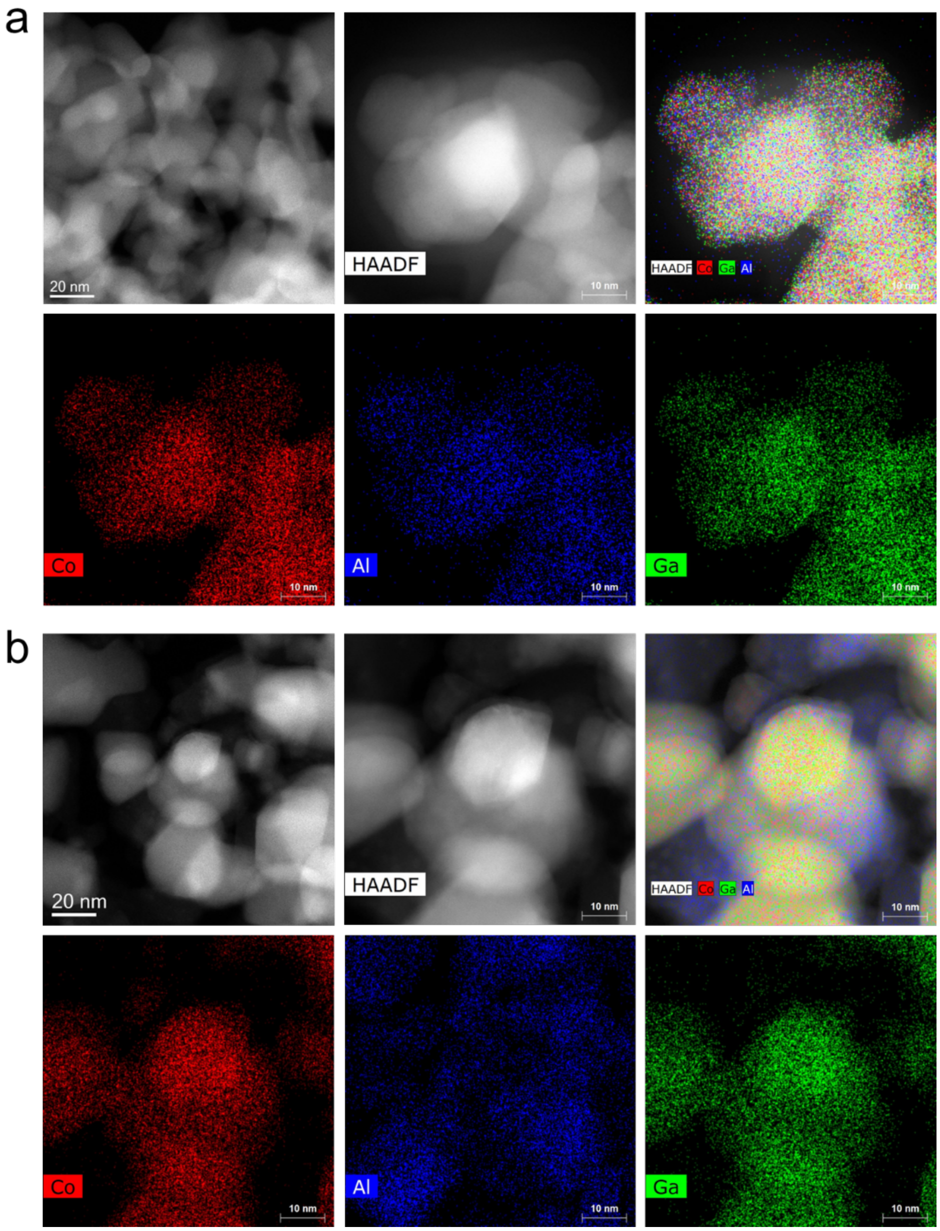

Supplementary Fig. 5 | HAADF-STEM and STEM-EDS elemental mapping images. $CoAl_{0.5}Ga_{1.5}O_4$ spinel oxides (a) before and (b) after reduction. After reduction, the distribution of Co and Ga largely overlaps, indicating the formation of CoGa intermetallic structure (agreeing with XRD characterization), and Al surrounds the CoGa intermetallic particles.

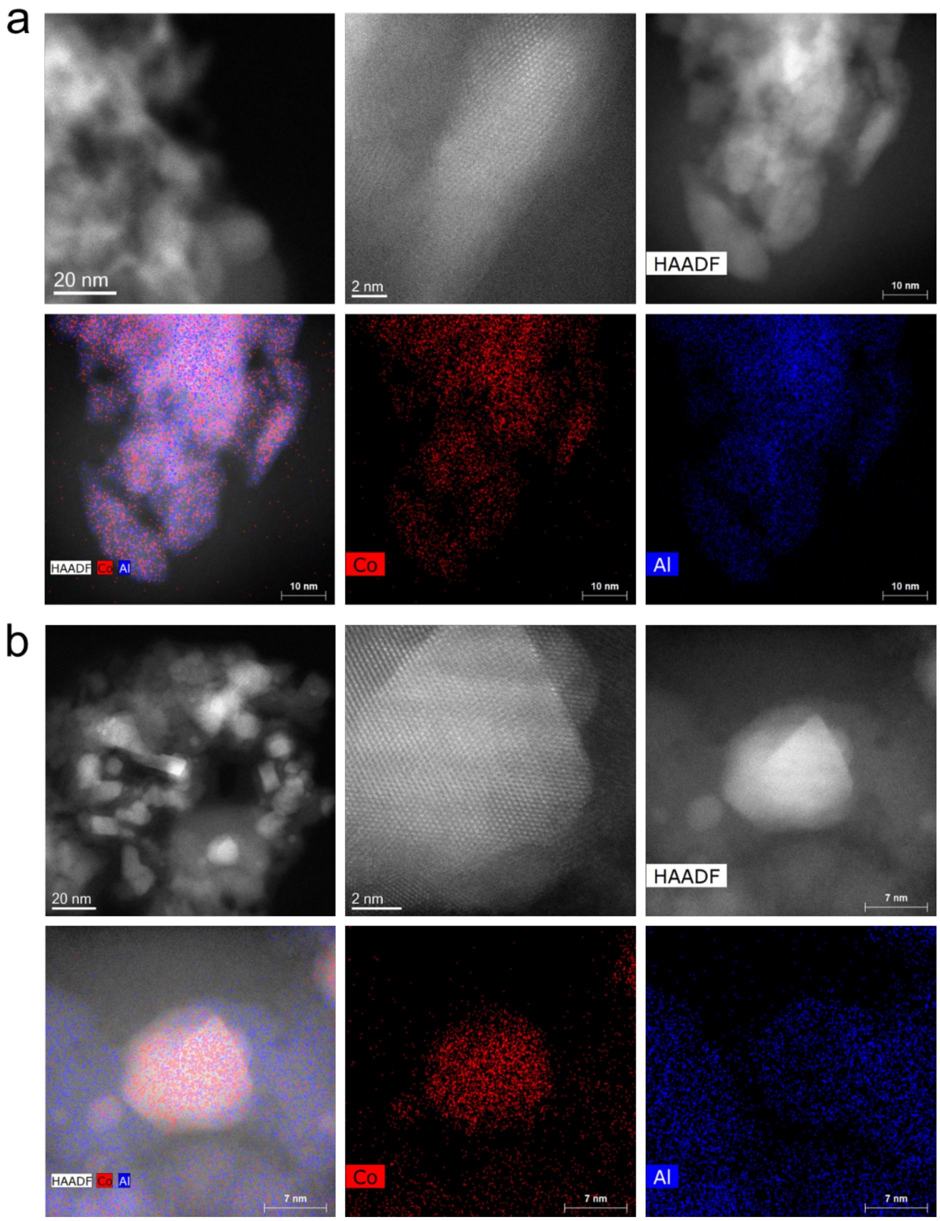

Supplementary Fig. 6 | HAADF-STEM and STEM-EDS elemental mapping images. $CoAl_2O_4$ spinel oxides (a) before and (b) after reduction. Before reduction, Co and Al uniformly distribute in $CoAl_2O_4$ spinel oxides, while Co nanoparticles formed after reduction.

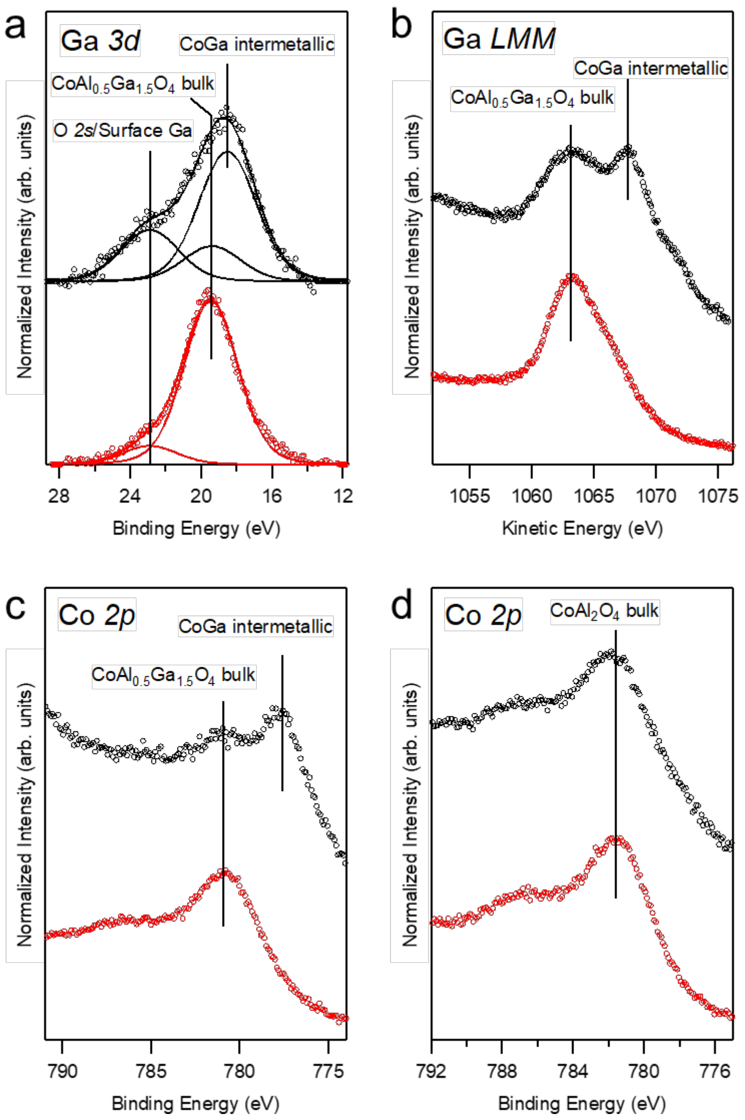

Supplementary Fig. 7 | XPS spectra of $CoAl_{1.5}Ga_{0.5}O_4$ (a-c) and $CoAl_2O_4$ (d). Ga *3d* (a), Ga *LMM* (b) and Co *2p*$_{3/2}$ (c) $CoAl_{0.5}Ga_{1.5}O_4$ spinel oxides before (red) and after (black) reduction. Before reduction, the Ga *3d* spectrum showed a strong component at 19.6 eV attributed to the Ga from the bulk of $CoAl_{0.5}Ga_{1.5}O_4$ and a weak component at 22.9 eV attributed to a mixture of O *2s* and surface oxidation of Ga. After reduction, a new component at 18.6 eV, 0.2 eV higher than metallic Ga, emerged and was assigned to intermetallic CoGa. The Ga *LMM* Auger region showed a similar new component emergence at 1067.8 eV after reduction, as well as a new Co component emerged at a lower binding energy of 777.3 eV. This is 0.8 eV lower than metallic Co. We assigned this component to intermetallic CoGa.

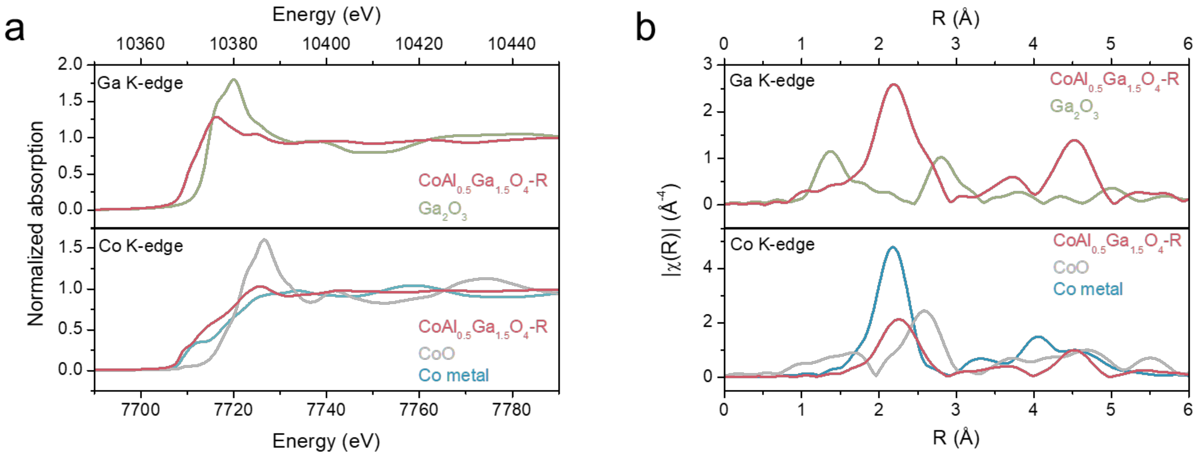

Supplementary Fig. 8 | XAS study on $CoAl_{1.5}Ga_{0.5}O_4$. (a) XANES and (b) EXAFS of $CoAl_{1.5}Ga_{0.5}O_4$-R on both Co and Ga K-edge. Co K-edge XANES shows that the edge position in the $CoAl_{1.5}Ga_{0.5}O_4$-R is shifted towards lower energy compared to the metallic Co, which supports alloying with lower electronegative Ga (1.88 for Co vs. 1.81 for Ga) and hence higher electron density on Co. Fourier transformed EXAFS (FT-EXAFS) of Co shows strong peak at 2.25 Å (non-phase corrected value) which is higher than the Co metal (2.2 Å) which is consistent with lattice expansion due to alloying. CoO FT-EXAFS is shown for comparison, indicating no detectable Co-O coordination. In the case of Ga K-edge XANES the $CoAl_{1.5}Ga_{0.5}O_4$-R spectrum is shifted to the low energy compared to Ga_2O_3 by 5 eV, which together with the white line intensity reduction is consistent with Ga in primarily metallic state. Inspection of Ga FT-EXAFS of $CoAl_{1.5}Ga_{0.5}O_4$-R demonstrates that the first strong contribution is at 2.2 Å which can be assigned to Ga-metal coordination, while small contribution of Ga-O coordination cannot be ruled out.

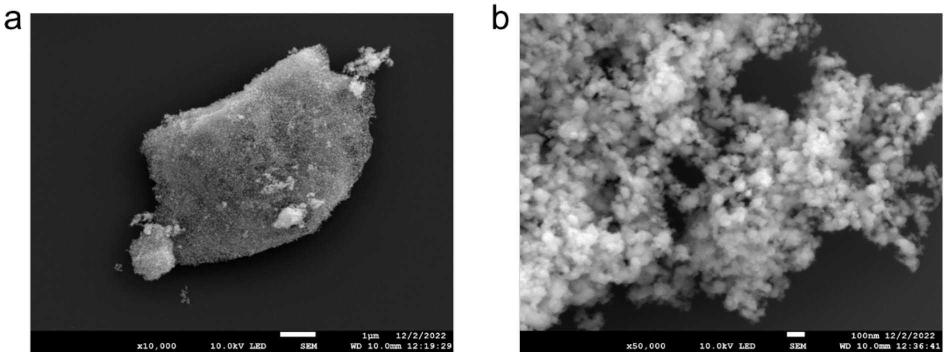

Supplementary Fig. 9 | Post-reaction (700 °C, 1000 h) characterization. SEM images of spent $CoAl_{0.5}Ga_{1.5}O_4$-R. Scale bar: 1 μm (a), and 100 nm (b).

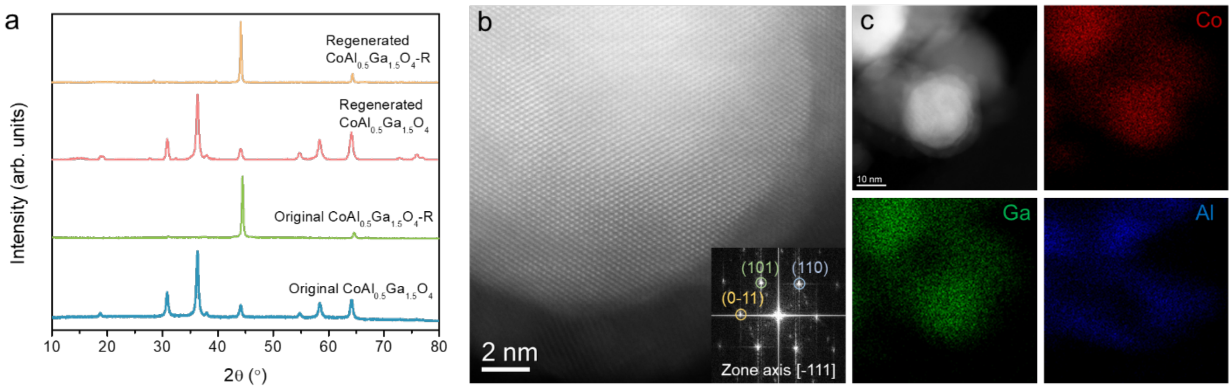

Supplementary Fig. 10 | Regeneration characterizations. (a) PXRD pattern of regenerated $CoAl_{0.5}Ga_{1.5}O_4$ (pink) and regenerated $CoAl_{0.5}Ga_{1.5}O_4$-R (yellow), compared with original $CoAl_{0.5}Ga_{1.5}O_4$-R(green), and $CoAl_{0.5}Ga_{1.5}O_4$ (blue). The XRD pattern of regenerated $CoAl_{0.5}Ga_{1.5}O_4$ and regenerated $CoAl_{0.5}Ga_{1.5}O_4$-R match with original $CoAl_{0.5}Ga_{1.5}O_4$ and $CoAl_{0.5}Ga_{1.5}O_4$-R, respectively. (b) HR-STEM image of regenerated $CoAl_{0.5}Ga_{1.5}O_4$-R; inset FFT of (b), showing CoGa intermetallic structure. (c) STEM-EDS mapping of regenerated $CoAl_{0.5}Ga_{1.5}O_4$-R. Analysis of the images reveals that the designed $CoAl_{0.5}Ga_{1.5}O_4$-R is regenerable with simple calcination and reduction.

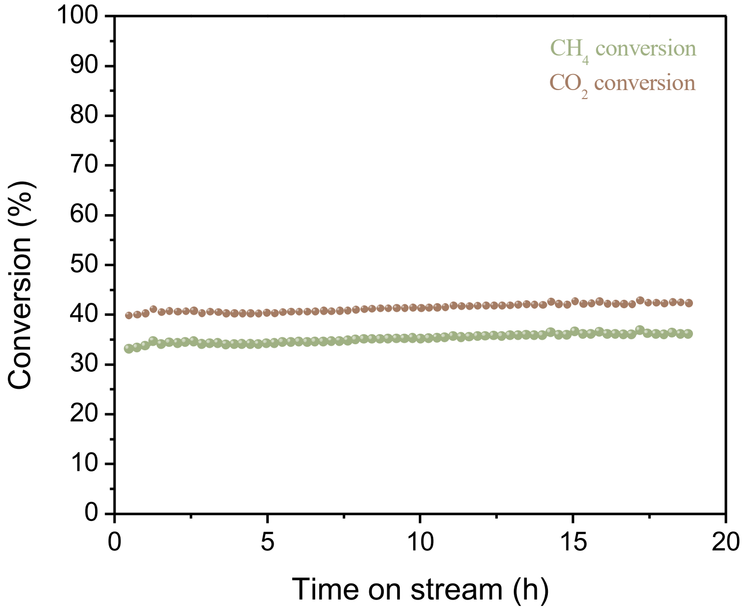

Supplementary Fig. 11 | DRM evaluation of $CoAl_{0.5}Ga_{1.5}O_4$-R with non-diluted CH_4 and CO_2 gas mixtures. Reaction conditions: 700 °C, CH_4/CO_2=50/50 SCCM, GHSV = 300 L g_{cat}^{-1} h^{-1}.

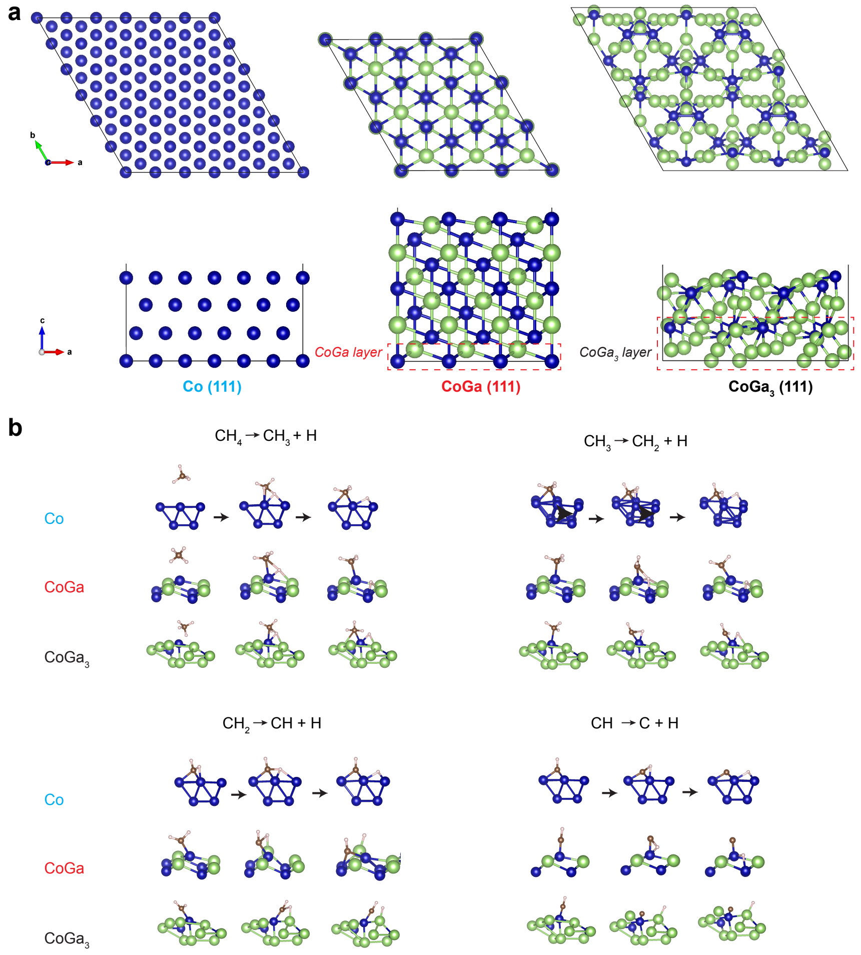

Supplementary Fig. 12 | Catalyst model and configuration of dehydrogenation steps. (a) The model of catalyst surface: Co(111), CoGa(111) and $CoGa_3$(111) (b) Configuration of initial state, transition state and final state of CH_x dehydrogenation on Co, CoGa and $CoGa_3$.

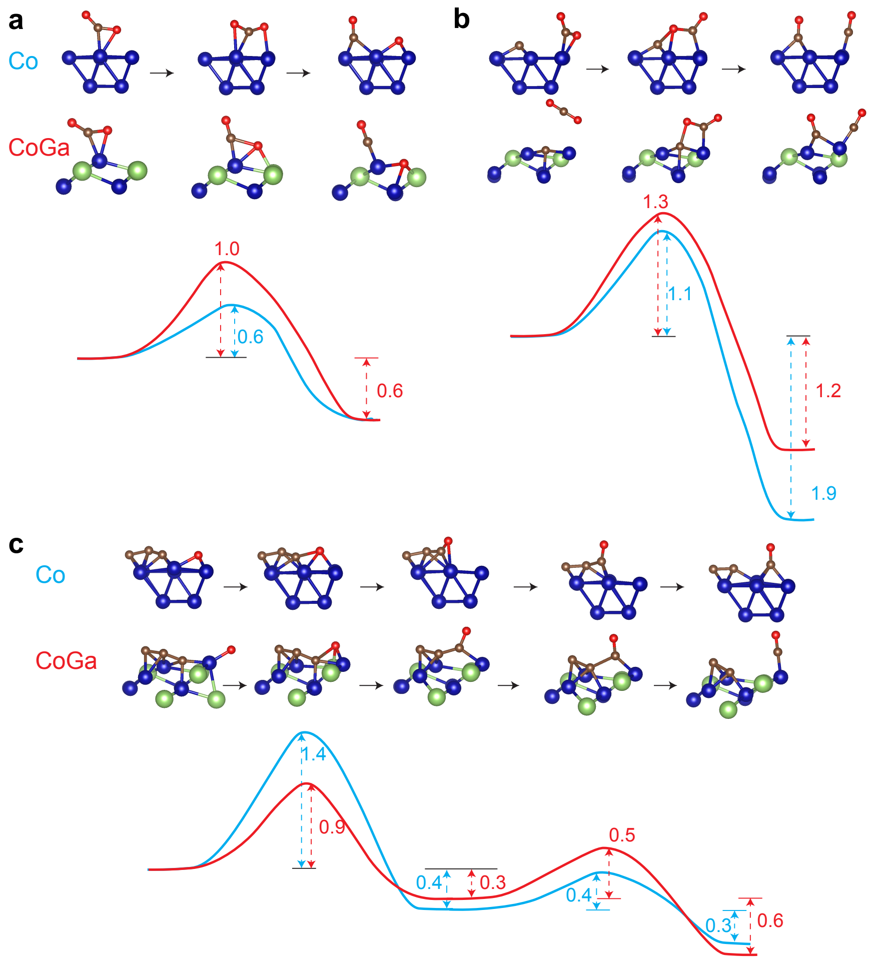

Supplementary Fig. 13 | Additional DFT calculations of DRM reactions. (a) CO_2 dissociation to CO and O, (b) Reverse Boudouard reaction and (c) Carbon *direct* oxidation on Co (blue) and CoGa (red).

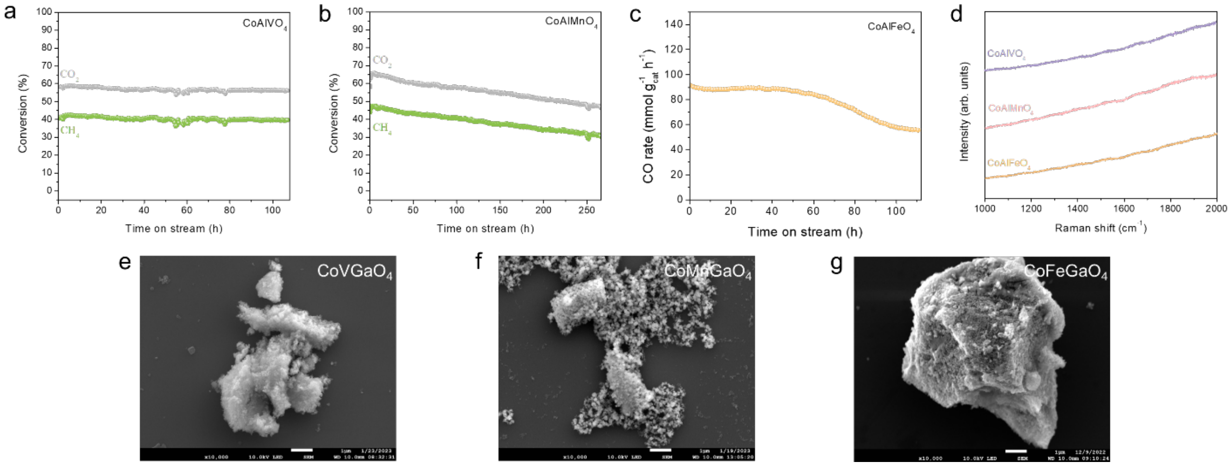

Supplementary Fig. 14 | Demonstration of a series of coke-free DRM catalysts, $CoAlMO_4$-R. DRM performance in (a) 100 h stability test of $CoAlVO_4$-R catalyst; (b) 250 h stability test of $CoAlMnO_4$-R catalyst; and (c) 110 h stability of $CoAlFeO_4$-R catalyst. DRM was performed at 700 °C with 300 L g_{cat}^{-1} h^{-1} reaction gas feeding (CH_4:CO_2:He=1:1:8). (d) Post-reaction Raman spectra. SEM images of (e) spent $CoAlVO_4$-R, (f) spent $CoAlMnO_4$-R, and (g) spent $CoAlFeO_4$-R; scale bar: 1 µm. As predicted, $CoAlMO_4$-R presented coke-free DRM over a long-term stability test. Like $CoAlGaO_4$-R, $CoAlVO_4$-R performed stable activity over a 100 h DRM test. Interestingly, even though $CoAlMnO_4$-R and $CoAlFeO_4$-R showed less stable CH_4 and CO_2 conversion, unlike other unstable catalysts, they remained coke-free, which indicates the concept of balancing kinetics is generally applicable in preventing coke formation. Tuning Al to M ratio and reaction conditions could improve the C-H activation rate and prevent coke deposition simultaneously.

Supplementary Table 1 | Reaction energy and activation energy on Co (111), CoGa (111) and $CoGa_3$ (111). Unit: eV

Reactions	Co (111)		CoGa (111)		$CoGa_3$ (111)	
	E_a	ΔE	E_a	ΔE	E_a	ΔE
**CH_4 activation**						
• $CH_4 \rightarrow CH_3$ +H	1.0	0.2	0.8	0.3	0.6	0.3
• $CH_3 \rightarrow CH_2$+H	0.7	0.4	1.0	0.5	0.7	0.7
• $CH_2 \rightarrow$ CH +H	0.3	-0.2	0.7	-0.6	2.1	2.0
• CH $\rightarrow$ C +H	1.1	0.5	1.2	0.7	2.0	1.9
**CO_2 activation**						
• $CO_2 \rightarrow$ CO + O	0.6	-0.6	1.0	-0.6	-	-
• C + $CO_2 \rightarrow$ 2CO	1.1	-1.9	1.3	-1.2	-	-
**Coke removal**						
• O + C-C-C $\rightarrow$ C-C-C-O	1.4	-0.4	0.9	-0.3	-	-
• C-C-C-O $\rightarrow$ C-C + CO	0.4	-0.3	0.5	-0.6	-	-

Supplementary Table 2 | Performance comparison of DRM catalysts.

Catalyst	Flow	Temp. (°C)	Rate ($mol\ g_{cat}^{-1}\ h^{-1}$)	Coke formation	Reference
**$CoAl_{0.5}Ga_{1.5}O_4$-R**	**300 $L\ g_{cat.}^{-1}\ h^{-1}$**<br>**CH_4:CO_2:He=1:1:8**	**700**	**CH_4: 0.51**<br>**CO_2: 0.70**	**1000 h**<br>**coke-free**	**This work**
Mo doped Ni on single crystal MgO[b]	60 $L\ g_{cat.}^{-1}\ h^{-1}$<br>CH_4:CO_2:He=1:1:8	800	CH_4: ~0.27<br>CO_2: ~0.27	850 h<br>coke-free	(*3*)
Ni atomically dispersed over CeOx doped hydroxyapatite	60 $L\ g_{cat.}^{-1}\ h^{-1}$<br>CH_4:CO_2:He=1:1:3	750	CH_4: 196.4<br>CO_2: 330.1	100 h<br>~2% weight loss by TGA	(*4*)
Ni@BOx/h-BN	60 $L\ g_{cat.}^{-1}\ h^{-1}$<br>CH_4:CO_2:N_2=2:2:1	750	CH_4: 0.78<br>CO_2: 0.86	40 h<br>No coke from TEM&SEM	*(5)*
Multielement oxide layer confined Ni	30 $L\ g_{cat.}^{-1}\ h^{-1}$<br>CH_4:CO_2:N_2=1:1:3	800	CH_4: ~0.19 to 0.21<br>CO_2: ~0.20 to 0.22	300 h<br>coke-free	(*6*)
Ni/ZrO_2@BN	25 $L\ g_{cat.}^{-1}\ h^{-1}$<br>CH_4:CO_2=1:1	750	CH_4: ~0.39<br>CO_2: ~0.45	200 h<br>2.8 wt.% coke	(*7*)
Ni/$Ce_{0.9}Eu_{0.1}O_{1.95}$	60 $L\ g_{cat.}^{-1}\ h^{-1}$<br>CH_4:CO_2:N_2=1:1:2	600	CH_4: 0.16<br>CO_2: 0.23	700 min<br>18.7% weight loss from TGA	(*8*)
Ni@HZSM-5	690 $L\ g_{cat.}^{-1}\ h^{-1}$<br>33.0%CO_2/10.6%CH_4/2.6% Ar/53.8% He	500	CO formation:<br>0.348 $mol_{CO}\ g_{Ni}^{-1}\ h^{-1}$	20 h<br>~0.43 wt.% coke	(*9*)

Reference:

1. A. P. E. York, T. c. Xiao, M. L. H. Green, J. B. Claridge, Methane Oxyforming for Synthesis Gas Production. *Catalysis Reviews* **49**, 511-560 (2007).
2. C. Jensen, M. S. Duyar, Thermodynamic Analysis of Dry Reforming of Methane for Valorization of Landfill Gas and Natural Gas. *Energy Technology* **9**, 2100106 (2021).
3. Y. Song *et al.*, Dry Reforming of Methane by Stable Ni-Mo Nanocatalysts on Single-crystalline MgO. *Science* **367**, 777-781 (2020).
4. M. Akri *et al.*, Atomically dispersed nickel as coke-resistant active sites for methane dry reforming. *Nat Commun* **10**, 5181 (2019).
5. J. Dong *et al.*, Reaction-Induced Strong Metal-Support Interactions between Metals and Inert Boron Nitride Nanosheets. *J. Am. Chem. Soc.* **142**, 17167-17174 (2020).
6. L. He *et al.*, Robust and Coke-free Ni Catalyst Stabilized by 1–2 nm-Thick Multielement Oxide for Methane Dry Reforming. *ACS Catal.* **11**, 12409-12416 (2021).
7. J. Deng *et al.*, Cooperatively enhanced coking resistance via boron nitride coating over Ni-based catalysts for dry reforming of methane. *Appl. Catal., B* **302**, (2022).
8. Y. Wang, R. Zhang, B. Yan, Ni/Ce0.9Eu0.1O1.95 with enhanced coke resistance for dry reforming of methane. *Journal of Catalysis* **407**, 77-89 (2022).
9. Q. Zhu *et al.*, Enhanced CO2 utilization in dry reforming of methane achieved through nickel-mediated hydrogen spillover in zeolite crystals. *Nat. Catal.* **5**, 1030-1037 (2022).
