# Supplementary material for: Balancing elementary steps enables coke-free dry reforming of methane
Source: Nat Commun. 2023 Nov 18;14:7514. doi: 10.1038/s41467-023-43277-0 (PMC10657353; doi:10.1038/s41467-023-43277-0)
Supplement: Supplementary file 3 — Description of Additional Supplementary Files [file 41467_2023_43277_MOESM3_ESM.pdf]

Description of Additional Supplementary Files

File Name: Supplementary Data 1
Description: Optimized configurations of adsorbed species on Co (111), CoGa (111) and $CoGa_3$(111).
