# Supplementary material for: Balancing elementary steps enables coke-free dry reforming of methane
Source: Nat Commun. 2023 Nov 18;14:7514. doi: 10.1038/s41467-023-43277-0 (PMC10657353; doi:10.1038/s41467-023-43277-0)
Supplement: Supplementary file 4 — Supplementary Data 1 [file 41467_2023_43277_MOESM4_ESM.pdf]

Supplementary Data 1

Optimized configurations of adsorbed species on Co (111), CoGa (111) and $CoGa_3$(111)

A. Co (111)

1. *CH_4 on Co (111)*
2. *CH_3* and H* on Co(111)*
3. *CH_3* on Co(111)*
4. *CH_2* and H* on Co(111)*
5. *CH_2* on Co(111)*
6. *CH* and H* on Co(111)*
7. *CH* on Co(111)*
8. *C* and H* on Co(111)*
9. *CO_2 on Co(111)*
10. *CO* and O* on Co(111)*
11. *C* and CO_2 on Co(111)*
12. *2CO* on Co(111)*
13. *C-C-C and CO_2 on Co(111)*
14. *C-C and 2CO on Co(111)*

B. CoGa(111)

1. *CH_4 on CoGa*
2. *CH_3* and H* on CoGa*
3. *CH_3* on CoGa*
4. *CH_2* and H* on CoGa*
5. *CH_2* on CoGa*
6. *CH* and H* on CoGa*
7. *CH* on CoGa*
8. *C* and H* on CoGa*
9. *CO_2 on CoGa*
10. *CO* and O* on CoGa*
11. *C* and CO_2 on CoGa*
12. *2CO* on CoGa*
13. *C-C-C* and CO_2 on CoGa*

14. C-C and 2CO* on CoGa*

C. $CoGa_3$(111)

1. *CH_4 on $CoGa_3$*
2. *CH_3* and H* on $CoGa_3$*
3. *CH_3* on $CoGa_3$*
4. *CH_2* and H* on $CoGa_3$*
5. *CH_2* on $CoGa_3$*
6. *CH* and H* on $CoGa_3$*
7. *CH* on $CoGa_3$*
8. *C* and H* on $CoGa_3$*

Optimized configurations of adsorbed species on Co (111), CoGa (111) and $CoGa_3$(111)

A. Co (111)

1. CH_4 on Co (111)

```
C Co H
1.0
  7.386800000000 -12.794312905350   0.000000000000
  7.386800000000  12.794312905350   0.000000000000
  0.000000000000   0.000000000000  21.031300000000
C Co H
  1 144   4
Selective dynamics
Direct
  0.517885566711   0.545249954810   0.435261090309
  0.111115756604   0.055514405947   0.188008573661
  0.000008618374   0.166662721503   0.280064592873
  0.277747935159   0.055548148304   0.188023206131
  0.166680745476   0.166675645551   0.280087262401
  0.277764068409   0.222164021121   0.187980384531
  0.166656977680   0.000001567445   0.280104440955
 -0.000021216685  -0.000018613305   0.280091160305
  0.444416908015   0.222215482226   0.187978084043
  0.444372327689   0.055540290051   0.188003453074
  0.333348158899   0.166659272458   0.280115381663
  0.611091727256   0.055553092409   0.188013483124
  0.499993220309   0.166719679625   0.280078957617
  0.611094513641   0.222217694963   0.187987524540
  0.333218141820   0.333301124590   0.279996675917
  0.333304656514   0.000003591101   0.280101104586
  0.833289434554   0.666646481210   0.280091420204
  0.111094856042   0.222183881606   0.187977857802
```

0.777771424152	0.222219888334	0.187969420643
0.833331874138	0.166651378360	0.280064614275
0.944465787398	0.222210719116	0.187990772952
0.833320226038	-0.000010581208	0.280083945101
0.111076063304	0.555578677058	0.187988045119
0.777760331431	0.055547908262	0.187982319180
0.000002754436	0.333339153869	0.280050727077
0.499992083638	0.000030566263	0.280093764813
-0.000035778119	0.499948548689	0.280075231050
0.166674577931	0.500032186083	0.280072828099
0.277772703778	0.555609922360	0.187953290661
0.166680992093	0.333309641012	0.280090782815
0.444483999366	0.555569980836	0.187967159095
0.111072198958	0.388865672605	0.187979121754
0.666669138967	0.000034277119	0.280072162101
0.277785660189	0.388878679521	0.187936632783
0.444522365310	0.389065030005	0.188057560299
0.611108388827	0.388904481033	0.187969860531
0.444404037448	0.888920238359	0.188019360928
0.500038208210	0.333317010800	0.280041258408
0.777826260370	0.555588419801	0.187977851133
0.666663582856	0.333346154035	0.280082711178
0.166647908063	0.833304465520	0.280102105838
0.666773473417	0.500018588730	0.279978904486
0.777789351390	0.388862557840	0.187973561077
0.610939415530	0.555529027998	0.188033294212
0.833281495589	0.499979947855	0.280081026934
0.833310911522	0.333326811590	0.280048705210
0.111122833166	0.888887032995	0.188053520034
-0.000026633975	0.666622937440	0.280086737575
0.111109685493	0.722247224886	0.188038186703
-0.000016014645	0.833312766162	0.280100694040
0.944426208273	0.388858930262	0.187989306775
0.944441951184	0.555560503804	0.187988801974
0.499926190336	0.499876581034	0.280218513607
0.277721732889	0.722246535054	0.187973665842
0.166637550128	0.666625301629	0.280079958292
0.333327362527	0.666642897063	0.280092330593
0.444442466440	0.722284257352	0.187981544189
0.333320787956	0.833337670121	0.280100252613
0.611100169507	0.722222753315	0.187936554774
0.499985504262	0.833368002897	0.280057858296
0.333008022145	0.499928858824	0.279923809534
0.611099010692	0.888940992101	0.187988247865
0.777803794993	0.888928112294	0.187991786512
0.666879661441	0.666915718278	0.279982786487

0.777824776182	0.722234064752	0.187987063326
0.666664362628	0.833309465354	0.280067697490
0.944495293642	0.722253961104	0.188007368789
0.833317766455	0.833349388225	0.280084742575
0.500035177198	0.667059664495	0.279948497072
0.277753387284	0.888899453436	0.188046735533
0.944415505450	0.055530022119	0.188004105048
0.944517671937	0.888901184207	0.187998550850
0.666680253205	0.166697612652	0.280072824999
0.000000000000	0.000000000000	0.000000000000
0.166666622887	0.166666622880	0.000000000000
0.055555540978	0.277777704852	0.095592368850
0.166666622895	-0.000000000000	0.000000000000
0.055555540986	0.111111081972	0.095592368850
0.333333245790	-0.000000000000	0.000000000000
0.666666491640	0.166666622880	0.000000000000
0.222222163873	0.277777704852	0.095592368850
0.555555409738	0.111111081972	0.095592368850
0.499999868745	0.166666622880	0.000000000000
0.388888786768	0.277777704852	0.095592368850
0.499999868752	-0.000000000000	0.000000000000
0.388888786776	0.111111081972	0.095592368850
0.666666491647	-0.000000000000	0.000000000000
0.333333245782	0.166666622880	0.000000000000
0.555555409731	0.277777704852	0.095592368850
0.888888655528	0.111111081972	0.095592368850
0.888888655520	0.277777704852	0.095592368850
0.555555409687	0.611110950689	0.095592368850
0.722222032626	0.277777704852	0.095592368850
0.833333114542	-0.000000000000	0.000000000000
0.722222032633	0.111111081972	0.095592368850
0.000000000024	0.333333245837	0.000000000000
0.222222163866	0.444444327731	0.095592368850
0.000000000016	0.499999868717	0.000000000000
0.833333114535	0.166666622880	0.000000000000
0.222222163897	0.611110950689	0.095592368850
0.055555541002	0.611110950689	0.095592368850
0.166666622919	0.333333245837	0.000000000000
0.055555540971	0.444444327731	0.095592368850
0.333333245814	0.333333245837	0.000000000000
0.555555409655	0.444444327731	0.095592368850
0.333333245806	0.499999868717	0.000000000000
0.166666622911	0.499999868717	0.000000000000
0.499999868701	0.499999868717	0.000000000000
0.222222163881	0.111111081972	0.095592368850
0.388888786792	0.611110950689	0.095592368850

0.333333245830	0.833333114555	0.000000000000
0.888888655513	0.444444327731	0.095592368850
0.666666491663	0.499999868717	0.000000000000
0.888888655544	0.611110950689	0.095592368850
0.833333114558	0.499999868717	0.000000000000
0.722222032649	0.611110950689	0.095592368850
0.833333114566	0.333333245837	0.000000000000
0.499999868709	0.333333245837	0.000000000000
0.722222032618	0.444444327731	0.095592368850
0.222222163889	0.777777573569	0.095592368850
0.999999999972	0.833333114555	0.000000000000
0.222222163921	0.944444196526	0.095592368850
0.166666622935	0.833333114555	0.000000000000
0.055555540958	0.944444196526	0.095592368850
0.999999999992	0.166666622880	0.000000000000
0.000000000048	0.666666491675	0.000000000000
0.388888786760	0.444444327731	0.095592368850
0.666666491671	0.333333245837	0.000000000000
0.055555540994	0.777777573569	0.095592368850
0.555555409711	0.944444196526	0.095592368850
0.499999868725	0.833333114555	0.000000000000
0.388888786816	0.944444196526	0.095592368850
0.499999868732	0.666666491675	0.000000000000
0.388888786784	0.777777573569	0.095592368850
0.166666622943	0.666666491675	0.000000000000
0.666666491627	0.666666491675	0.000000000000
0.555555409679	0.777777573569	0.095592368850
0.888888655568	0.944444196526	0.095592368850
0.833333114582	0.833333114555	0.000000000000
0.722222032606	0.944444196526	0.095592368850
0.833333114590	0.666666491675	0.000000000000
0.722222032574	0.777777573569	0.095592368850
0.888888655537	0.777777573569	0.095592368850
0.333333245837	0.666666491675	0.000000000000
0.666666491620	0.833333114555	0.000000000000
0.570793065985	0.517251356810	0.420292626219
0.446918465518	0.508069745199	0.405769034849
0.496306480013	0.525157538399	0.485289194763
0.556906495752	0.630246507170	0.428882478356

2. CH_3* and H* on Co(111)

C Co H

1.0

7.386800000000	-12.794312905350	0.000000000000
7.386800000000	12.794312905350	0.000000000000
0.000000000000	0.000000000000	21.031300000000

```
C Co H
  1 144   4
Selective dynamics
Direct
  0.437443257868   0.548677221469   0.352987768705
  0.110966203460   0.055238402877   0.187796437745
  0.000183712530   0.166338341272   0.280042054214
  0.277673345754   0.055251854672   0.187854795336
  0.166266708501   0.166059994257   0.279850643822
  0.277747161863   0.222222951185   0.187461042394
  0.166514799789   0.000422369230   0.279939499013
  0.000018602820   0.000339194691   0.279974203439
  0.444439289307   0.222279976436   0.188002665204
  0.444346361193   0.055540554458   0.187826385605
  0.332967109998   0.166085287524   0.279827795113
  0.611001936710   0.055566356631   0.187972476996
  0.499824076148   0.166220605958   0.280100328938
  0.611004221299   0.222200476656   0.187985770680
  0.330857739056   0.328472194217   0.278726069966
  0.333374805097   0.000001916787   0.280055356680
  0.833837750532   0.666993185925   0.279799201355
  0.111091884397   0.222278886766   0.187976407507
  0.777660857910   0.222157128743   0.188040585371
  0.833255863423   0.166848514134   0.279999190186
  0.944411588936   0.222150933043   0.187952053591
  0.833629523365   0.000331107748   0.279952764969
  0.111010262191   0.555473111081   0.188000730850
  0.777755102833   0.055531043168   0.187942213631
  0.000352820050   0.333180397721   0.280001259801
  0.499843204014   0.000455221769   0.279963101404
  0.000546506220   0.500123206952   0.280075167779
  0.164995278588   0.499295376372   0.279734863730
  0.277991310224   0.555277299060   0.188340907688
  0.165168103866   0.332498064077   0.279715983094
  0.444633362320   0.555813254844   0.187832765688
  0.111174327437   0.388931609322   0.187778350976
  0.666757037240   0.000213939280   0.279910155553
  0.277977086076   0.389242597411   0.188193139479
  0.444776788823   0.389558778176   0.188442469169
  0.611037792091   0.388964241887   0.187856958734
  0.444526169407   0.888765727832   0.188015858067
  0.500403499406   0.332627715324   0.279768534623
  0.777628205160   0.555522169104   0.188005359452
  0.666636926309   0.333225376984   0.280028644121
  0.166578975127   0.833470880735   0.280004829742
  0.666915554052   0.499544746554   0.279802939022
```

0.777721909389	0.388876402157	0.187982566510
0.610416705727	0.555395508676	0.188494162900
0.833509667642	0.500031823710	0.280012673772
0.833178374451	0.333151119010	0.280092640754
0.111185249575	0.888873717492	0.187763500533
-0.000006585557	0.666309137127	0.280039091818
0.110977232930	0.722300123711	0.187821323252
0.000533397285	0.833416606749	0.279892129658
0.944374059606	0.388888101643	0.188040389528
0.944363239711	0.555570301197	0.187826129848
0.499894142572	0.498767124396	0.283264886051
0.277789467758	0.722042267789	0.187646185066
0.166365386243	0.666959756780	0.279855357420
0.331614522665	0.668581696703	0.279086091866
0.445050236156	0.722003453401	0.188508234736
0.332953350349	0.833273889831	0.279833964773
0.610550974993	0.722096947910	0.188312682455
0.499797402085	0.834707957682	0.279767867739
0.330636139769	0.498968529794	0.282563426216
0.610995514769	0.888721575820	0.187680248416
0.777767739674	0.888696342633	0.187913929999
0.670974102829	0.667338912955	0.280324045811
0.777519348588	0.722165733725	0.187589716298
0.668330550389	0.837279634204	0.280558320463
0.944644167188	0.722277694968	0.187852890792
0.835337686290	0.834067272005	0.279662257727
0.498812926668	0.665694380605	0.285169980947
0.277722142102	0.888958363646	0.187846396768
0.944451165709	0.055559992116	0.187830097632
0.944509974120	0.888933185117	0.187866771162
0.666698617065	0.166973355824	0.280074951570
0.000000000000	0.000000000000	0.000000000000
0.166666622887	0.166666622880	0.000000000000
0.055555540978	0.277777704852	0.095592368850
0.166666622895	-0.000000000000	0.000000000000
0.055555540986	0.111111081972	0.095592368850
0.333333245790	-0.000000000000	0.000000000000
0.666666491640	0.166666622880	0.000000000000
0.222222163873	0.277777704852	0.095592368850
0.555555409738	0.111111081972	0.095592368850
0.499999868745	0.166666622880	0.000000000000
0.388888786768	0.277777704852	0.095592368850
0.499999868752	-0.000000000000	0.000000000000
0.388888786776	0.111111081972	0.095592368850
0.666666491647	-0.000000000000	0.000000000000
0.333333245782	0.166666622880	0.000000000000

0.555555409731	0.277777704852	0.095592368850
0.888888655528	0.111111081972	0.095592368850
0.888888655520	0.277777704852	0.095592368850
0.555555409687	0.611110950689	0.095592368850
0.722222032626	0.277777704852	0.095592368850
0.833333114542	-0.000000000000	0.000000000000
0.722222032633	0.111111081972	0.095592368850
0.000000000024	0.333333245837	0.000000000000
0.222222163866	0.444444327731	0.095592368850
0.000000000016	0.499999868717	0.000000000000
0.833333114535	0.166666622880	0.000000000000
0.222222163897	0.611110950689	0.095592368850
0.055555541002	0.611110950689	0.095592368850
0.166666622919	0.333333245837	0.000000000000
0.055555540971	0.444444327731	0.095592368850
0.333333245814	0.333333245837	0.000000000000
0.555555409655	0.444444327731	0.095592368850
0.333333245806	0.499999868717	0.000000000000
0.166666622911	0.499999868717	0.000000000000
0.499999868701	0.499999868717	0.000000000000
0.222222163881	0.111111081972	0.095592368850
0.388888786792	0.611110950689	0.095592368850
0.333333245830	0.833333114555	0.000000000000
0.888888655513	0.444444327731	0.095592368850
0.666666491663	0.499999868717	0.000000000000
0.888888655544	0.611110950689	0.095592368850
0.833333114558	0.499999868717	0.000000000000
0.722222032649	0.611110950689	0.095592368850
0.833333114566	0.333333245837	0.000000000000
0.499999868709	0.333333245837	0.000000000000
0.722222032618	0.444444327731	0.095592368850
0.222222163889	0.777777573569	0.095592368850
0.999999999972	0.833333114555	0.000000000000
0.222222163921	0.944444196526	0.095592368850
0.166666622935	0.833333114555	0.000000000000
0.055555540958	0.944444196526	0.095592368850
0.999999999992	0.166666622880	0.000000000000
0.000000000048	0.666666491675	0.000000000000
0.388888786760	0.444444327731	0.095592368850
0.666666491671	0.333333245837	0.000000000000
0.055555540994	0.777777573569	0.095592368850
0.555555409711	0.944444196526	0.095592368850
0.499999868725	0.833333114555	0.000000000000
0.388888786816	0.944444196526	0.095592368850
0.499999868732	0.666666491675	0.000000000000
0.388888786784	0.777777573569	0.095592368850

```
  0.166666622943   0.666666491675   0.000000000000
  0.666666491627   0.666666491675   0.000000000000
  0.555555409679   0.777777573569   0.095592368850
  0.888888655568   0.944444196526   0.095592368850
  0.833333114582   0.833333114555   0.000000000000
  0.722222032606   0.944444196526   0.095592368850
  0.833333114590   0.666666491675   0.000000000000
  0.722222032574   0.777777573569   0.095592368850
  0.888888655537   0.777777573569   0.095592368850
  0.333333245837   0.666666491675   0.000000000000
  0.666666491620   0.833333114555   0.000000000000
  0.477292799519   0.505618600209   0.372195401516
  0.352001143596   0.500743202780   0.368844296438
  0.471077683335   0.623856404420   0.379810264757
  0.618979468824   0.728165810712   0.323977435285
```

3. CH_3* on Co(111)

```
C Co H
1.0
  7.386800000000 -12.794312905350   0.000000000000
  7.386800000000  12.794312905350   0.000000000000
  0.000000000000   0.000000000000  21.031300000000
C Co H
  1 144   3
Selective dynamics
Direct
  0.444179568216   0.555268759766   0.351609753007
  0.277713307333   0.055358775933   0.187854301780
  0.166511962216   0.166265226122   0.279911008597
  0.277815536842   0.222316181097   0.187601326027
  0.166499476762   0.999685861056   0.279956227476
  0.666698332538   0.000079984899   0.280033967234
  0.444504198432   0.222303666824   0.188043329275
  0.999626841267   0.166172732018   0.280076720613
  0.444432138193   0.055538764485   0.187835076193
  0.611068464986   0.055514272036   0.187966219797
  0.499855622806   0.166184076199   0.280074612626
  0.611069492667   0.222199573052   0.187968827610
  0.499866736988   0.000271752587   0.280077384266
  0.333533317414   0.000076229486   0.280075836075
  0.332025746535   0.499330575936   0.282932913092
  0.333076705221   0.166274309577   0.279909619909
  0.611049331754   0.388907633552   0.187859486431
  0.110953151817   0.055353665463   0.187854462414
  0.777757630653   0.055517483451   0.187965812630
  0.944451688777   0.222203880466   0.187970528901
```

0.833692519170	0.000260864034	0.280079018500
0.111114011851	0.555456471561	0.188042648084
-0.000137806727	0.333241769007	0.280039196594
0.666813262470	0.166717379276	0.280067960356
0.111180432667	0.388907569942	0.187859413677
0.777755269318	0.222192460843	0.188012262168
0.278031963905	0.389400414448	0.188383640408
0.278033020703	0.555281220713	0.188387877962
0.165663092255	0.332854465145	0.279857850317
0.444427892519	0.555535653522	0.187574363065
0.331285123661	0.329250576748	0.279069428574
0.999647034844	0.500071269034	0.280074084835
0.833662979994	0.666875507780	0.279910583267
0.165666635752	0.499411521733	0.279859544069
0.500577946516	0.499365197414	0.282961282574
0.331303478965	0.668643472095	0.279081245206
0.444702230788	0.721923836767	0.188400083777
0.666703012768	0.333237720636	0.280036042812
0.777752259167	0.388875573680	0.187967536521
0.667027491947	0.499409191691	0.279864415432
0.944444583929	0.388877198840	0.187969391480
0.833704810317	0.500062279976	0.280072837128
0.944417769370	0.555517178227	0.187829473796
0.777658770412	0.555461172693	0.188043964063
0.833216332227	0.333111201458	0.280068409653
0.999830821595	0.666354149609	0.280070654295
0.110950570167	0.722237349746	0.187853309457
0.000226904802	0.833428970110	0.279946404014
0.277816812772	0.722145239172	0.187602572037
0.999842725553	0.000083851301	0.280074082365
0.111110075103	0.222291365658	0.188042952969
0.111093298183	0.888870569453	0.187804539288
0.500510468502	0.332886162441	0.279860427810
0.610555455324	0.555275410541	0.188396805459
0.166495028435	0.833422493717	0.279949171427
0.333071986241	0.833425270513	0.279908551203
0.610542476352	0.721920240058	0.188405068974
0.500509698092	0.834212541712	0.279869642265
0.611052750631	0.888787917258	0.187862162625
0.444501735951	0.888840729475	0.188045901750
0.500523710437	0.667719464393	0.282992592567
0.670633901762	0.668632773793	0.279093627422
0.777644178912	0.722145393948	0.187607512194
0.666997155407	0.834205294474	0.279871395999
0.944602411384	0.722239048423	0.187853187452
0.833651067133	0.833419953685	0.279910601962

0.944595173129	0.888995463652	0.187852494040
0.777651448858	0.888837920129	0.188044746777
0.166519246596	0.666873406539	0.279909056558
0.277711349510	0.888996016669	0.187853009153
0.833225455551	0.166713533534	0.280069100278
0.944418093389	0.055534621693	0.187833268182
0.444688795666	0.389407654674	0.188386185803
0.388888786792	0.611110950689	0.095592368850
0.055555540978	0.277777704852	0.095592368850
0.166666622895	-0.000000000000	0.000000000000
0.055555540986	0.111111081972	0.095592368850
0.333333245790	-0.000000000000	0.000000000000
0.666666491640	0.166666622880	0.000000000000
0.555555409738	0.111111081972	0.095592368850
0.166666622887	0.166666622880	0.000000000000
0.555555409731	0.277777704852	0.095592368850
0.388888786768	0.277777704852	0.095592368850
0.499999868752	-0.000000000000	0.000000000000
0.388888786776	0.111111081972	0.095592368850
0.666666491647	-0.000000000000	0.000000000000
0.333333245782	0.166666622880	0.000000000000
0.499999868701	0.499999868717	0.000000000000
0.499999868745	0.166666622880	0.000000000000
0.000000000000	0.000000000000	0.000000000000
0.222222163873	0.277777704852	0.095592368850
0.888888655520	0.277777704852	0.095592368850
0.722222032633	0.111111081972	0.095592368850
0.000000000024	0.333333245837	0.000000000000
0.222222163866	0.444444327731	0.095592368850
0.000000000016	0.499999868717	0.000000000000
0.833333114535	0.166666622880	0.000000000000
0.222222163897	0.611110950689	0.095592368850
0.888888655528	0.111111081972	0.095592368850
0.055555541002	0.611110950689	0.095592368850
0.055555540971	0.444444327731	0.095592368850
0.333333245814	0.333333245837	0.000000000000
0.555555409655	0.444444327731	0.095592368850
0.333333245806	0.499999868717	0.000000000000
0.166666622911	0.499999868717	0.000000000000
0.555555409687	0.611110950689	0.095592368850
0.166666622919	0.333333245837	0.000000000000
0.499999868709	0.333333245837	0.000000000000
0.333333245830	0.833333114555	0.000000000000
0.555555409711	0.944444196526	0.095592368850
0.666666491663	0.499999868717	0.000000000000
0.888888655544	0.611110950689	0.095592368850

0.833333114558   0.499999868717   0.000000000000
0.722222032649   0.611110950689   0.095592368850
0.833333114566   0.333333245837   0.000000000000
0.722222032618   0.444444327731   0.095592368850
0.888888655513   0.444444327731   0.095592368850
0.000000000048   0.666666491675   0.000000000000
0.999999999972   0.833333114555   0.000000000000
0.222222163921   0.944444196526   0.095592368850
0.166666622935   0.833333114555   0.000000000000
0.055555540958   0.944444196526   0.095592368850
0.999999999992   0.166666622880   0.000000000000
0.222222163881   0.111111081972   0.095592368850
0.222222163889   0.777777573569   0.095592368850
0.666666491671   0.333333245837   0.000000000000
0.388888786760   0.444444327731   0.095592368850
0.166666622943   0.666666491675   0.000000000000
0.499999868725   0.833333114555   0.000000000000
0.388888786816   0.944444196526   0.095592368850
0.499999868732   0.666666491675   0.000000000000
0.388888786784   0.777777573569   0.095592368850
0.555555409679   0.777777573569   0.095592368850
0.666666491627   0.666666491675   0.000000000000
0.666666491620   0.833333114555   0.000000000000
0.888888655568   0.944444196526   0.095592368850
0.833333114582   0.833333114555   0.000000000000
0.722222032606   0.944444196526   0.095592368850
0.833333114590   0.666666491675   0.000000000000
0.722222032574   0.777777573569   0.095592368850
0.888888655537   0.777777573569   0.095592368850
0.333333245837   0.666666491675   0.000000000000
0.055555540994   0.777777573569   0.095592368850
0.833333114542 -0.000000000000   0.000000000000
0.722222032626   0.277777704852   0.095592368850
0.484844544766   0.514040479121   0.372181267553
0.362069566190   0.514100433974   0.371936081083
0.484932858470   0.636904354003   0.372466079829

4. CH_2* and H* on Co(111)

C Co H
1.0
7.386800000000 -12.794312905350   0.000000000000
7.386800000000 12.794312905350   0.000000000000
0.000000000000   0.000000000000 21.031300000000
C Co H
1 144   3
Selective dynamics

Direct

0.439251483786	0.550311368150	0.341931559562
0.277750868301	0.055403008255	0.187885423326
0.166046324109	0.166074756454	0.279955146500
0.277908201310	0.222429214044	0.187850023855
0.166640712362	0.999811580522	0.279924310656
0.666933039940	0.000401468768	0.279858025853
0.444590642183	0.222317691056	0.188052195897
0.999845726230	0.166494888116	0.280094881801
0.444405559587	0.055461197810	0.187808764282
0.611073388352	0.055553733530	0.187933118253
0.500016182184	0.166030636236	0.280134443727
0.611100787738	0.222271008205	0.187953003672
0.500016538874	0.000421186480	0.279995357323
0.333375417633	0.000017098706	0.280030078050
0.328412951798	0.497500057692	0.282880236371
0.333056161765	0.165976775653	0.279946985933
0.611021619553	0.389130313053	0.187949049631
0.111047896265	0.055441265277	0.187842556664
0.777774048559	0.055550146227	0.187887435237
0.944492832273	0.222214883113	0.187901509074
0.833656701272	0.000541244271	0.279955541843
0.111133306988	0.555651717147	0.187972195879
0.000335216110	0.333183382310	0.279911683507
0.666715434076	0.166815926788	0.280074028871
0.111332745117	0.389088586835	0.187884452037
0.777740817783	0.222276379886	0.188042077136
0.278414875922	0.389163719903	0.188018989130
0.278479804250	0.556561594336	0.187832155871
0.165819821282	0.333071596824	0.280007246226
0.444302151034	0.556225265450	0.187139995077
0.330727157253	0.329058692909	0.279493871660
0.999599993190	0.500029887822	0.280074700824
0.833856007183	0.666917314314	0.279809889520
0.166365819476	0.499716165482	0.279864207308
0.500245370269	0.497332442084	0.282791969163
0.331613403666	0.668380578212	0.279386344826
0.444259542678	0.721522265805	0.188282088057
0.666739072809	0.333074542224	0.280020992284
0.777769132496	0.388956432806	0.187969025162
0.668115614725	0.499948803825	0.279793843487
0.944477498052	0.388958924010	0.188031596597
0.833828517644	0.500091335077	0.280027960769
0.944502202956	0.555626177493	0.187830507973
0.777529110534	0.555481859944	0.188039055349
0.833416028245	0.333356949408	0.280093273370

0.999765627490	0.666615709671	0.280083096706
0.111009491842	0.722312864080	0.187843705266
0.000562463769	0.833539104694	0.279874604127
0.277704839953	0.722351346011	0.187846540717
0.999875303480	0.000310494513	0.280008044610
0.111122021498	0.222285498095	0.187964422894
0.111167887033	0.888948213824	0.187760438553
0.500111363794	0.331669719885	0.279762837939
0.610254827371	0.555755981128	0.188800203200
0.166552886840	0.833461904381	0.279994048569
0.333280796466	0.833693042944	0.279930015140
0.610771974794	0.721810351054	0.188156005717
0.499592323736	0.834130077927	0.279871937826
0.610859959327	0.888607164581	0.187821172358
0.444528762787	0.888737257400	0.188070434373
0.500121241758	0.668285828597	0.285576843280
0.671359836114	0.668464498858	0.280670130995
0.777290345466	0.722109435093	0.187852810322
0.668030813437	0.836503986993	0.280845246083
0.944594142466	0.722261328424	0.187860602141
0.835477038472	0.834572965254	0.279743498010
0.944440068427	0.888914407691	0.187854855503
0.777539852786	0.888717469772	0.187977751897
0.166085108187	0.666681748205	0.279942625211
0.277770783409	0.889004344187	0.187870760273
0.833313578513	0.166902282314	0.279974009214
0.944426492194	0.055541558692	0.187922696103
0.444403317101	0.389412778197	0.188654977991
0.388888786792	0.611110950689	0.095592368850
0.055555540978	0.277777704852	0.095592368850
0.166666622895	-0.000000000000	0.000000000000
0.055555540986	0.111111081972	0.095592368850
0.333333245790	-0.000000000000	0.000000000000
0.666666491640	0.166666622880	0.000000000000
0.555555409738	0.111111081972	0.095592368850
0.166666622887	0.166666622880	0.000000000000
0.555555409731	0.277777704852	0.095592368850
0.388888786768	0.277777704852	0.095592368850
0.499999868752	-0.000000000000	0.000000000000
0.388888786776	0.111111081972	0.095592368850
0.666666491647	-0.000000000000	0.000000000000
0.333333245782	0.166666622880	0.000000000000
0.499999868701	0.499999868717	0.000000000000
0.499999868745	0.166666622880	0.000000000000
0.000000000000	0.000000000000	0.000000000000
0.222222163873	0.277777704852	0.095592368850

0.888888655520	0.277777704852	0.095592368850
0.722222032633	0.111111081972	0.095592368850
0.000000000024	0.333333245837	0.000000000000
0.222222163866	0.444444327731	0.095592368850
0.000000000016	0.499999868717	0.000000000000
0.833333114535	0.166666622880	0.000000000000
0.222222163897	0.611110950689	0.095592368850
0.888888655528	0.111111081972	0.095592368850
0.055555541002	0.611110950689	0.095592368850
0.055555540971	0.444444327731	0.095592368850
0.333333245814	0.333333245837	0.000000000000
0.555555409655	0.444444327731	0.095592368850
0.333333245806	0.499999868717	0.000000000000
0.166666622911	0.499999868717	0.000000000000
0.555555409687	0.611110950689	0.095592368850
0.166666622919	0.333333245837	0.000000000000
0.499999868709	0.333333245837	0.000000000000
0.333333245830	0.833333114555	0.000000000000
0.555555409711	0.944444196526	0.095592368850
0.666666491663	0.499999868717	0.000000000000
0.888888655544	0.611110950689	0.095592368850
0.833333114558	0.499999868717	0.000000000000
0.722222032649	0.611110950689	0.095592368850
0.833333114566	0.333333245837	0.000000000000
0.722222032618	0.444444327731	0.095592368850
0.888888655513	0.444444327731	0.095592368850
0.000000000048	0.666666491675	0.000000000000
0.999999999972	0.833333114555	0.000000000000
0.222222163921	0.944444196526	0.095592368850
0.166666622935	0.833333114555	0.000000000000
0.055555540958	0.944444196526	0.095592368850
0.999999999992	0.166666622880	0.000000000000
0.222222163881	0.111111081972	0.095592368850
0.222222163889	0.777777573569	0.095592368850
0.666666491671	0.333333245837	0.000000000000
0.388888786760	0.444444327731	0.095592368850
0.166666622943	0.666666491675	0.000000000000
0.499999868725	0.833333114555	0.000000000000
0.388888786816	0.944444196526	0.095592368850
0.499999868732	0.666666491675	0.000000000000
0.388888786784	0.777777573569	0.095592368850
0.555555409679	0.777777573569	0.095592368850
0.666666491627	0.666666491675	0.000000000000
0.666666491620	0.833333114555	0.000000000000
0.888888655568	0.944444196526	0.095592368850
0.833333114582	0.833333114555	0.000000000000

0.722222032606   0.944444196526   0.095592368850
0.833333114590   0.666666491675   0.000000000000
0.722222032574   0.777777573569   0.095592368850
0.888888655537   0.777777573569   0.095592368850
0.333333245837   0.666666491675   0.000000000000
0.055555540994   0.777777573569   0.095592368850
0.833333114542 -0.000000000000   0.000000000000
0.722222032626   0.277777704852   0.095592368850
0.488251211001   0.506697983169   0.358187220296
0.420152805427   0.564849516499   0.390166869964
0.615803117351   0.728001690084   0.325949148008

5. **CH_2* on Co(111)**

C Co H
1.0
7.386800000000 -12.794312905350   0.000000000000
7.386800000000 12.794312905350   0.000000000000
0.000000000000   0.000000000000 21.031300000000
C Co H
1 144   2
Selective dynamics
Direct
0.443353210331   0.556428781732   0.341108976209
0.277702554895   0.055375151682   0.187878257209
0.166086084146   0.166013435502   0.279923475979
0.277922231911   0.222479335385   0.187899154643
0.166463090526   0.999669685348   0.279959088184
0.666796953672   0.000236237559   0.279913378733
0.444572583598   0.222417313667   0.188094275660
0.999577897495   0.166255901562   0.280085283069
0.444385753945   0.055472390227   0.187866743731
0.611046470299   0.055487787737   0.187998152982
0.499940337580   0.166132526529   0.280085203827
0.611086751025   0.222180308858   0.187986140147
0.499955976205   0.000358938019   0.280106520494
0.333342444223   0.999960816040   0.280106035734
0.833972158442   0.666900670242   0.279931545727
0.333041169112   0.165973323402   0.279934799989
0.610980510624   0.388994389476   0.187935697329
0.110933723398   0.055362697674   0.187893562774
0.777755941098   0.055498913977   0.187955569842
0.944475131295   0.222215385763   0.187953848158
0.833638449954   0.000318277540   0.280088901649
0.111071005986   0.555600385238   0.188040366885
0.999708091410   0.333149748158   0.279920950292
0.666710211689   0.166586142343   0.280103283166

0.111301405454	0.389035495778	0.187898447060
0.777762115426	0.222215166352	0.188079702013
0.278430760260	0.389171973183	0.188075676997
0.278422377933	0.556243546726	0.188034810249
0.166183781920	0.333337205583	0.280057482870
0.444027350305	0.555947996573	0.186979775210
0.330826421715	0.329133187500	0.279578777101
0.999597682568	0.500005429284	0.280099288502
0.444533551429	0.389698059946	0.188751419254
0.166294081680	0.499710591331	0.279964713900
0.501086953233	0.498798546045	0.283022014800
0.331469042571	0.668478754659	0.279344904713
0.443755607546	0.721549082069	0.188044525460
0.666796885806	0.333171250705	0.280018873926
0.777790462997	0.388883348408	0.187984879612
0.667946451259	0.499743458990	0.279870992018
0.944485284133	0.388925794166	0.187998929468
0.833815733981	0.500014589079	0.280088516833
0.944486721918	0.555585454847	0.187866692751
0.777547780367	0.555402196347	0.188092548937
0.833373915564	0.333253542921	0.280106715286
0.000006029370	0.666616099682	0.280101659748
0.110946525350	0.722291343705	0.187886625273
0.000269511353	0.833479601971	0.279953848930
0.277586070898	0.722404208142	0.187850528463
0.000120487450	0.000055613859	0.280135423755
0.111092285836	0.222245437231	0.188039918851
0.111075940953	0.888889667022	0.187794007576
0.500238470780	0.332024444721	0.279879251280
0.610294183764	0.555452791680	0.188739305361
0.166509564509	0.833454741489	0.279994456441
0.333349024761	0.833901489213	0.279957474822
0.610751978384	0.721515414692	0.188091311641
0.500237775074	0.833634814670	0.279988669581
0.610934360868	0.888667712029	0.187907889621
0.444375582771	0.888896645437	0.188047504136
0.501976704467	0.671050000858	0.283310789123
0.670819238986	0.669124966762	0.279567271986
0.777480725014	0.722043025790	0.187897246011
0.666570851079	0.833721576528	0.280068470322
0.944590812952	0.722268242056	0.187878645934
0.833947898470	0.833877256466	0.279907247731
0.944595710134	0.889024456919	0.187889793594
0.777692433454	0.888848523076	0.188042400612
0.166063819633	0.666610184111	0.279960567332
0.277683008203	0.889025428598	0.187884933686

0.833253092591	0.166703481245	0.280113599703
0.944400777369	0.055550500320	0.187975103168
0.328856575621	0.497870469741	0.283257936828
0.499999868709	0.333333245837	0.000000000000
0.055555540978	0.277777704852	0.095592368850
0.166666622895	-0.000000000000	0.000000000000
0.055555540986	0.111111081972	0.095592368850
0.333333245790	-0.000000000000	0.000000000000
0.666666491640	0.166666622880	0.000000000000
0.555555409738	0.111111081972	0.095592368850
0.166666622887	0.166666622880	0.000000000000
0.555555409731	0.277777704852	0.095592368850
0.388888786768	0.277777704852	0.095592368850
0.499999868752	-0.000000000000	0.000000000000
0.388888786776	0.111111081972	0.095592368850
0.666666491647	-0.000000000000	0.000000000000
0.333333245782	0.166666622880	0.000000000000
0.499999868701	0.499999868717	0.000000000000
0.499999868745	0.166666622880	0.000000000000
0.388888786792	0.611110950689	0.095592368850
0.222222163873	0.277777704852	0.095592368850
0.888888655520	0.277777704852	0.095592368850
0.722222032633	0.111111081972	0.095592368850
0.000000000024	0.333333245837	0.000000000000
0.222222163866	0.444444327731	0.095592368850
0.000000000016	0.499999868717	0.000000000000
0.833333114535	0.166666622880	0.000000000000
0.222222163897	0.611110950689	0.095592368850
0.888888655528	0.111111081972	0.095592368850
0.055555541002	0.611110950689	0.095592368850
0.055555540971	0.444444327731	0.095592368850
0.333333245814	0.333333245837	0.000000000000
0.555555409655	0.444444327731	0.095592368850
0.333333245806	0.499999868717	0.000000000000
0.166666622911	0.499999868717	0.000000000000
0.555555409687	0.611110950689	0.095592368850
0.166666622919	0.333333245837	0.000000000000
0.000000000000	0.000000000000	0.000000000000
0.333333245830	0.833333114555	0.000000000000
0.555555409711	0.944444196526	0.095592368850
0.666666491663	0.499999868717	0.000000000000
0.888888655544	0.611110950689	0.095592368850
0.833333114558	0.499999868717	0.000000000000
0.722222032649	0.611110950689	0.095592368850
0.833333114566	0.333333245837	0.000000000000
0.722222032618	0.444444327731	0.095592368850

0.888888655513   0.444444327731   0.095592368850
0.000000000048   0.666666491675   0.000000000000
0.999999999972   0.833333114555   0.000000000000
0.222222163921   0.944444196526   0.095592368850
0.166666622935   0.833333114555   0.000000000000
0.055555540958   0.944444196526   0.095592368850
0.999999999992   0.166666622880   0.000000000000
0.222222163881   0.111111081972   0.095592368850
0.222222163889   0.777777573569   0.095592368850
0.666666491671   0.333333245837   0.000000000000
0.388888786760   0.444444327731   0.095592368850
0.166666622943   0.666666491675   0.000000000000
0.499999868725   0.833333114555   0.000000000000
0.388888786816   0.944444196526   0.095592368850
0.499999868732   0.666666491675   0.000000000000
0.388888786784   0.777777573569   0.095592368850
0.555555409679   0.777777573569   0.095592368850
0.666666491627   0.666666491675   0.000000000000
0.666666491620   0.833333114555   0.000000000000
0.888888655568   0.944444196526   0.095592368850
0.833333114582   0.833333114555   0.000000000000
0.722222032606   0.944444196526   0.095592368850
0.833333114590   0.666666491675   0.000000000000
0.722222032574   0.777777573569   0.095592368850
0.888888655537   0.777777573569   0.095592368850
0.333333245837   0.666666491675   0.000000000000
0.055555540994   0.777777573569   0.095592368850
0.833333114542 -0.000000000000   0.000000000000
0.722222032626   0.277777704852   0.095592368850
0.424936521168   0.573548797559   0.388848851949
0.490037392971   0.510831451119   0.358228287967

6. CH* and H* on Co(111)

C Co H
1.0
7.386800000000 -12.794312905350   0.000000000000
7.386800000000 12.794312905350   0.000000000000
0.000000000000   0.000000000000 21.031300000000
C Co H
1 144   2
Selective dynamics
Direct
0.440200111796   0.548991252828   0.335987784763
0.277650931554   0.055285120025   0.187933023661
0.165818623455   0.165977409531   0.280067486320
0.277579794251   0.221903518089   0.188046347612

0.166558771745	0.999645923547	0.279970185795
0.666763398195	0.000261335160	0.279857825810
0.444325011067	0.221929533559	0.187959510729
0.999517477612	0.166108758774	0.280118074577
0.444274307999	0.055374445197	0.187860486397
0.610969275297	0.055453932681	0.187934214424
0.499956657469	0.165879585620	0.280164779266
0.610952126046	0.222134507938	0.187931774378
0.499933617773	0.000286939241	0.280009864189
0.333165162005	0.999865172086	0.280089756157
0.833548208884	0.666339652556	0.279898978032
0.333282173367	0.165868333760	0.280024002954
0.610891765024	0.388982678049	0.188011539423
0.111012293303	0.055365700279	0.187876010184
0.777631926905	0.055406890774	0.187921671814
0.944392856706	0.222080530739	0.187931864975
0.833465645321	0.000376460196	0.279987636000
0.110942384302	0.555510573941	0.187965300124
0.999288615292	0.332846779025	0.279932202933
0.666546450389	0.166733682661	0.280096248078
0.111278864605	0.388926095814	0.187979534048
0.777651039050	0.222130958799	0.188057043749
0.278543482056	0.388903663964	0.188099873909
0.278540713343	0.556538231168	0.187850045380
0.165513105659	0.332368333804	0.280060542273
0.444527587522	0.555714794455	0.187220623011
0.331522579191	0.329943023983	0.279861872878
0.999232658821	0.499713801407	0.280106569790
0.443516523885	0.388731834563	0.188069200895
0.165860109260	0.499631588851	0.280006177301
0.502157357142	0.495368402706	0.283004994141
0.331340496507	0.668046138275	0.279776945183
0.444034576879	0.721197076159	0.188317108992
0.666616523982	0.332783956584	0.279922847012
0.777633593770	0.388838327461	0.187961137215
0.667085958188	0.499837772269	0.280017027012
0.944363151231	0.388799420773	0.188009671673
0.833525088798	0.499811355678	0.280053443021
0.944325304392	0.555484236583	0.187893429419
0.777685576733	0.555518336032	0.187954705004
0.833207345235	0.333218071905	0.280114756994
0.000040049875	0.666432918771	0.280138733255
0.110881680529	0.722151703142	0.187859545623
0.000253768662	0.833295822637	0.279916714456
0.277649539043	0.722205813729	0.188040875022
0.000100424241	0.000056520076	0.280024138003

0.110947482170	0.222016387511	0.187970610962
0.111054221451	0.888816094545	0.187824928395
0.499902794820	0.332241786813	0.280026917669
0.611078223348	0.556674863051	0.188122011647
0.166367986033	0.833242317348	0.280009417481
0.333157039718	0.833580418688	0.279986668054
0.610780042121	0.721527830784	0.188260432580
0.499188295255	0.833986159398	0.279939790377
0.610743739348	0.888435473248	0.187898072730
0.444433313110	0.888660100630	0.188039472422
0.500628520955	0.668705089681	0.285311722235
0.670450378490	0.667835403414	0.280998192444
0.777614295790	0.722243672906	0.188038137534
0.668084452632	0.836235027384	0.280923369418
0.944450114011	0.722099547392	0.187930553551
0.835320435087	0.834513504028	0.279830141461
0.944301959006	0.888773463242	0.187888004668
0.777503242779	0.888661273551	0.187958232824
0.165728046211	0.666494638833	0.279976630754
0.277650488031	0.888828539687	0.187913749462
0.833189598553	0.166622986313	0.280014088289
0.944326534248	0.055359826137	0.187878614012
0.326543109610	0.496485687926	0.282943734443
0.499999868709	0.333333245837	0.000000000000
0.055555540978	0.277777704852	0.095592368850
0.166666622895	-0.000000000000	0.000000000000
0.055555540986	0.111111081972	0.095592368850
0.333333245790	-0.000000000000	0.000000000000
0.666666491640	0.166666622880	0.000000000000
0.555555409738	0.111111081972	0.095592368850
0.166666622887	0.166666622880	0.000000000000
0.555555409731	0.277777704852	0.095592368850
0.388888786768	0.277777704852	0.095592368850
0.499999868752	-0.000000000000	0.000000000000
0.388888786776	0.111111081972	0.095592368850
0.666666491647	-0.000000000000	0.000000000000
0.333333245782	0.166666622880	0.000000000000
0.499999868701	0.499999868717	0.000000000000
0.499999868745	0.166666622880	0.000000000000
0.388888786792	0.611110950689	0.095592368850
0.222222163873	0.277777704852	0.095592368850
0.888888655520	0.277777704852	0.095592368850
0.722222032633	0.111111081972	0.095592368850
0.000000000024	0.333333245837	0.000000000000
0.222222163866	0.444444327731	0.095592368850
0.000000000016	0.499999868717	0.000000000000

0.833333114535	0.166666622880	0.000000000000
0.222222163897	0.611110950689	0.095592368850
0.888888655528	0.111111081972	0.095592368850
0.055555541002	0.611110950689	0.095592368850
0.055555540971	0.444444327731	0.095592368850
0.333333245814	0.333333245837	0.000000000000
0.555555409655	0.444444327731	0.095592368850
0.333333245806	0.499999868717	0.000000000000
0.166666622911	0.499999868717	0.000000000000
0.555555409687	0.611110950689	0.095592368850
0.166666622919	0.333333245837	0.000000000000
0.000000000000	0.000000000000	0.000000000000
0.333333245830	0.833333114555	0.000000000000
0.555555409711	0.944444196526	0.095592368850
0.666666491663	0.499999868717	0.000000000000
0.888888655544	0.611110950689	0.095592368850
0.833333114558	0.499999868717	0.000000000000
0.722222032649	0.611110950689	0.095592368850
0.833333114566	0.333333245837	0.000000000000
0.722222032618	0.444444327731	0.095592368850
0.888888655513	0.444444327731	0.095592368850
0.000000000048	0.666666491675	0.000000000000
0.999999999972	0.833333114555	0.000000000000
0.222222163921	0.944444196526	0.095592368850
0.166666622935	0.833333114555	0.000000000000
0.055555540958	0.944444196526	0.095592368850
0.999999999992	0.166666622880	0.000000000000
0.222222163881	0.111111081972	0.095592368850
0.222222163889	0.777777573569	0.095592368850
0.666666491671	0.333333245837	0.000000000000
0.388888786760	0.444444327731	0.095592368850
0.166666622943	0.666666491675	0.000000000000
0.499999868725	0.833333114555	0.000000000000
0.388888786816	0.944444196526	0.095592368850
0.499999868732	0.666666491675	0.000000000000
0.388888786784	0.777777573569	0.095592368850
0.555555409679	0.777777573569	0.095592368850
0.666666491627	0.666666491675	0.000000000000
0.666666491620	0.833333114555	0.000000000000
0.888888655568	0.944444196526	0.095592368850
0.833333114582	0.833333114555	0.000000000000
0.722222032606	0.944444196526	0.095592368850
0.833333114590	0.666666491675	0.000000000000
0.722222032574	0.777777573569	0.095592368850
0.888888655537	0.777777573569	0.095592368850
0.333333245837	0.666666491675	0.000000000000

0.055555540994 0.777777573569 0.095592368850
0.833333114542 -0.000000000000 0.000000000000
0.722222032626 0.277777704852 0.095592368850
0.440734807046 0.546440355858 0.388350503237
0.615447670204 0.728392088889 0.326482867048

7. CH* on Co(111)

C Co H
1.0
7.386800000000 -12.794312905350 0.000000000000
7.386800000000 12.794312905350 0.000000000000
0.000000000000 0.000000000000 21.031300000000
C Co H
1 144 1
Selective dynamics
Direct
0.444284110247 0.555356589088 0.334918327379
0.999945530616 0.999938021830 0.280074003303
0.111005443477 0.055412323596 0.187899760957
0.999424387189 0.166100821799 0.280078341280
0.277734413983 0.055414397584 0.187897885393
0.165979103292 0.166035794633 0.280011596244
0.277657379479 0.221991327716 0.187993462094
0.111026933371 0.222102025188 0.187992756218
0.166500016348 0.999678555502 0.279949476998
0.333313208937 0.999944789044 0.280073340070
0.444394957810 0.055481333591 0.187878146102
0.333376291084 0.166041376836 0.280012490735
0.611056433076 0.055461379188 0.187934957817
0.499991418632 0.166102559079 0.280074147088
0.611050534676 0.222181589618 0.187931537579
0.444398583123 0.222106845135 0.187991587517
0.331612909060 0.329904648661 0.279877492026
0.499997747427 0.000334532867 0.280077372527
0.666778299641 0.000234885040 0.279905364743
0.944409738490 0.055479881849 0.187879716123
0.833273494815 0.166606391181 0.280054787922
0.944461910433 0.222182705823 0.187932177049
0.833656515057 0.000325958718 0.280074334480
0.111041662527 0.555507734707 0.187991379739
0.777731604819 0.055462378234 0.187933799069
0.777749691972 0.222173368867 0.188031327762
-0.000422530764 0.333062224854 0.279901016055
0.278641567895 0.388957727728 0.188099063922
0.166069380930 0.499725712403 0.280102282236
0.278630854736 0.556259010885 0.188099059921

0.166079534864	0.332857796910	0.280100193095
0.444403956478	0.555485272888	0.187052105947
0.111353811792	0.388962955374	0.187982272040
0.999488002790	0.499896325095	0.280082246504
0.722222032574	0.777777573569	0.095592368850
0.443641033568	0.388960084107	0.188104459157
0.610932951297	0.388964035377	0.187984444818
0.610944453895	0.556256150541	0.188103034124
0.500092473666	0.332870440066	0.280098444996
0.777783861136	0.555507350489	0.187991838193
0.666796711000	0.333061334685	0.279902890510
0.777737811464	0.388877562547	0.187932449523
0.666958580196	0.499728479983	0.280106833548
0.502867849592	0.496963534527	0.283105464228
0.944462986380	0.388873563392	0.187933556572
0.944435817123	0.555543075266	0.187882468397
0.833284668130	0.333248404004	0.280055888254
0.111084294547	0.888841253828	0.187835617009
0.999941054432	0.666572099522	0.280072410483
0.111011579259	0.722188164287	0.187899040385
0.000144305563	0.833357007930	0.279943377042
0.833708070244	0.499891561944	0.280078338549
0.277661753412	0.722250761421	0.187989459326
0.166538248749	0.833359790253	0.279944883657
0.277745949814	0.888913087073	0.187897351096
0.444416067435	0.888868243904	0.187987817748
0.331591411027	0.668222433267	0.279871501354
0.443651817672	0.721270931235	0.188104573316
0.333385463903	0.833853713134	0.280000900216
0.610943892524	0.721277185041	0.188103755884
0.500071332476	0.833676020720	0.280101604951
0.165989765105	0.666492948656	0.280003088837
0.610952984377	0.888581940142	0.187988645564
0.777777749931	0.888865427384	0.187986480348
0.669960774985	0.668230004230	0.279873428452
0.777908951957	0.722248776268	0.187987647673
0.666924791168	0.833669428640	0.280101921315
0.944497439794	0.722188149853	0.187898050678
0.833790123383	0.833845730312	0.280002638541
0.502815618613	0.672325237288	0.283101497735
0.666656939643	0.166613887459	0.280053874075
0.944494868107	0.888913753299	0.187895151836
0.833817186820	0.666494616948	0.280004666211
0.000000000000	0.000000000000	0.000000000000
0.222222163873	0.277777704852	0.095592368850
0.166666622887	0.166666622880	0.000000000000

0.055555540978	0.277777704852	0.095592368850
0.166666622895	-0.000000000000	0.000000000000
0.055555540986	0.111111081972	0.095592368850
0.888888655528	0.111111081972	0.095592368850
0.999999999992	0.166666622880	0.000000000000
0.333333245790	-0.000000000000	0.000000000000
0.555555409731	0.277777704852	0.095592368850
0.499999868745	0.166666622880	0.000000000000
0.388888786768	0.277777704852	0.095592368850
0.499999868752	-0.000000000000	0.000000000000
0.388888786776	0.111111081972	0.095592368850
0.555555409738	0.111111081972	0.095592368850
0.333333245782	0.166666622880	0.000000000000
0.666666491647	-0.000000000000	0.000000000000
0.666666491640	0.166666622880	0.000000000000
0.333333245806	0.499999868717	0.000000000000
0.833333114535	0.166666622880	0.000000000000
0.722222032626	0.277777704852	0.095592368850
0.833333114542	-0.000000000000	0.000000000000
0.722222032633	0.111111081972	0.095592368850
0.000000000024	0.333333245837	0.000000000000
0.222222163866	0.444444327731	0.095592368850
0.888888655520	0.277777704852	0.095592368850
0.000000000016	0.499999868717	0.000000000000
0.166666622911	0.499999868717	0.000000000000
0.055555541002	0.611110950689	0.095592368850
0.166666622919	0.333333245837	0.000000000000
0.055555540971	0.444444327731	0.095592368850
0.333333245814	0.333333245837	0.000000000000
0.555555409655	0.444444327731	0.095592368850
0.222222163897	0.611110950689	0.095592368850
0.555555409687	0.611110950689	0.095592368850
0.327368165181	0.496938792219	0.283096358168
0.499999868701	0.499999868717	0.000000000000
0.555555409679	0.777777573569	0.095592368850
0.666666491671	0.333333245837	0.000000000000
0.888888655513	0.444444327731	0.095592368850
0.666666491663	0.499999868717	0.000000000000
0.888888655544	0.611110950689	0.095592368850
0.833333114558	0.499999868717	0.000000000000
0.722222032649	0.611110950689	0.095592368850
0.388888786760	0.444444327731	0.095592368850
0.833333114566	0.333333245837	0.000000000000
0.000000000048	0.666666491675	0.000000000000
0.222222163889	0.777777573569	0.095592368850
0.999999999972	0.833333114555	0.000000000000

```
0.222222163921   0.944444196526   0.095592368850
0.166666622935   0.833333114555   0.000000000000
0.222222163881   0.111111081972   0.095592368850
0.722222032618   0.444444327731   0.095592368850
0.499999868709   0.333333245837   0.000000000000
0.055555540958   0.944444196526   0.095592368850
0.166666622943   0.666666491675   0.000000000000
0.333333245830   0.833333114555   0.000000000000
0.555555409711   0.944444196526   0.095592368850
0.499999868725   0.833333114555   0.000000000000
0.388888786816   0.944444196526   0.095592368850
0.499999868732   0.666666491675   0.000000000000
0.333333245837   0.666666491675   0.000000000000
0.388888786784   0.777777573569   0.095592368850
0.888888655537   0.777777573569   0.095592368850
0.666666491620   0.833333114555   0.000000000000
0.888888655568   0.944444196526   0.095592368850
0.833333114582   0.833333114555   0.000000000000
0.722222032606   0.944444196526   0.095592368850
0.833333114590   0.666666491675   0.000000000000
0.666666491627   0.666666491675   0.000000000000
0.055555540994   0.777777573569   0.095592368850
0.388888786792   0.611110950689   0.095592368850
0.444199902938   0.555198750214   0.387371775094
```

8. C* and H* on Co(111)

```
C Co H
1.0
  7.386800000000 -12.794312905350   0.000000000000
  7.386800000000  12.794312905350   0.000000000000
  0.000000000000   0.000000000000  21.031300000000
C Co H
  1 144   1
Selective dynamics
Direct
  0.440132224108   0.548686357034   0.324067300917
  0.999849570240   0.999905845883   0.280063636582
  0.110896462329   0.055166817847   0.187876558841
  0.999159717358   0.165729363123   0.280196963533
  0.277604899712   0.055139340623   0.187907608553
  0.165480251365   0.165589758935   0.280085444839
  0.277563520672   0.221877244467   0.188023701163
  0.110825906994   0.221831275411   0.187876017547
  0.166473733579   0.999427405239   0.279989552648
  0.332937372110   0.999608908048   0.280085059377
  0.444176926055   0.055195470640   0.187877109600
```

0.333273328874	0.165475119268	0.280050970789
0.610922689912	0.055376743336	0.187951563817
0.499942567744	0.165451788472	0.280239175453
0.610924682923	0.222061940090	0.187964556513
0.444264626760	0.221735841538	0.187871085906
0.330821896890	0.328446807065	0.279888601921
0.499915710662	0.000330623391	0.280103993553
0.666942958410	0.000741503917	0.279981890245
0.944315164665	0.055222905086	0.187894829274
0.833041272527	0.166467960978	0.280003276892
0.944322307415	0.221990711080	0.187947910815
0.833790253917	0.000685038996	0.280066448097
0.110810462075	0.555577885090	0.187862703536
0.777616984661	0.055323166787	0.187929151911
0.777619338313	0.222076485567	0.188024341076
0.001381541920	0.332424077877	0.280032969163
0.278181266211	0.388667436741	0.187977285246
0.163347856247	0.499844952148	0.280338480656
0.278325444152	0.556211476680	0.187955442504
0.163469415231	0.330169273908	0.280254663232
0.444413014930	0.555477306824	0.187893873035
0.111209471557	0.388928550956	0.188096406021
0.998866671654	0.499618229310	0.280212919481
0.722222032574	0.777777573569	0.095592368850
0.443697036461	0.388589951865	0.187977337362
0.610924703330	0.388979805602	0.188146729739
0.610964388782	0.556419365630	0.188201822440
0.499749664662	0.330036453718	0.280273469086
0.777852489708	0.555584194194	0.187885245847
0.666847134777	0.332380676331	0.280072683951
0.777614181357	0.388773975199	0.187972879554
0.669640095115	0.499975423542	0.280318634292
0.504477550578	0.493282642445	0.282260629855
0.944297189209	0.388748856461	0.188010135479
0.944321270407	0.555540655567	0.187900119557
0.833114441607	0.333068828708	0.280100456924
0.111064973128	0.888809397614	0.187779642519
0.999885994560	0.666573263437	0.280134828374
0.110787543584	0.722208840918	0.187839892826
0.000507856573	0.833283990634	0.280002401439
0.833693695143	0.499600980331	0.280146141136
0.277627821017	0.722169161706	0.187998275570
0.166229189107	0.833296236547	0.280044336634
0.277599177801	0.888819117694	0.187888283097
0.444379422152	0.888554869872	0.187990256336
0.330513595596	0.668775263298	0.279794392131

0.444229082085	0.721151050014	0.188533465336
0.333153359206	0.833889083070	0.280033269299
0.610693214832	0.721578416092	0.188254236001
0.499298980065	0.835478798787	0.280251563461
0.165394800198	0.666450748336	0.280008231950
0.610684840456	0.888398793244	0.187999384945
0.777699965624	0.888640164994	0.187807941072
0.671964584171	0.668198817669	0.281048374699
0.777753643830	0.722329556674	0.187989122545
0.670127406449	0.838362177445	0.281111019008
0.944617738944	0.722162190809	0.187897277203
0.835632845319	0.834762247635	0.279887499874
0.503092939748	0.672538706458	0.285152009923
0.666535362807	0.166610742346	0.280089956594
0.944418690059	0.888754956948	0.187924049853
0.834016840207	0.666412135200	0.279949101854
0.000000000000	0.000000000000	0.000000000000
0.222222163873	0.277777704852	0.095592368850
0.166666622887	0.166666622880	0.000000000000
0.055555540978	0.277777704852	0.095592368850
0.166666622895	-0.000000000000	0.000000000000
0.055555540986	0.111111081972	0.095592368850
0.888888655528	0.111111081972	0.095592368850
0.999999999992	0.166666622880	0.000000000000
0.333333245790	-0.000000000000	0.000000000000
0.555555409731	0.277777704852	0.095592368850
0.499999868745	0.166666622880	0.000000000000
0.388888786768	0.277777704852	0.095592368850
0.499999868752	-0.000000000000	0.000000000000
0.388888786776	0.111111081972	0.095592368850
0.555555409738	0.111111081972	0.095592368850
0.333333245782	0.166666622880	0.000000000000
0.666666491647	-0.000000000000	0.000000000000
0.666666491640	0.166666622880	0.000000000000
0.333333245806	0.499999868717	0.000000000000
0.833333114535	0.166666622880	0.000000000000
0.722222032626	0.277777704852	0.095592368850
0.833333114542	-0.000000000000	0.000000000000
0.722222032633	0.111111081972	0.095592368850
0.000000000024	0.333333245837	0.000000000000
0.222222163866	0.444444327731	0.095592368850
0.888888655520	0.277777704852	0.095592368850
0.000000000016	0.499999868717	0.000000000000
0.166666622911	0.499999868717	0.000000000000
0.055555541002	0.611110950689	0.095592368850
0.166666622919	0.333333245837	0.000000000000

0.055555540971	0.444444327731	0.095592368850
0.333333245814	0.333333245837	0.000000000000
0.555555409655	0.444444327731	0.095592368850
0.222222163897	0.611110950689	0.095592368850
0.555555409687	0.611110950689	0.095592368850
0.321702244127	0.493960651986	0.281924402891
0.499999868701	0.499999868717	0.000000000000
0.555555409679	0.777777573569	0.095592368850
0.666666491671	0.333333245837	0.000000000000
0.888888655513	0.444444327731	0.095592368850
0.666666491663	0.499999868717	0.000000000000
0.888888655544	0.611110950689	0.095592368850
0.833333114558	0.499999868717	0.000000000000
0.722222032649	0.611110950689	0.095592368850
0.388888786760	0.444444327731	0.095592368850
0.833333114566	0.333333245837	0.000000000000
0.000000000048	0.666666491675	0.000000000000
0.222222163889	0.777777573569	0.095592368850
0.999999999972	0.833333114555	0.000000000000
0.222222163921	0.944444196526	0.095592368850
0.166666622935	0.833333114555	0.000000000000
0.222222163881	0.111111081972	0.095592368850
0.722222032618	0.444444327731	0.095592368850
0.499999868709	0.333333245837	0.000000000000
0.055555540958	0.944444196526	0.095592368850
0.166666622943	0.666666491675	0.000000000000
0.333333245830	0.833333114555	0.000000000000
0.555555409711	0.944444196526	0.095592368850
0.499999868725	0.833333114555	0.000000000000
0.388888786816	0.944444196526	0.095592368850
0.499999868732	0.666666491675	0.000000000000
0.333333245837	0.666666491675	0.000000000000
0.388888786784	0.777777573569	0.095592368850
0.888888655537	0.777777573569	0.095592368850
0.666666491620	0.833333114555	0.000000000000
0.888888655568	0.944444196526	0.095592368850
0.833333114582	0.833333114555	0.000000000000
0.722222032606	0.944444196526	0.095592368850
0.833333114590	0.666666491675	0.000000000000
0.666666491627	0.666666491675	0.000000000000
0.055555540994	0.777777573569	0.095592368850
0.388888786792	0.611110950689	0.095592368850
0.619344072039	0.731231451541	0.325906043368

9. CO_2 on Co(111)

C Co O

```
   1.00000000000000
     7.3868000000000000  -12.7943129053500009    0.0000000000000000
     7.3868000000000000   12.7943129053500009    0.0000000000000000
     0.0000000000000000    0.0000000000000000   21.0313000000000017
   C    Co   O
     1   144     2
Selective dynamics
Direct
  0.5651851689660751  0.5674381831500688  0.3749957247923962
  0.7222220326260000  0.2777777048519994  0.0955923688499993
  0.8333331145420004  0.0000000000000000  0.0000000000000000
  0.7222220326329989  0.1111110819719983  0.0955923688499993
  0.0000000000240021  0.3333332458369966  0.0000000000000000
  0.2222221638660002  0.4444443277309986  0.0955923688499993
  0.0000000000160014  0.4999998687169978  0.0000000000000000
  0.8333331145350016  0.1666666228800011  0.0000000000000000
  0.2222221638970012  0.6111109506890031  0.0955923688499993
  0.0555555410020006  0.6111109506890031  0.0955923688499993
  0.1666666229190028  0.3333332458369966  0.0000000000000000
  0.0555555409709996  0.4444443277309986  0.0955923688499993
  0.3333332458140035  0.3333332458369966  0.0000000000000000
  0.5555554096549997  0.4444443277309986  0.0955923688499993
  0.3333332458060028  0.4999998687169978  0.0000000000000000
  0.1666666229110021  0.4999998687169978  0.0000000000000000
  0.5555554096870026  0.6111109506890031  0.0955923688499993
  0.8888886555199988  0.2777777048519994  0.0955923688499993
  0.8888886555279996  0.1111110819719983  0.0955923688499993
  0.2222221638729991  0.2777777048519994  0.0955923688499993
  0.1666666228870000  0.1666666228800011  0.0000000000000000
  0.0555555409779984  0.2777777048519994  0.0955923688499993
  0.1666666228950007  0.0000000000000000  0.0000000000000000
  0.0555555409859991  0.1111110819719983  0.0955923688499993
  0.3333332457900013  0.0000000000000000  0.0000000000000000
  0.6666664916400009  0.1666666228800011  0.0000000000000000
  0.5555554097379982  0.1111110819719983  0.0955923688499993
  0.5555554097309994  0.2777777048519994  0.0955923688499993
  0.4999998687450002  0.1666666228800011  0.0000000000000000
  0.3888887867679998  0.2777777048519994  0.0955923688499993
  0.4999998687519991  0.0000000000000000  0.0000000000000000
  0.3888887867760005  0.1111110819719983  0.0955923688499993
  0.6666664916469998  0.0000000000000000  0.0000000000000000
  0.3333332457820006  0.1666666228800011  0.0000000000000000
  0.4999998687010034  0.4999998687169978  0.0000000000000000
  0.3888887867920019  0.6111109506890031  0.0955923688499993
  0.4999998687089970  0.3333332458369966  0.0000000000000000
  0.3333332458299978  0.8333331145550034  0.0000000000000000
```

0.5555554097109976	0.9444441965259998	0.0955923688499993
0.4999998687249985	0.8333331145550034	0.0000000000000000
0.3888887868159969	0.9444441965259998	0.0955923688499993
0.4999998687319973	0.6666664916750022	0.0000000000000000
0.3888887867840012	0.7777775735689971	0.0955923688499993
0.5555554096790019	0.7777775735689971	0.0955923688499993
0.6666664916269980	0.6666664916750022	0.0000000000000000
0.6666664916199991	0.8333331145550034	0.0000000000000000
0.8888886555680031	0.9444441965259998	0.0955923688499993
0.8333331145819969	0.8333331145550034	0.0000000000000000
0.7222220326059983	0.9444441965259998	0.0955923688499993
0.8333331145899976	0.6666664916750022	0.0000000000000000
0.7222220325740025	0.7777775735689971	0.0955923688499993
0.8888886555370021	0.7777775735689971	0.0955923688499993
0.3333332458369966	0.6666664916750022	0.0000000000000000
0.0555555409939998	0.7777775735689971	0.0955923688499993
0.1666666229429978	0.6666664916750022	0.0000000000000000
0.3888887867599990	0.4444443277309986	0.0955923688499993
0.6666664916710019	0.3333332458369966	0.0000000000000000
0.8888886555130000	0.4444443277309986	0.0955923688499993
0.6666664916630012	0.4999998687169978	0.0000000000000000
0.8888886555440010	0.6111109506890031	0.0955923688499993
0.8333331145580019	0.4999998687169978	0.0000000000000000
0.7222220326490003	0.6111109506890031	0.0955923688499993
0.8333331145660026	0.3333332458369966	0.0000000000000000
0.7222220326179993	0.4444443277309986	0.0955923688499993
0.0000000000479972	0.6666664916750022	0.0000000000000000
0.2222221638890005	0.7777775735689971	0.0955923688499993
0.9999999999719975	0.8333331145550034	0.0000000000000000
0.2222221639210034	0.9444441965259998	0.0955923688499993
0.1666666229349971	0.8333331145550034	0.0000000000000000
0.0555555409579966	0.9444441965259998	0.0955923688499993
0.9999999999919993	0.1666666228800011	0.0000000000000000
0.2222221638809998	0.1111110819719983	0.0955923688499993
0.0000000000000000	0.0000000000000000	0.0000000000000000
0.3305231661562703	0.4993975137112155	0.2856763376523497
0.9435941012555423	0.0554176021645021	0.1931805494212369
0.8321352219548052	0.1665184374839642	0.2865661009622730
0.9434252676416143	0.2219829142300463	0.1933155721840593
0.8328500207442189	0.0007313178975308	0.2863038864912436
0.1101743427848578	0.5552281750705321	0.1932234545714961
0.9987012901867692	0.3330136236125536	0.2864704919548772
0.6652138682116048	0.1658079213082228	0.2863433967445271
0.1100907192460369	0.3885308864208352	0.1932015158598031
0.2771680839885413	0.3886641235891686	0.1928133533983428
0.1650474904555813	0.4996230954911118	0.2862488864995977

0.2770038158740901	0.5549549643684604	0.1930713077628357
0.1650525227547266	0.3325828017870822	0.2859721023875965
0.4447297253530487	0.5547338905086583	0.1944615733100285
0.3311374949748568	0.3315081725363059	0.2855866916194289
0.9988062676812209	0.4996136073703989	0.2863501611941885
0.4445444189367327	0.3897339467324446	0.1940829950348399
0.7767617067086069	0.0553295375870492	0.1932736791452616
0.7766306749300397	0.2217918134807479	0.1933444265230329
0.1102323754177262	0.0552316977825917	0.1931285977046949
0.9988029108379216	0.1665660847239304	0.2863920951070163
0.2767958192576623	0.0551665563522454	0.1932017830427444
0.1650734264934512	0.1658274070198989	0.2861733699014578
0.2770715385247237	0.2221256797030271	0.1931171901287654
0.1655200195325864	0.9995146444166698	0.2863313227784853
0.6654806343782991	0.0002152473488942	0.2863337419753851
0.4435378590018407	0.2219379622945978	0.1931382150741985
0.4432005677811313	0.0549309515559856	0.1931610668979527
0.3318323255364735	0.1655515480047038	0.2862148353634774
0.6098769324779214	0.0549397960541202	0.1932031300519400
0.4985941224745966	0.1653780075976961	0.2862124663743312
0.6099833404201660	0.2216163122799289	0.1931022583763484
0.4984382131550646	-0.0006458034481994	0.2862489175792172
0.3318585552379177	0.9991611743201176	0.2863521355989006
0.8341931395408299	0.6663108144761942	0.2857828738161884
0.6103721820548735	0.3887917047856272	0.1928450020401100
0.4974834866778561	0.4987241019755230	0.2932850535707126
0.3318176457622546	0.6663960356015206	0.2861192284878383
0.4437282881742399	0.7214538398684162	0.1929434225424682
0.3318663709436361	0.8328309492409057	0.2863205599037451
0.6112673348874317	0.7216595518078184	0.1939281266741116
0.4988262138814074	0.8335206260489811	0.2860792748830055
0.6101927528109001	0.8883722355058139	0.1931134125887805
0.4433550419190770	0.8882618597041704	0.1931565854302681
0.4977346051568355	0.6678130394158559	0.2854367348163602
0.6687338681871562	0.6691933404333991	0.2899413919342829
0.7760603810443838	0.7215283313844535	0.1938612643721924
0.6664166250563791	0.8352180255471391	0.2857700684776989
0.9435229636325956	0.7220004739915662	0.1931882639004869
0.8341335916693042	0.8350329745490679	0.2858006361275526
0.9435562512791764	0.8885464175685699	0.1930959247639767
0.7770869366603411	0.8888157592417080	0.1929887922411437
0.1654126300734187	0.6663095462641878	0.2862901432778474
0.2767142858875899	0.8883073904109383	0.1932247469380551
0.1654135706963195	0.8328937356914512	0.2864155855621566
0.6095253333585686	0.5559147555974868	0.1958035210924661
0.4990180096768295	0.3311523839522317	0.2857800531201781

0.7768432770935471 0.5554438908544240 0.1928235696198480
0.6656737825453862 0.3317920791678607 0.2860046898434144
0.7768183875009613 0.3884346208510832 0.1931016471705045
0.6671094221832257 0.4974671918713895 0.2855928296551535
0.9433997261671552 0.3885621868604029 0.1933280815658804
0.8327275006932151 0.4991730798709684 0.2859452241059744
0.9435424848164070 0.5552612269540731 0.1931452851800352
0.8320859204578508 0.3327516189181302 0.2865232464069031
0.1101609680627522 0.8885515690367737 0.1932331147648552
-0.0005504709234404 0.6663101549953411 0.2863442539274731
0.1101506226622073 0.7218711799274034 0.1931972172745556
-0.0006084899837401 0.8333995127242195 0.2863283158593457
0.2768528639238914 0.7217105855507692 0.1931759087896834
-0.0005279426855409 0.0003185433446394 0.2862613637544785
0.1101731261038332 0.2219063352893345 0.1931347845678827
0.4878806305861687 0.4920035323414246 0.3992385978989860
0.6471943404560219 0.6477681738546051 0.3908967670479968

10. CO* and O* on Co(111)

C Co O
1.00000000000000
7.3868000000000000 -12.7943129053500009 0.0000000000000000
7.3868000000000000 12.7943129053500009 0.0000000000000000
0.0000000000000000 0.0000000000000000 21.0313000000000017
C Co O
1 144 2
Selective dynamics
Direct
0.3764951276690937 0.4382068218021755 0.3557437157912471
0.7222220326260000 0.2777777048519994 0.0955923688499993
0.8333331145420004 0.0000000000000000 0.0000000000000000
0.7222220326329989 0.1111110819719983 0.0955923688499993
0.0000000000240021 0.3333332458369966 0.0000000000000000
0.2222221638660002 0.4444443277309986 0.0955923688499993
0.0000000000160014 0.4999998687169978 0.0000000000000000
0.8333331145350016 0.1666666228800011 0.0000000000000000
0.2222221638970012 0.6111109506890031 0.0955923688499993
0.0555555410020006 0.6111109506890031 0.0955923688499993
0.1666666229190028 0.3333332458369966 0.0000000000000000
0.0555555409709996 0.4444443277309986 0.0955923688499993
0.3333332458140035 0.3333332458369966 0.0000000000000000
0.5555554096549997 0.4444443277309986 0.0955923688499993
0.3333332458060028 0.4999998687169978 0.0000000000000000
0.1666666229110021 0.4999998687169978 0.0000000000000000
0.5555554096870026 0.6111109506890031 0.0955923688499993
0.8888886555199988 0.2777777048519994 0.0955923688499993

0.8888886555279996	0.1111110819719983	0.0955923688499993
0.2222221638729991	0.2777777048519994	0.0955923688499993
0.1666666228870000	0.1666666228800011	0.0000000000000000
0.0555555409779984	0.2777777048519994	0.0955923688499993
0.1666666228950007	0.0000000000000000	0.0000000000000000
0.0555555409859991	0.1111110819719983	0.0955923688499993
0.3333332457900013	0.0000000000000000	0.0000000000000000
0.6666664916400009	0.1666666228800011	0.0000000000000000
0.5555554097379982	0.1111110819719983	0.0955923688499993
0.5555554097309994	0.2777777048519994	0.0955923688499993
0.4999998687450002	0.1666666228800011	0.0000000000000000
0.3888887867679998	0.2777777048519994	0.0955923688499993
0.4999998687519991	0.0000000000000000	0.0000000000000000
0.3888887867760005	0.1111110819719983	0.0955923688499993
0.6666664916469998	0.0000000000000000	0.0000000000000000
0.3333332457820006	0.1666666228800011	0.0000000000000000
0.4999998687010034	0.4999998687169978	0.0000000000000000
0.3888887867920019	0.6111109506890031	0.0955923688499993
0.4999998687089970	0.3333332458369966	0.0000000000000000
0.3333332458299978	0.8333331145550034	0.0000000000000000
0.5555554097109976	0.9444441965259998	0.0955923688499993
0.4999998687249985	0.8333331145550034	0.0000000000000000
0.3888887868159969	0.9444441965259998	0.0955923688499993
0.4999998687319973	0.6666664916750022	0.0000000000000000
0.3888887867840012	0.7777775735689971	0.0955923688499993
0.5555554096790019	0.7777775735689971	0.0955923688499993
0.6666664916269980	0.6666664916750022	0.0000000000000000
0.6666664916199991	0.8333331145550034	0.0000000000000000
0.8888886555680031	0.9444441965259998	0.0955923688499993
0.8333331145819969	0.8333331145550034	0.0000000000000000
0.7222220326059983	0.9444441965259998	0.0955923688499993
0.8333331145899976	0.6666664916750022	0.0000000000000000
0.7222220325740025	0.7777775735689971	0.0955923688499993
0.8888886555370021	0.7777775735689971	0.0955923688499993
0.3333332458369966	0.6666664916750022	0.0000000000000000
0.0555555409939998	0.7777775735689971	0.0955923688499993
0.1666666229429978	0.6666664916750022	0.0000000000000000
0.3888887867599990	0.4444443277309986	0.0955923688499993
0.6666664916710019	0.3333332458369966	0.0000000000000000
0.8888886555130000	0.4444443277309986	0.0955923688499993
0.6666664916630012	0.4999998687169978	0.0000000000000000
0.8888886555440010	0.6111109506890031	0.0955923688499993
0.8333331145580019	0.4999998687169978	0.0000000000000000
0.7222220326490003	0.6111109506890031	0.0955923688499993
0.8333331145660026	0.3333332458369966	0.0000000000000000
0.7222220326179993	0.4444443277309986	0.0955923688499993

0.0000000000479972	0.6666664916750022	0.0000000000000000
0.2222221638890005	0.7777775735689971	0.0955923688499993
0.9999999999719975	0.8333331145550034	0.0000000000000000
0.2222221639210034	0.9444441965259998	0.0955923688499993
0.1666666229349971	0.8333331145550034	0.0000000000000000
0.0555555409579966	0.9444441965259998	0.0955923688499993
0.9999999999919993	0.1666666228800011	0.0000000000000000
0.2222221638809998	0.1111110819719983	0.0955923688499993
0.0000000000000000	0.0000000000000000	0.0000000000000000
0.3325040397027214	0.5004799832931356	0.2886179147896837
0.9444414461676316	0.0555476960275280	0.1932008348677141
0.8334994926205104	0.1667985551327730	0.2864496315474256
0.9443664535047834	0.2222123537547154	0.1932616291217573
0.8335119154878395	0.0003118097806220	0.2863841181328349
0.1110326479269144	0.5555587194685422	0.1931294327385214
0.9998811057451902	0.3333794401953202	0.2862626119291232
0.6667334693305941	0.1665038294316481	0.2864080904917690
0.1109629771463118	0.3888808251374600	0.1929327675815348
0.2780821743100215	0.3890745982415830	0.1936471431392798
0.1665296293895651	0.5000523511730801	0.2860995091667640
0.2778547438685674	0.5557059742326316	0.1928912890065392
0.1652836890465378	0.3327076382963480	0.2859418771941523
0.4449526068902603	0.5551635520351189	0.1944405718397146
0.3325125284606804	0.3322546561560297	0.2886996544741022
0.0002082997352113	0.5000108654599881	0.2863385734655153
0.4449425877747980	0.3898744385536765	0.1944591379532125
0.7778039097795016	0.0555495552240574	0.1932863236116869
0.7777911502976126	0.2223036894733711	0.1932775787935008
0.1110510821449938	0.0555770813358539	0.1931432390674317
0.9999012227990626	0.1666091935444050	0.2862713796423156
0.2777751158592007	0.0554952680464851	0.1930555315549959
0.1665698527910648	0.1666338003538446	0.2861291291661425
0.2778757741922927	0.2222419151705488	0.1928926024416860
0.1667993743703219	0.0001128120796520	0.2862600739678328
0.6667482081873918	0.0003453782042081	0.2864149997438310
0.4444750284420759	0.2221492235998496	0.1929313152824600
0.4443212876258607	0.0555232857650986	0.1930945022767048
0.3326446875168371	0.1661744403130603	0.2860247855011288
0.6110823076382782	0.0555843738175429	0.1932548393983635
0.5000079986245098	0.1657653713607968	0.2861972933565781
0.6110627430391572	0.2222503646925672	0.1931983024341820
0.4999279598858020	0.0000042727005641	0.2862533435963946
0.3332888247976094	0.9999549095162041	0.2863079771688176
0.8371115621512258	0.6686167072118069	0.2859500626201661
0.6112981332876690	0.3893567835408052	0.1935063965616763
0.4966185984302069	0.4983905962828771	0.2941621704002270

0.3326255381442050 0.6666111579465963 0.2860092516980745
0.4444534694341171 0.7223957108117330 0.1929242770693678
0.3332809885637910 0.8334185870758359 0.2863096757124122
0.6112975222173224 0.7220315469272994 0.1935036054149280
0.5000151132606859 0.8343426603485253 0.2861909690941055
0.6110582182140958 0.8889034257846582 0.1931973057133778
0.4443185799784608 0.8888873030034287 0.1930918165119385
0.4988580929939456 0.6699149765888850 0.2856945030930120
0.6684526286641621 0.6705117831043488 0.2896433069088713
0.7771843529756770 0.7219147099979700 0.1936275743193888
0.6672658838319999 0.8345704093718020 0.2861407644467674
0.9445035462660600 0.7222956137499007 0.1932608887877192
0.8339874038977698 0.8342778641175569 0.2860753771041748
0.9445025238186823 0.8887778219565378 0.1932155981450921
0.7777436785690415 0.8888377185214711 0.1930867971242167
0.1667696048395483 0.6667609067588480 0.2862579138952029
0.2776873836987117 0.8888855845661280 0.1932980253876504
0.1666906891769212 0.8333867338570081 0.2864821751048292
0.6110292865022109 0.5555678229562520 0.1938378054779771
0.4988436503683721 0.3290653276347074 0.2857046044492842
0.7771887861327690 0.5553636103327346 0.1936275869614738
0.6672516138414241 0.3328105919301925 0.2861422845879137
0.7777299509191457 0.3889839955650815 0.1930845598304054
0.6684535358214518 0.4980603459112804 0.2896196224943841
0.9444240838409673 0.3889634830248251 0.1931841673083031
0.8339573838804871 0.4998121992972607 0.2860737026300182
0.9444789997260348 0.5557909993002502 0.1932090732291504
0.8334908072115110 0.3332901033095189 0.2863685959797830
0.1111433275005814 0.8888384828909481 0.1932109676889315
1.0007941743298785 0.6667140885101930 0.2863972522330487
0.1111178868536267 0.7223701705659218 0.1932005655375162
0.0008090247180599 0.8341753904713566 0.2863939071762496
0.2777549563030354 0.7223579327467505 0.1930519699120699
0.0002548875114108 0.0003267124186940 0.2863537419412694
0.1109734661799256 0.2221671181255084 0.1929507272702947
0.3722943662840155 0.4366349614072553 0.4124225741630591
0.6172044513328665 0.5586560626210922 0.3455556177770048

11. C* and CO_2 on Co(111)

C Co O
1.0
7.386800000000 -12.794312905350 0.000000000000
7.386800000000 12.794312905350 0.000000000000
0.000000000000 0.000000000000 21.031300000000
C Co O

```
  2 144   2
Selective dynamics
Direct
  0.443407244889   0.556028325220   0.323449613160
  0.688567378064   0.549844110802   0.365625508986
  0.722222032626   0.277777704852   0.095592368850
  0.833333114542  -0.000000000000   0.000000000000
  0.722222032633   0.111111081972   0.095592368850
  0.000000000024   0.333333245837   0.000000000000
  0.222222163866   0.444444327731   0.095592368850
  0.000000000016   0.499999868717   0.000000000000
  0.222222163897   0.611110950689   0.095592368850
  0.166666622911   0.499999868717   0.000000000000
  0.055555541002   0.611110950689   0.095592368850
  0.166666622919   0.333333245837   0.000000000000
  0.055555540971   0.444444327731   0.095592368850
  0.333333245814   0.333333245837   0.000000000000
  0.555555409655   0.444444327731   0.095592368850
  0.333333245806   0.499999868717   0.000000000000
  0.833333114535   0.166666622880   0.000000000000
  0.888888655520   0.277777704852   0.095592368850
  0.666666491640   0.166666622880   0.000000000000
  0.888888655528   0.111111081972   0.095592368850
  0.999999999992   0.166666622880   0.000000000000
  0.222222163873   0.277777704852   0.095592368850
  0.166666622887   0.166666622880   0.000000000000
  0.055555540978   0.277777704852   0.095592368850
  0.166666622895   0.000000000000   0.000000000000
  0.055555540986   0.111111081972   0.095592368850
  0.333333245790   0.000000000000   0.000000000000
  0.555555409738   0.111111081972   0.095592368850
  0.333333245782   0.166666622880   0.000000000000
  0.555555409731   0.277777704852   0.095592368850
  0.499999868745   0.166666622880   0.000000000000
  0.388888786768   0.277777704852   0.095592368850
  0.499999868752   0.000000000000   0.000000000000
  0.388888786776   0.111111081972   0.095592368850
  0.666666491647   0.000000000000   0.000000000000
  0.555555409687   0.611110950689   0.095592368850
  0.499999868701   0.499999868717   0.000000000000
  0.388888786792   0.611110950689   0.095592368850
  0.499999868709   0.333333245837   0.000000000000
  0.333333245830   0.833333114555   0.000000000000
  0.555555409711   0.944444196526   0.095592368850
  0.499999868725   0.833333114555   0.000000000000
  0.388888786816   0.944444196526   0.095592368850
```

0.499999868732	0.666666491675	0.000000000000
0.388888786784	0.777777573569	0.095592368850
0.555555409679	0.777777573569	0.095592368850
0.666666491627	0.666666491675	0.000000000000
0.666666491620	0.833333114555	0.000000000000
0.888888655568	0.944444196526	0.095592368850
0.833333114582	0.833333114555	0.000000000000
0.722222032606	0.944444196526	0.095592368850
0.833333114590	0.666666491675	0.000000000000
0.722222032574	0.777777573569	0.095592368850
0.888888655537	0.777777573569	0.095592368850
0.333333245837	0.666666491675	0.000000000000
0.055555540994	0.777777573569	0.095592368850
0.166666622943	0.666666491675	0.000000000000
0.388888786760	0.444444327731	0.095592368850
0.666666491671	0.333333245837	0.000000000000
0.888888655513	0.444444327731	0.095592368850
0.666666491663	0.499999868717	0.000000000000
0.888888655544	0.611110950689	0.095592368850
0.833333114558	0.499999868717	0.000000000000
0.722222032649	0.611110950689	0.095592368850
0.833333114566	0.333333245837	0.000000000000
0.722222032618	0.444444327731	0.095592368850
0.000000000048	0.666666491675	0.000000000000
0.222222163889	0.777777573569	0.095592368850
0.999999999972	0.833333114555	0.000000000000
0.222222163921	0.944444196526	0.095592368850
0.166666622935	0.833333114555	0.000000000000
0.055555540958	0.944444196526	0.095592368850
0.222222163881	0.111111081972	0.095592368850
0.000000000000	0.000000000000	0.000000000000
0.835721445214	0.669719712088	0.283656469690
0.944938030727	0.889263062462	0.187631868869
0.777769008892	0.055875310251	0.187903779623
0.666988738097	0.166676629297	0.280062391037
0.944588857981	0.055752047373	0.187808880977
0.833322590917	0.167331437548	0.280099270874
0.944514163605	0.222380655380	0.187983678279
0.834396597322	0.002127461986	0.280025688881
0.667063143916	0.001299727283	0.279967674731
0.111119331182	0.555802878353	0.187833502362
0.111384951643	0.389062592096	0.188109812478
-0.000162529472	0.500397218273	0.280125017505
0.278540538886	0.389070744793	0.187974440954
0.164034074291	0.500038854890	0.280313925210
0.278446042395	0.556162461178	0.188050383056

0.164229574318	0.330937802845	0.280306663324
-0.000798949011	0.333082481146	0.279964007664
0.444804489640	0.555458370684	0.188025443329
0.777734242702	0.222273186666	0.187971920410
0.610875343771	0.222038817935	0.187985516564
0.111136060186	0.222103000546	0.187882774468
0.001304020543	0.001580606444	0.279942505288
0.111307285954	0.055619565432	0.187767969501
-0.000359597058	0.166757821118	0.280096109745
0.277710525542	0.055251557810	0.187857316872
0.166537917916	0.166517926276	0.279999962750
0.500084943011	0.000911374721	0.280083457348
0.277884994430	0.222103966339	0.187900575844
0.444311141980	0.221926042041	0.187943816763
0.333182075717	-0.000122419010	0.280057719578
0.444183912244	0.055309008808	0.187777943436
0.333064431835	0.165667043186	0.280047279677
0.611011563886	0.055548298417	0.187915912581
0.499670338845	0.165227733420	0.280016548627
0.166715114007	-0.000233040802	0.279928204069
0.331159094120	0.328677346307	0.279619555087
0.444378112146	0.389259975330	0.188053431553
0.166746424015	0.833858633633	0.279874523084
0.277563022209	0.888998218112	0.187749020117
0.165809863234	0.666770554401	0.279940839565
0.444334371869	0.889006433862	0.187750249097
0.330917831375	0.669276118114	0.279855441435
0.444106346935	0.721494415023	0.188075055985
0.333368738208	0.834305865636	0.279942624347
0.323046325006	0.495115814376	0.282188606034
0.611560749080	0.720919751951	0.189020386455
0.610836293736	0.888606105550	0.187951259309
0.504808285440	0.676650613849	0.282075811333
0.777877931175	0.889143927368	0.187671634737
0.673341986418	0.673657254654	0.282775156608
0.777943178713	0.721847192277	0.187995542771
0.668878574707	0.837730750163	0.279913739958
0.499803397467	0.836020198283	0.280196525398
0.943270886826	0.721796866834	0.188983545185
0.277718163834	0.722394016817	0.187864999198
0.110311352606	0.722090096186	0.187984667464
0.610112938140	0.387496705350	0.186797556759
0.503442042037	0.495138906301	0.282388894648
0.611521027873	0.556213706485	0.188560532609
0.500044279436	0.330585248101	0.279881333842
0.777513803800	0.555829789575	0.188514409586

```
 0.666783712721   0.332375983350   0.279535659857
 0.002349637884   0.835599696761   0.279572200304
 0.777515404980   0.389163396192   0.187939219344
 0.944547873613   0.389115327379   0.187856828388
 0.835082768830   0.498637302618   0.280180591057
 0.943912540183   0.555537216713   0.188037600115
 0.833443459110   0.333435631784   0.279859811739
 0.111086432962   0.888731175683   0.187562478369
-0.000161772116   0.666901836386   0.279844506394
 0.666537987047   0.494618613375   0.282536296258
 0.837190979079   0.840157376248   0.278961334213
 0.662434375556   0.508717811916   0.417166163093
 0.746842689804   0.657746031263   0.356123164475
```

12. 2CO* on Co(111)

```
C Co O
1.0
  7.386800000000 -12.794312905350   0.000000000000
  7.386800000000  12.794312905350   0.000000000000
  0.000000000000   0.000000000000  21.031300000000
C Co O
  2 144   2
Selective dynamics
Direct
  0.438936596681   0.552617229703   0.345107048628
  0.704730410160   0.666893592430   0.362979995819
  0.722222032626   0.277777704852   0.095592368850
  0.833333114542  -0.000000000000   0.000000000000
  0.722222032633   0.111111081972   0.095592368850
  0.000000000024   0.333333245837   0.000000000000
  0.222222163866   0.444444327731   0.095592368850
  0.000000000016   0.499999868717   0.000000000000
  0.222222163897   0.611110950689   0.095592368850
  0.166666622911   0.499999868717   0.000000000000
  0.055555541002   0.611110950689   0.095592368850
  0.166666622919   0.333333245837   0.000000000000
  0.055555540971   0.444444327731   0.095592368850
  0.333333245814   0.333333245837   0.000000000000
  0.555555409655   0.444444327731   0.095592368850
  0.333333245806   0.499999868717   0.000000000000
  0.833333114535   0.166666622880   0.000000000000
  0.888888655520   0.277777704852   0.095592368850
  0.666666491640   0.166666622880   0.000000000000
  0.888888655528   0.111111081972   0.095592368850
  0.999999999992   0.166666622880   0.000000000000
  0.222222163873   0.277777704852   0.095592368850
```

0.166666622887	0.166666622880	0.000000000000
0.055555540978	0.277777704852	0.095592368850
0.166666622895	0.000000000000	0.000000000000
0.055555540986	0.111111081972	0.095592368850
0.333333245790	0.000000000000	0.000000000000
0.555555409738	0.111111081972	0.095592368850
0.333333245782	0.166666622880	0.000000000000
0.555555409731	0.277777704852	0.095592368850
0.499999868745	0.166666622880	0.000000000000
0.388888786768	0.277777704852	0.095592368850
0.499999868752	0.000000000000	0.000000000000
0.388888786776	0.111111081972	0.095592368850
0.666666491647	0.000000000000	0.000000000000
0.555555409687	0.611110950689	0.095592368850
0.499999868701	0.499999868717	0.000000000000
0.388888786792	0.611110950689	0.095592368850
0.499999868709	0.333333245837	0.000000000000
0.333333245830	0.833333114555	0.000000000000
0.555555409711	0.944444196526	0.095592368850
0.499999868725	0.833333114555	0.000000000000
0.388888786816	0.944444196526	0.095592368850
0.499999868732	0.666666491675	0.000000000000
0.388888786784	0.777777573569	0.095592368850
0.555555409679	0.777777573569	0.095592368850
0.666666491627	0.666666491675	0.000000000000
0.666666491620	0.833333114555	0.000000000000
0.888888655568	0.944444196526	0.095592368850
0.833333114582	0.833333114555	0.000000000000
0.722222032606	0.944444196526	0.095592368850
0.833333114590	0.666666491675	0.000000000000
0.722222032574	0.777777573569	0.095592368850
0.888888655537	0.777777573569	0.095592368850
0.333333245837	0.666666491675	0.000000000000
0.055555540994	0.777777573569	0.095592368850
0.166666622943	0.666666491675	0.000000000000
0.388888786760	0.444444327731	0.095592368850
0.666666491671	0.333333245837	0.000000000000
0.888888655513	0.444444327731	0.095592368850
0.666666491663	0.499999868717	0.000000000000
0.888888655544	0.611110950689	0.095592368850
0.833333114558	0.499999868717	0.000000000000
0.722222032649	0.611110950689	0.095592368850
0.833333114566	0.333333245837	0.000000000000
0.722222032618	0.444444327731	0.095592368850
0.000000000048	0.666666491675	0.000000000000
0.222222163889	0.777777573569	0.095592368850

0.999999999972	0.833333114555	0.000000000000
0.222222163921	0.944444196526	0.095592368850
0.166666622935	0.833333114555	0.000000000000
0.055555540958	0.944444196526	0.095592368850
0.222222163881	0.111111081972	0.095592368850
0.000000000000	0.000000000000	0.000000000000
0.835884236444	0.667001202837	0.280273975969
0.944172244416	0.888690013171	0.188038175864
0.777686268955	0.055476736404	0.187919896918
0.666809594750	0.166596919118	0.280153986477
0.944375410093	0.055468736241	0.187833144214
0.833213294989	0.166710378800	0.280077818572
0.944400113395	0.222114916345	0.187941749928
0.833709147435	0.000570977411	0.279984104635
0.666500818494	0.000083963746	0.279856941307
0.111139009798	0.555599339083	0.187990972215
0.111313946448	0.388973030607	0.187930157042
-0.000298052581	0.500034061850	0.280008848320
0.278193373241	0.388910420217	0.187776630550
0.166031267039	0.499437056486	0.279952135835
0.278221691594	0.555895350871	0.187777718490
0.166016995518	0.333264273287	0.279943438849
-0.000106928517	0.333252820249	0.279891478962
0.444528868688	0.555587263024	0.188051011132
0.777786749348	0.222186810382	0.188064418675
0.611015480268	0.221968234666	0.187955288776
0.111116232306	0.222146718751	0.187995935225
-0.000021759684	0.000239925319	0.279978631331
0.111141903497	0.055545000832	0.187931249846
-0.000339757839	0.166320342181	0.280016810366
0.277787230060	0.055395223304	0.187882695467
0.166468575815	0.166435263242	0.280007445300
0.499854240757	0.000313216608	0.280017691702
0.277673579861	0.222059897732	0.187987215987
0.444332156548	0.222084504380	0.187945538183
0.333464956833	0.000011121301	0.280160830619
0.444307923205	0.055438511468	0.187891627631
0.333130491984	0.166142454748	0.279978836890
0.610992916727	0.055606822690	0.187921925034
0.499863421941	0.166099339062	0.280003860666
0.166640900268	-0.000154528461	0.279956503640
0.331403825560	0.329825292403	0.279622574401
0.444280988201	0.389366190809	0.188054635474
0.166638039205	0.833450636908	0.279950042840
0.277813537084	0.889006252688	0.187897858134
0.166556143766	0.666627400274	0.280034916896

0.444395265595 0.888779443669 0.187956504718
0.331386322327 0.668157091404 0.279671173540
0.444356279351 0.721474216267 0.188098845462
0.333155128429 0.833545665146 0.279974396390
0.330341765970 0.498472191011 0.282398579606
0.611108295500 0.720859969129 0.188634954603
0.611202566829 0.888341051583 0.188054065304
0.499817509235 0.667917520510 0.282588082856
0.777422636748 0.888216814506 0.187983342928
0.670396704977 0.668553497328 0.284338107500
0.778249264250 0.722338468499 0.187727510498
0.665895337030 0.834699501942 0.279775199977
0.500049238780 0.833473885365 0.279884164298
0.944137512517 0.722101285058 0.188050731334
0.277703857995 0.722214084528 0.187972138988
0.111168380719 0.722220066425 0.187955595018
0.611158033289 0.389409941989 0.188039405860
0.499453482582 0.498255595618 0.282414465877
0.610852638697 0.556096779457 0.188142949958
0.499868160948 0.332842272743 0.279863430363
0.777474374610 0.555847570226 0.188059141850
0.666559125412 0.333200144913 0.279742983812
0.001413230041 0.834109903151 0.279922596193
0.777714915843 0.388853322479 0.187884001761
0.944403199818 0.388927712426 0.187917072063
0.833928604867 0.499607604357 0.280000326024
0.944380681388 0.555554126832 0.187821992409
0.833201296241 0.333155056965 0.280044542843
0.111238002504 0.888905573960 0.187834526130
-0.000211115047 0.666337271884 0.279937059257
0.665658909425 0.497165678862 0.279834110867
0.835583358686 0.835698900714 0.279880567555
0.443358346863 0.554316206287 0.402107924076
0.721110275345 0.665289920085 0.417271656085

13. C-C-C and CO_2 on Co(111)

C Co O
1.00000000000000
7.3868000000000000 -12.7943129053500009 0.0000000000000000
7.3868000000000000 12.7943129053500009 0.0000000000000000
0.0000000000000000 0.0000000000000000 21.0313000000000017
C Co O
3 144 1
Selective dynamics
Direct
0.4407748916650850 0.5609576329577262 0.3379674159363682

0.2660426552212614	0.5593739791390259	0.3374079930428499
0.3689266249602807	0.5919957305825018	0.3511654632131419
0.7222220326260000	0.2777777048519994	0.0955923688499993
0.8333331145420004	0.0000000000000000	0.0000000000000000
0.7222220326329989	0.1111110819719983	0.0955923688499993
0.0000000000240021	0.3333332458369966	0.0000000000000000
0.2222221638660002	0.4444443277309986	0.0955923688499993
0.0000000000160014	0.4999998687169978	0.0000000000000000
0.2222221638970012	0.6111109506890031	0.0955923688499993
0.1666666229190028	0.3333332458369966	0.0000000000000000
0.0555555410020006	0.6111109506890031	0.0955923688499993
0.8333331145350016	0.1666666228800011	0.0000000000000000
0.0555555409709996	0.4444443277309986	0.0955923688499993
0.3333332458140035	0.3333332458369966	0.0000000000000000
0.5555554096549997	0.4444443277309986	0.0955923688499993
0.3333332458060028	0.4999998687169978	0.0000000000000000
0.1666666229110021	0.4999998687169978	0.0000000000000000
0.8888886555199988	0.2777777048519994	0.0955923688499993
0.3888887867760005	0.1111110819719983	0.0955923688499993
0.8888886555279996	0.1111110819719983	0.0955923688499993
0.9999999999919992	0.1666666228800011	0.0000000000000000
0.2222221638729991	0.2777777048519994	0.0955923688499993
0.1666666228870000	0.1666666228800011	0.0000000000000000
0.0555555409779984	0.2777777048519994	0.0955923688499993
0.1666666228950007	0.0000000000000000	0.0000000000000000
0.0555555409859991	0.1111110819719983	0.0955923688499993
0.3333332457900013	0.0000000000000000	0.0000000000000000
0.5555554097379982	0.1111110819719983	0.0955923688499993
0.3333332457820006	0.1666666228800011	0.0000000000000000
0.5555554097309994	0.2777777048519994	0.0955923688499993
0.4999998687450002	0.1666666228800011	0.0000000000000000
0.3888887867679998	0.2777777048519994	0.0955923688499993
0.4999998687519991	0.0000000000000000	0.0000000000000000
0.5555554096870026	0.6111109506890031	0.0955923688499993
0.6666664916469998	0.0000000000000000	0.0000000000000000
0.6666664916400009	0.1666666228800011	0.0000000000000000
0.4999998687010034	0.4999998687169978	0.0000000000000000
0.3888887867599990	0.4444443277309986	0.0955923688499993
0.4999998687089970	0.3333332458369966	0.0000000000000000
0.3333332458299978	0.8333331145550034	0.0000000000000000
0.5555554097109976	0.9444441965259998	0.0955923688499993
0.4999998687249985	0.8333331145550034	0.0000000000000000
0.3888887868159969	0.9444441965259998	0.0955923688499993
0.4999998687319973	0.6666664916750022	0.0000000000000000
0.3888887867840012	0.7777775735689971	0.0955923688499993
0.5555554096790019	0.7777775735689971	0.0955923688499993

0.6666664916269980	0.6666664916750022	0.0000000000000000
0.6666664916199991	0.8333331145550034	0.0000000000000000
0.8888886555680031	0.9444441965259998	0.0955923688499993
0.8333331145819969	0.8333331145550034	0.0000000000000000
0.7222220326059983	0.9444441965259998	0.0955923688499993
0.8333331145899976	0.6666664916750022	0.0000000000000000
0.7222220325740025	0.7777775735689971	0.0955923688499993
0.8888886555370021	0.7777775735689971	0.0955923688499993
0.3333332458369966	0.6666664916750022	0.0000000000000000
0.0555555409939998	0.7777775735689971	0.0955923688499993
0.1666666229429978	0.6666664916750022	0.0000000000000000
0.2222221638809998	0.1111110819719983	0.0955923688499993
0.6666664916710019	0.3333332458369966	0.0000000000000000
0.8888886555130000	0.4444443277309986	0.0955923688499993
0.6666664916630012	0.4999998687169978	0.0000000000000000
0.8888886555440010	0.6111109506890031	0.0955923688499993
0.8333331145580019	0.4999998687169978	0.0000000000000000
0.7222220326490003	0.6111109506890031	0.0955923688499993
0.8333331145660026	0.3333332458369966	0.0000000000000000
0.7222220326179993	0.4444443277309986	0.0955923688499993
0.0000000000479972	0.6666664916750022	0.0000000000000000
0.2222221638890005	0.7777775735689971	0.0955923688499993
0.9999999999719976	0.8333331145550034	0.0000000000000000
0.2222221639210034	0.9444441965259998	0.0955923688499993
0.1666666229349971	0.8333331145550034	0.0000000000000000
0.0555555409579966	0.9444441965259998	0.0955923688499993
0.3888887867920019	0.6111109506890031	0.0955923688499993
0.0000000000000000	0.0000000000000000	0.0000000000000000
0.8356111001973185	0.8356856626772800	0.2797191286609140
0.9448554113457344	0.8891463533585164	0.1877042063267524
0.6669962882038513	0.1662320868177111	0.2800209091133506
0.9445176662357276	0.0552675077126819	0.1875495030755802
0.8333886125592885	0.1665917614618039	0.2801231243463123
0.9445705946203022	0.2222743539302431	0.1880668899338908
0.8341899356945222	0.0006437911243656	0.2799394937960387
0.1114033208782228	0.5559432894251041	0.1882938330594734
0.7780574108959061	0.0555115166153634	0.1877588960407098
0.9975048855324564	0.3314779745101368	0.2800811123041936
0.9980395472507472	0.5001002332941563	0.2802140625428087
0.2776411767970074	0.3877355872503627	0.1876099642664478
0.1567116498871063	0.4957011667372935	0.2844016088131651
0.2776144923491350	0.5559808383298870	0.1869650909198440
0.1618086067998015	0.3271782836871366	0.2792860095638685
0.4448025309534032	0.5559810529845115	0.1881376911546433
0.1118003359239397	0.3896167880868540	0.1885105976334308
0.6681883009396014	0.0019687858716094	0.2800291983768564

0.7778726894640193	0.2220986828465664	0.1880156817281048
0.5008591969897296	0.0023081686785811	0.2799468732359850
0.1107943685753933	0.2218920658547745	0.1875193501814821
0.0010797050397747	0.0007943238430242	0.2799223466831913
0.1112212101426781	0.0552089530131420	0.1875329629919631
0.9985554497152848	0.1649602841849319	0.2798613874216934
0.2778698346137830	0.0556445191055392	0.1875928103337335
0.1661190780743184	0.1655983714365587	0.2796767136525584
0.2781264612637320	0.2223739289921036	0.1878117396035485
0.1669845702661504	0.0002862759967949	0.2799167864566667
0.4446190713517285	0.2224319822351086	0.1877714470437762
0.3334846813544163	0.0007459227580298	0.2798664406883924
0.4443444710252465	0.0555224725758591	0.1875341469824847
0.3335911956591812	0.1659342263612669	0.2799108014006286
0.6113208772096648	0.0555481112624197	0.1877987686519452
0.5000495165777475	0.1662129683408106	0.2798343473441380
0.6112270826453147	0.2221133489177235	0.1880182169339778
0.3315853726569787	0.3287842222171914	0.2797335928961701
0.8400902555781039	0.6699954379741129	0.2790823163645104
0.4456533726485380	0.3911649699489664	0.1898332367836216
0.6105165950843791	0.3891583445781733	0.1881033911073531
0.1650326218659192	0.6687646915097951	0.2798875005164467
0.4443808399916181	0.8891681107528651	0.1876539939627459
0.3365216303599854	0.6785597442330643	0.2840380553318166
0.4438715307027423	0.7215488895864279	0.1877249453265737
0.3337466081885975	0.8368850062711335	0.2798198217881898
0.6103544047947446	0.7206702307063668	0.1891048890229265
0.2778741308340479	0.8890440325594978	0.1875570680752723
0.5028576691946909	0.8393339983287325	0.2794648556009506
0.5046125414528461	0.6780780360259757	0.2839472854167301
0.7778916889325213	0.8886805585002825	0.1876458301692016
0.6738974667303133	0.6734947014331516	0.2843311119346252
0.7778189507001536	0.7217125801136968	0.1882995875632231
0.6702113338103961	0.8370892536456399	0.2800435598858130
0.9451009682247398	0.7228757809430403	0.1874715280137099
0.6114821447144783	0.8888551791790292	0.1879023479610036
0.3246005854175949	0.4896684368486681	0.2804861541843439
0.1673759895797548	0.8347713510471050	0.2797565796280863
0.0014657305528581	0.8347260511266812	0.2797029164737933
0.5033799940321099	0.5007501997343894	0.2946411607897049
0.6108767825862274	0.5557140790270471	0.1873337861868689
0.4983911542251078	0.3284448390409106	0.2795603297098315
0.7777232114660040	0.5560581883675317	0.1881980470937439
0.6674809146892107	0.3323875884256395	0.2798359548995828
0.7778086262000784	0.3888861983643470	0.1876803846002115
0.2773957136736794	0.7218427553236859	0.1881471529752428

0.6723493254673840 0.4956641839497460 0.2832392349742608
0.8347753491835499 0.4989328199811077 0.2798088669637517
0.9446852294360004 0.5557433429138683 0.1877034648883173
0.8327535852297390 0.3324399369185055 0.2799102763802919
0.1117963402757644 0.8895354841021436 0.1876131160280449
0.0001895872041692 0.6667017063198710 0.2795819832673516
0.1116505005153157 0.7223195749230394 0.1878437215517824
0.9447123543279397 0.3891188805310077 0.1879408781398499
0.6311631113319909 0.5615848240538556 0.3360125626019204

14. C-C and 2CO on Co(111)

```
C    Co   O
   1.00000000000000
   7.3868000000000000 -12.7943129053500009   0.0000000000000000
   7.3868000000000000  12.7943129053500009   0.0000000000000000
   0.0000000000000000   0.0000000000000000  21.0313000000000017
C    Co   O
3  144  1
Selective dynamics
Direct
  0.5778518182751865  0.6200322507782210  0.3507686322478111
  0.2740819157217224  0.5547489478135008  0.3361991600498025
  0.3812025410022963  0.6100992741936401  0.3382590234698694
  0.7222220326260000  0.2777777048519994  0.0955923688499993
  0.8333331145420004  0.0000000000000000  0.0000000000000000
  0.7222220326329989  0.1111110819719983  0.0955923688499993
  0.0000000000240021  0.3333332458369966  0.0000000000000000
  0.2222221638660002  0.4444443277309986  0.0955923688499993
  0.0000000000160014  0.4999998687169978  0.0000000000000000
  0.2222221638970012  0.6111109506890031  0.0955923688499993
  0.1666666229190028  0.3333332458369966  0.0000000000000000
  0.0555555410020006  0.6111109506890031  0.0955923688499993
  0.8333331145350016  0.1666666228800011  0.0000000000000000
  0.0555555409709996  0.4444443277309986  0.0955923688499993
  0.3333332458140035  0.3333332458369966  0.0000000000000000
  0.5555554096549997  0.4444443277309986  0.0955923688499993
  0.3333332458060028  0.4999998687169978  0.0000000000000000
  0.1666666229110021  0.4999998687169978  0.0000000000000000
  0.8888886555199988  0.2777777048519994  0.0955923688499993
  0.3888887867760005  0.1111110819719983  0.0955923688499993
  0.8888886555279996  0.1111110819719983  0.0955923688499993
  0.9999999999919992  0.1666666228800011  0.0000000000000000
  0.2222221638729991  0.2777777048519994  0.0955923688499993
  0.1666666228870000  0.1666666228800011  0.0000000000000000
  0.0555555409779984  0.2777777048519994  0.0955923688499993
  0.1666666228950007  0.0000000000000000  0.0000000000000000
```

0.0555555409859991	0.1111110819719983	0.0955923688499993
0.3333332457900013	0.0000000000000000	0.0000000000000000
0.5555554097379982	0.1111110819719983	0.0955923688499993
0.3333332457820006	0.1666666228800011	0.0000000000000000
0.5555554097309994	0.2777777048519994	0.0955923688499993
0.4999998687450002	0.1666666228800011	0.0000000000000000
0.3888887867679998	0.2777777048519994	0.0955923688499993
0.4999998687519991	0.0000000000000000	0.0000000000000000
0.5555554096870026	0.6111109506890031	0.0955923688499993
0.6666664916469998	0.0000000000000000	0.0000000000000000
0.6666664916400009	0.1666666228800011	0.0000000000000000
0.4999998687010034	0.4999998687169978	0.0000000000000000
0.3888887867599990	0.4444443277309986	0.0955923688499993
0.4999998687089970	0.3333332458369966	0.0000000000000000
0.3333332458299978	0.8333331145550034	0.0000000000000000
0.5555554097109976	0.9444441965259998	0.0955923688499993
0.4999998687249985	0.8333331145550034	0.0000000000000000
0.3888887868159969	0.9444441965259998	0.0955923688499993
0.4999998687319973	0.6666664916750022	0.0000000000000000
0.3888887867840012	0.7777775735689971	0.0955923688499993
0.5555554096790019	0.7777775735689971	0.0955923688499993
0.6666664916269980	0.6666664916750022	0.0000000000000000
0.6666664916199991	0.8333331145550034	0.0000000000000000
0.8888886555680031	0.9444441965259998	0.0955923688499993
0.8333331145819969	0.8333331145550034	0.0000000000000000
0.7222220326059983	0.9444441965259998	0.0955923688499993
0.8333331145899976	0.6666664916750022	0.0000000000000000
0.7222220325740025	0.7777775735689971	0.0955923688499993
0.8888886555370021	0.7777775735689971	0.0955923688499993
0.3333332458369966	0.6666664916750022	0.0000000000000000
0.0555555409939998	0.7777775735689971	0.0955923688499993
0.1666666229429978	0.6666664916750022	0.0000000000000000
0.2222221638809998	0.1111110819719983	0.0955923688499993
0.6666664916710019	0.3333332458369966	0.0000000000000000
0.8888886555130000	0.4444443277309986	0.0955923688499993
0.6666664916630012	0.4999998687169978	0.0000000000000000
0.8888886555440010	0.6111109506890031	0.0955923688499993
0.8333331145580019	0.4999998687169978	0.0000000000000000
0.7222220326490003	0.6111109506890031	0.0955923688499993
0.8333331145660026	0.3333332458369966	0.0000000000000000
0.7222220326179993	0.4444443277309986	0.0955923688499993
0.0000000000479972	0.6666664916750022	0.0000000000000000
0.2222221638890005	0.7777775735689971	0.0955923688499993
0.9999999999719976	0.8333331145550034	0.0000000000000000
0.2222221639210034	0.9444441965259998	0.0955923688499993
0.1666666229349971	0.8333331145550034	0.0000000000000000

0.0555555409579966	0.9444441965259998	0.0955923688499993
0.3888887867920019	0.6111109506890031	0.0955923688499993
0.0000000000000000	0.0000000000000000	0.0000000000000000
0.8339108051101020	0.8335400969237976	0.2796011359353129
0.9438457394510557	0.8885502254511193	0.1877743670202643
0.6668069400575604	0.1669708377163663	0.2800851652845958
0.9444640792812354	0.0552567330400459	0.1877172551532648
0.8332403470802363	0.1667336371234683	0.2801023184307847
0.9444295204934572	0.2222390731218931	0.1880713625681897
0.8334336062834267	0.9999676480951740	0.2798965051092872
0.1118177562606811	0.5555103195232126	0.1886994700346797
0.7779931593168818	0.0555928349007952	0.1877060619515010
0.9985056071230188	0.3320004577167057	0.2801013308376964
0.9986751542176576	0.4998706158784469	0.2801071983045820
0.2767284327459497	0.3877641801905469	0.1874414625691528
0.1601183091836691	0.4967397223627317	0.2854428501462672
0.2776956327099152	0.5555964334377949	0.1868676576734720
0.1644570075964468	0.3288509348525911	0.2791677108708038
0.4444143192283586	0.5560300726259667	0.1887862057882037
0.1118107940052902	0.3896995202957274	0.1886735483454416
0.6680588837983460	0.0011781219087926	0.2801490663241382
0.7777569861404378	0.2221262240038222	0.1879970704164144
0.5004817606106947	0.0010037272815022	0.2798711873452269
0.1105193640735804	0.2218444495055786	0.1878354921573020
0.0000338644804502	0.9998000882655530	0.2798478366478649
0.1109549771384717	0.0551554717801699	0.1877691381282160
0.9986330233807240	0.1651433664394886	0.2801423254730912
0.2774837100725956	0.0554570801985652	0.1877619151840161
0.1659074787762420	0.1653075704264300	0.2798907917885148
0.2774079457356951	0.2215790967673755	0.1877841837568794
0.1669358712738402	0.0001646861509052	0.2800413659136856
0.4442899361113017	0.2218889715797378	0.1879255122196973
0.3335374055249463	0.0014869323587364	0.2800784399377841
0.4443175507992591	0.0555332483855524	0.1878057054813678
0.3336286686387881	0.1659030933454095	0.2800444319502088
0.6111155196826801	0.0556170317380504	0.1879641978154834
0.5002408992777569	0.1662684544470062	0.2801784041377576
0.6110691409935001	0.2220668868013062	0.1880391976996442
0.3331865575727655	0.3304642940940533	0.2795677435809177
0.8339198411813936	0.6667335408493762	0.2796198741485222
0.4440772880463265	0.3888386963077932	0.1878155094646935
0.6109943684337360	0.3889945053794782	0.1878090402386137
0.1648539721767855	0.6690791629652981	0.2793014694333204
0.4441645559396301	0.8884145040695102	0.1880505231075227
0.3350808673097516	0.6791002189297670	0.2818482047907446
0.4446254466406450	0.7216839897573635	0.1881824853028691

```
  0.3330045902645483  0.8366737390192917  0.2794817588435343
  0.6093840965009174  0.7211346864140391  0.1894254921454534
  0.2774046710208390  0.8892044039847672  0.1876190997187051
  0.5041048187295869  0.8390141873790875  0.2798458619569810
  0.5034448097819035  0.6661372074976289  0.2943115040656268
  0.7770396211098545  0.8880220897266887  0.1879735410163024
  0.6694355191640777  0.6671994843811724  0.2816427975071759
  0.7783243493666543  0.7224397939649019  0.1871330499256139
  0.6678943189799658  0.8366806353503071  0.2800495089645812
  0.9444802028612640  0.7225112719263486  0.1877317966317688
  0.6112624338840883  0.8886056134367903  0.1879459033083350
  0.3333504266811748  0.4900645606562675  0.2825158004565088
  0.1662248531421190  0.8343684988149604  0.2798048469716163
  0.9998742269958421  0.8335121607933781  0.2797491803547398
  0.5039617603133567  0.4967012408867950  0.2817491133803661
  0.6113083190403110  0.5559140160896573  0.1880390659962062
  0.5004256514554962  0.3324711136950544  0.2796713380002841
  0.7778884762042449  0.5558788082218178  0.1877122490348276
  0.6669213979479669  0.3329194594286361  0.2798975129835424
  0.7777795359599772  0.3889045551099535  0.1878564378443292
  0.2764819645144767  0.7223265591906496  0.1870575921016860
  0.6690834412823418  0.4980482841247231  0.2799394429660476
  0.8336419569865723  0.5000113626304311  0.2800573087930113
  0.9445906536109446  0.5555134167212882  0.1880536441577052
  0.8326554324568733  0.3327446782920973  0.2799608594820154
  0.1108763240217385  0.8892181303476738  0.1876860045576153
  0.9989243425409340  0.6666163262642557  0.2800413181029310
  0.1107530126997460  0.7221029802499948  0.1878664561704003
  0.9446350041242124  0.3889960893047562  0.1880231217497927
  0.5768739930854713  0.6040399325694850  0.4064527085203821
```

B. CoGa(111)

1. **CH_4 on CoGa**

```
C Co Ga H
1.0
  6.040950000000 -10.463232325983   0.000000000000
  6.040950000000  10.463232325983   0.000000000000
  0.000000000000   0.000000000000  24.864900000000
C Co Ga H
  1  63  54   4
Selective dynamics
Direct
  0.330540350752   0.322804572914   0.492731451275
  0.444445827215   0.222222913585   0.132246134551
  0.333334370422   0.000000000000   0.198369201847
```

0.666668740844	0.000000000000	0.000000000000
0.888891654475	0.111111456840	0.066123067296
0.777780197636	0.222222913585	0.132246134551
0.666668740844	0.000000000000	0.198369201847
0.000000000002	0.333334370426	0.000000000000
0.222222913633	0.444445827266	0.066123067296
0.111111456795	0.555557284011	0.132246134551
0.000000000002	0.333334370426	0.198369201847
0.333334370424	0.333334370426	0.000000000000
0.555557284055	0.444445827266	0.066123067296
0.444445827216	0.555557284011	0.132246134551
0.333334370424	0.333334370426	0.198369201847
0.666668740846	0.333334370426	0.000000000000
0.888891654477	0.444445827266	0.066123067296
0.777780197638	0.555557284011	0.132246134551
0.666668740846	0.333334370426	0.198369201847
0.000000000004	0.666668740852	0.000000000000
0.222222913635	0.777780197692	0.066123067296
0.111111456797	0.888891654437	0.132246134551
0.000000000004	0.666668740852	0.198369201847
0.333334370426	0.666668740852	0.000000000000
0.555557284057	0.777780197692	0.066123067296
0.444445827218	0.888891654437	0.132246134551
0.333334370426	0.666668740852	0.198369201847
0.888891654479	0.777780197692	0.066123067296
0.777780197640	0.888891654437	0.132246134551
0.666668740848	0.666668740852	0.198369201847
0.555557284053	0.111111456840	0.066123067296
0.333334370422	0.000000000000	0.000000000000
0.666668740848	0.666668740852	0.000000000000
0.111111456793	0.222222913585	0.132246134551
0.000000000000	0.000000000000	0.198369201847
0.222519434639	0.111789510522	0.263219751884
0.109806185732	0.221478461695	0.330664844378
0.555523774483	0.111038298236	0.263309102655
0.444928262215	0.221739701062	0.330733171073
0.332735083069	0.000435301104	0.387486277105
0.888713956897	0.111058922301	0.263283396264
0.777792430282	0.222242439861	0.331862167427
0.666884000499	0.000710085151	0.387654447146
0.222514786033	0.444147316793	0.263237948912
0.111291992885	0.555493055290	0.331947213116
0.001828964625	0.334432421513	0.387430048053
0.554991699349	0.444192375616	0.263257434810
0.445113115137	0.557032714528	0.330750812108
0.000628564879	0.000315428290	0.387527926372

0.888950798979	0.444580209331	0.263273362845
0.332949355635	0.332005447455	0.392791445230
0.000000000000	0.000000000000	0.000000000000
0.222222913631	0.111111456840	0.066123067296
0.777704526579	0.889074864982	0.331887954062
0.889005786744	0.777696493921	0.263236511204
0.332648122241	0.664975587899	0.387507219212
0.665752013280	0.666182847976	0.387532478645
0.555626531630	0.777880567090	0.263288260549
0.000090489655	0.666757403646	0.387714851844
0.111066182942	0.888845604877	0.331966868250
0.222070378978	0.777857569039	0.263248489237
0.665875943525	0.334816629134	0.387409491057
0.444395321543	0.889147655974	0.331814081831
0.777594265073	0.555577146009	0.331910015499
0.555510272373	0.111805020118	0.371689928397
0.333115213316	-0.000362389242	0.295921040972
0.222599467607	0.112335341451	0.370478573024
-0.000388936880	-0.000565047725	0.295943479418
0.666668740848	0.666668740852	0.099184600903
0.888891654479	0.777780197692	0.165307668199
0.777780197640	0.888891654437	0.033061533648
0.444445827218	0.888891654437	0.231430735495
0.555557284057	0.777780197692	0.165307668199
0.333334370426	0.666668740852	0.099184600903
0.777780197640	0.888891654437	0.231430735495
0.666607097249	0.000021123089	0.296074590805
0.888768181640	0.444293547100	0.371657624789
-0.000360914928	0.333525068843	0.295888849057
0.223770503478	0.443423060646	0.370066234533
0.333372390000	0.333391527044	0.298370890415
0.553446300604	0.444339284226	0.370242444179
0.667181355475	0.333510272491	0.295840313692
0.000005841611	0.666538896043	0.296134336886
0.222566186474	0.777668370762	0.371790059621
0.333185073470	0.667276385949	0.295882410301
0.555270236560	0.778201799649	0.371673400202
0.667332072206	0.667204623009	0.295918483581
0.444445827218	0.888891654437	0.033061533648
0.888471775309	0.777642852095	0.371764976317
0.889246570984	0.111546044542	0.371684725738
0.111111456797	0.888891654437	0.231430735495
0.000000000002	0.333334370426	0.099184600903
0.000000000004	0.666668740852	0.099184600903
0.111111456793	0.222222913585	0.033061533648
0.000000000000	0.000000000000	0.099184600903

0.222222913631   0.111111456840   0.165307668199
0.111111456793   0.222222913585   0.231430735495
0.444445827215   0.222222913585   0.033061533648
0.333334370422   0.000000000000   0.099184600903
0.555557284053   0.111111456840   0.165307668199
0.444445827215   0.222222913585   0.231430735495
0.777780197636   0.222222913585   0.033061533648
0.666668740844   0.000000000000   0.099184600903
0.222222913635   0.777780197692   0.165307668199
0.777780197636   0.222222913585   0.231430735495
0.888891654475   0.111111456840   0.165307668199
0.222222913633   0.444445827266   0.165307668199
0.111111456795   0.555557284011   0.231430735495
0.444445827216   0.555557284011   0.033061533648
0.333334370424   0.333334370426   0.099184600903
0.555557284055   0.444445827266   0.165307668199
0.444445827216   0.555557284011   0.231430735495
0.777780197638   0.555557284011   0.033061533648
0.666668740846   0.333334370426   0.099184600903
0.888891654477   0.444445827266   0.165307668199
0.777780197638   0.555557284011   0.231430735495
0.111111456797   0.888891654437   0.033061533648
0.111111456795   0.555557284011   0.033061533648
0.257439889145   0.328469896637   0.467336959449
0.381881831876   0.411799686579   0.515598296114
0.279225766674   0.239496039537   0.518958076729
0.400814628546   0.309359182418   0.469040683888

2. CH_3* and H* on CoGa

C Co Ga H
1.0
6.040950000000 -10.463232325983   0.000000000000
6.040950000000 10.463232325983   0.000000000000
0.000000000000   0.000000000000 24.864900000000
C Co Ga H
1 63 54   4
Selective dynamics
Direct
0.284206105523   0.367431873824   0.461487099199
0.444445827215   0.222222913585   0.132246134551
0.333334370422   0.000000000000   0.198369201847
0.666668740844   0.000000000000   0.000000000000
0.888891654475   0.111111456840   0.066123067296
0.777780197636   0.222222913585   0.132246134551
0.666668740844   0.000000000000   0.198369201847
0.000000000002   0.333334370426   0.000000000000
0.222222913633   0.444445827266   0.066123067296

0.111111456795	0.555557284011	0.132246134551
0.000000000002	0.333334370426	0.198369201847
0.333334370424	0.333334370426	0.000000000000
0.555557284055	0.444445827266	0.066123067296
0.444445827216	0.555557284011	0.132246134551
0.333334370424	0.333334370426	0.198369201847
0.666668740846	0.333334370426	0.000000000000
0.888891654477	0.444445827266	0.066123067296
0.777780197638	0.555557284011	0.132246134551
0.666668740846	0.333334370426	0.198369201847
0.000000000004	0.666668740852	0.000000000000
0.222222913635	0.777780197692	0.066123067296
0.111111456797	0.888891654437	0.132246134551
0.000000000004	0.666668740852	0.198369201847
0.333334370426	0.666668740852	0.000000000000
0.555557284057	0.777780197692	0.066123067296
0.444445827218	0.888891654437	0.132246134551
0.333334370426	0.666668740852	0.198369201847
0.888891654479	0.777780197692	0.066123067296
0.777780197640	0.888891654437	0.132246134551
0.666668740848	0.666668740852	0.198369201847
0.555557284053	0.111111456840	0.066123067296
0.333334370422	0.000000000000	0.000000000000
0.666668740848	0.666668740852	0.000000000000
0.111111456793	0.222222913585	0.132246134551
0.000000000000	0.000000000000	0.198369201847
0.222754892644	0.111600799887	0.263609938045
0.111704734014	0.223659588473	0.330957850576
0.555681589168	0.110982429152	0.263507959276
0.439864963055	0.226764530120	0.332893246927
0.347523571725	0.017436076645	0.388614646742
0.889402388961	0.111807465038	0.263328838557
0.780080218660	0.221754164155	0.330636669974
0.676242206290	-0.009354959894	0.388022534901
0.222229491710	0.444331739679	0.263359806888
0.109848636268	0.556501435572	0.333314513613
-0.002101407773	0.339653243404	0.387829040835
0.555067046420	0.443952585961	0.263658457340
0.442942568335	0.554897307864	0.331056945011
-0.003701925512	-0.003732085705	0.387771464001
0.888081749507	0.444065796854	0.263582523965
0.330400800083	0.334466490420	0.391348879298
0.000000000000	0.000000000000	0.000000000000
0.222222913631	0.111111456840	0.066123067296
0.778824524311	0.887737815813	0.332094869372
0.889072468785	0.777475590058	0.263422925510

0.327544953945	0.668910275154	0.387808306126
0.670289343856	0.670027479985	0.387875580820
0.554889987406	0.777192233239	0.263368246371
0.003364086170	0.662758388359	0.387763418476
0.111167846607	0.889466605916	0.331121482501
0.222494563573	0.778496125672	0.263536904251
0.648781743978	0.319146762202	0.388705300124
0.444709650943	0.886467817111	0.330639761752
0.776793323581	0.555246452813	0.331157496253
0.566480636063	0.100198163293	0.369300941032
0.334224855830	0.000619917169	0.296682627507
0.216993663505	0.109061813286	0.369172708950
-0.000499232346	0.000143741899	0.295956515935
0.666668740848	0.666668740852	0.099184600903
0.888891654479	0.777780197692	0.165307668199
0.777780197640	0.888891654437	0.033061533648
0.444445827218	0.888891654437	0.231430735495
0.555557284057	0.777780197692	0.165307668199
0.333334370426	0.666668740852	0.099184600903
0.777780197640	0.888891654437	0.231430735495
0.667892880139	-0.001397164150	0.295978260109
0.876763057380	0.440174230938	0.372807621351
0.001156627149	0.335474947258	0.296065418892
0.222176450382	0.444838284494	0.370953025127
0.332332107153	0.334375788505	0.296035025169
0.556692918408	0.449325713548	0.369414812559
0.665876979618	0.332373374465	0.296754183371
0.000180378914	0.666185070670	0.296144356431
0.226092522073	0.789352816059	0.372715276959
0.331051961895	0.665324019630	0.296036995691
0.550760033651	0.771900232681	0.371219200363
0.666398206497	0.667050059018	0.296023334436
0.444445827218	0.888891654437	0.033061533648
0.891760595791	0.774738162032	0.371061564906
0.894927159091	0.116321831950	0.371178722644
0.111111456797	0.888891654437	0.231430735495
0.000000000002	0.333334370426	0.099184600903
0.000000000004	0.666668740852	0.099184600903
0.111111456793	0.222222913585	0.033061533648
0.000000000000	0.000000000000	0.099184600903
0.222222913631	0.111111456840	0.165307668199
0.111111456793	0.222222913585	0.231430735495
0.444445827215	0.222222913585	0.033061533648
0.333334370422	0.000000000000	0.099184600903
0.555557284053	0.111111456840	0.165307668199
0.444445827215	0.222222913585	0.231430735495

0.777780197636 0.222222913585 0.033061533648
0.666668740844 0.000000000000 0.099184600903
0.222222913635 0.777780197692 0.165307668199
0.777780197636 0.222222913585 0.231430735495
0.888891654475 0.111111456840 0.165307668199
0.222222913633 0.444445827266 0.165307668199
0.111111456795 0.555557284011 0.231430735495
0.444445827216 0.555557284011 0.033061533648
0.333334370424 0.333334370426 0.099184600903
0.555557284055 0.444445827266 0.165307668199
0.444445827216 0.555557284011 0.231430735495
0.777780197638 0.555557284011 0.033061533648
0.666668740846 0.333334370426 0.099184600903
0.888891654477 0.444445827266 0.165307668199
0.777780197638 0.555557284011 0.231430735495
0.111111456797 0.888891654437 0.033061533648
0.111111456795 0.555557284011 0.033061533648
0.183103742227 0.316033752857 0.473388198811
0.331055592091 0.466507029121 0.476011057230
0.333137175476 0.318050406121 0.478747420638
0.468895869932 0.197372297244 0.396261712237

3. CH_3* on CoGa

C Co Ga H
1.0
6.040950000000 -10.463232325983 0.000000000000
6.040950000000 10.463232325983 0.000000000000
0.000000000000 0.000000000000 24.864900000000
C Co Ga H
1 63 54 3
Selective dynamics
Direct
0.349738658164 0.304470374212 0.467315529102
0.444445827215 0.222222913585 0.132246134551
0.333334370422 0.000000000000 0.198369201847
0.666668740844 0.000000000000 0.000000000000
0.888891654475 0.111111456840 0.066123067296
0.777780197636 0.222222913585 0.132246134551
0.666668740844 0.000000000000 0.198369201847
0.000000000002 0.333334370426 0.000000000000
0.222222913633 0.444445827266 0.066123067296
0.111111456795 0.555557284011 0.132246134551
0.000000000002 0.333334370426 0.198369201847
0.333334370424 0.333334370426 0.000000000000
0.555557284055 0.444445827266 0.066123067296
0.444445827216 0.555557284011 0.132246134551
0.333334370424 0.333334370426 0.198369201847

0.666668740846	0.333334370426	0.000000000000
0.888891654477	0.444445827266	0.066123067296
0.777780197638	0.555557284011	0.132246134551
0.666668740846	0.333334370426	0.198369201847
0.000000000004	0.666668740852	0.000000000000
0.222222913635	0.777780197692	0.066123067296
0.111111456797	0.888891654437	0.132246134551
0.000000000004	0.666668740852	0.198369201847
0.333334370426	0.666668740852	0.000000000000
0.555557284057	0.777780197692	0.066123067296
0.444445827218	0.888891654437	0.132246134551
0.333334370426	0.666668740852	0.198369201847
0.888891654479	0.777780197692	0.066123067296
0.777780197640	0.888891654437	0.132246134551
0.666668740848	0.666668740852	0.198369201847
0.555557284053	0.111111456840	0.066123067296
0.333334370422	0.000000000000	0.000000000000
0.666668740848	0.666668740852	0.000000000000
0.111111456793	0.222222913585	0.132246134551
0.000000000000	0.000000000000	0.198369201847
0.222426398912	0.111456123222	0.263581991925
0.113627848187	0.224396347169	0.331877654550
0.555559732129	0.111353980626	0.263375005916
0.444578835547	0.222583341782	0.331623738362
0.333571903323	0.001095882252	0.387537817084
0.889070251895	0.111410081608	0.263224653064
0.778189806931	0.222262645155	0.331607141401
0.667015112829	-0.001026242912	0.387590935519
0.222377550127	0.444227458566	0.263438730540
0.110841331984	0.556351342671	0.331628177351
-0.000784182096	0.333226653144	0.387595220399
0.555341957894	0.444331056572	0.263511263073
0.442200268801	0.552354396527	0.331870144522
0.001084962406	0.000846430470	0.387548885683
0.889084433503	0.444766384229	0.263345874675
0.333272712356	0.336496065023	0.392289473701
0.000000000000	0.000000000000	0.000000000000
0.777443532208	0.888794587610	0.331455277075
0.888669675692	0.777913938400	0.263309351215
0.332730033214	0.668226240080	0.387600356080
0.444346801536	0.888333489114	0.331564453599
0.667034883928	0.666773450385	0.387502837107
-0.000475564474	0.666872630433	0.387761625055
0.777447925785	0.555946645527	0.332045476831
0.555421472308	0.777626560326	0.263154964091
0.666609430372	0.333225556056	0.387491580056

0.222222913631	0.111111456840	0.066123067296
0.222145884164	0.777783468677	0.263300614893
0.111489723413	0.890547614439	0.332213805720
0.666556036813	0.000092195866	0.296039199927
0.333334370426	0.666668740852	0.099184600903
0.444445827218	0.888891654437	0.231430735495
0.777780197640	0.888891654437	0.033061533648
0.888891654479	0.777780197692	0.165307668199
0.777780197640	0.888891654437	0.231430735495
0.000003918907	0.000573014777	0.296006132676
0.223801151396	0.114531352352	0.371426865926
0.333425464980	-0.000147999720	0.296024085392
0.556104130391	0.110589987772	0.371504329857
0.555557284057	0.777780197692	0.165307668199
0.666668740848	0.666668740852	0.099184600903
0.888816234314	0.445002652133	0.371364102480
0.000172340150	0.333695692872	0.296035254537
0.221114701376	0.446274177158	0.370971921815
0.333652819450	0.332540859593	0.296004078041
0.554876490468	0.444418789289	0.370691620659
0.667095810161	0.333544346637	0.295967662803
-0.000008151569	0.667521497055	0.296148677107
0.221439329764	0.778722021520	0.371444281280
0.333286029203	0.666357566414	0.295974412280
0.554815653849	0.776635907835	0.371327593837
0.665980649869	0.666400943762	0.295973591013
0.444445827218	0.888891654437	0.033061533648
0.889250882099	0.778586578155	0.371302138715
0.889385005008	0.111092170397	0.371357759019
0.111111456797	0.888891654437	0.231430735495
0.111111456795	0.555557284011	0.033061533648
0.000000000004	0.666668740852	0.099184600903
0.111111456793	0.222222913585	0.033061533648
0.000000000000	0.000000000000	0.099184600903
0.222222913631	0.111111456840	0.165307668199
0.111111456793	0.222222913585	0.231430735495
0.444445827215	0.222222913585	0.033061533648
0.333334370422	0.000000000000	0.099184600903
0.555557284053	0.111111456840	0.165307668199
0.444445827215	0.222222913585	0.231430735495
0.777780197636	0.222222913585	0.033061533648
0.666668740844	0.000000000000	0.099184600903
0.222222913635	0.777780197692	0.165307668199
0.777780197636	0.222222913585	0.231430735495
0.888891654475	0.111111456840	0.165307668199
0.222222913633	0.444445827266	0.165307668199

0.111111456795   0.555557284011   0.231430735495
0.444445827216   0.555557284011   0.033061533648
0.333334370424   0.333334370426   0.099184600903
0.555557284055   0.444445827266   0.165307668199
0.444445827216   0.555557284011   0.231430735495
0.777780197638   0.555557284011   0.033061533648
0.666668740846   0.333334370426   0.099184600903
0.888891654477   0.444445827266   0.165307668199
0.777780197638   0.555557284011   0.231430735495
0.111111456797   0.888891654437   0.033061533648
0.000000000002   0.333334370426   0.099184600903
0.402366570412   0.254967001266   0.477135403771
0.400670045142   0.403026151229   0.483107976818
0.252158843846   0.252122429288   0.483771856075

4. CH_2* and H* on CoGa

C Co Ga H
1.0
6.040950000000 -10.463232325983   0.000000000000
6.040950000000 10.463232325983   0.000000000000
0.000000000000   0.000000000000 24.864900000000
C Co Ga H
1 63 54   3
Selective dynamics
Direct
0.236062408946   0.299943223688   0.448976829820
0.444445827215   0.222222913585   0.132246134551
0.333334370422   0.000000000000   0.198369201847
0.666668740844   0.000000000000   0.000000000000
0.888891654475   0.111111456840   0.066123067296
0.777780197636   0.222222913585   0.132246134551
0.666668740844   0.000000000000   0.198369201847
0.000000000002   0.333334370426   0.000000000000
0.222222913633   0.444445827266   0.066123067296
0.111111456795   0.555557284011   0.132246134551
0.000000000002   0.333334370426   0.198369201847
0.333334370424   0.333334370426   0.000000000000
0.555557284055   0.444445827266   0.066123067296
0.444445827216   0.555557284011   0.132246134551
0.333334370424   0.333334370426   0.198369201847
0.666668740846   0.333334370426   0.000000000000
0.888891654477   0.444445827266   0.066123067296
0.777780197638   0.555557284011   0.132246134551
0.666668740846   0.333334370426   0.198369201847
0.000000000004   0.666668740852   0.000000000000
0.222222913635   0.777780197692   0.066123067296
0.111111456797   0.888891654437   0.132246134551

0.000000000004	0.666668740852	0.198369201847
0.333334370426	0.666668740852	0.000000000000
0.555557284057	0.777780197692	0.066123067296
0.444445827218	0.888891654437	0.132246134551
0.333334370426	0.666668740852	0.198369201847
0.888891654479	0.777780197692	0.066123067296
0.777780197640	0.888891654437	0.132246134551
0.666668740848	0.666668740852	0.198369201847
0.555557284053	0.111111456840	0.066123067296
0.333334370422	0.000000000000	0.000000000000
0.666668740848	0.666668740852	0.000000000000
0.111111456793	0.222222913585	0.132246134551
0.000000000000	0.000000000000	0.198369201847
0.222752213112	0.111593251740	0.263877093527
0.111002095420	0.223321118514	0.330281185707
0.555594959922	0.110974812840	0.263555957907
0.438842020765	0.226422134215	0.333459116117
0.346893493574	0.015605154100	0.388578368951
0.889570600662	0.111958788245	0.263428930996
0.780033755966	0.222057781576	0.330789732431
0.675176883973	-0.009293374654	0.388238994720
0.222110114140	0.444290745459	0.263458162102
0.109201044231	0.557209690489	0.333285148464
-0.006083959773	0.338506977001	0.387697914714
0.555027837526	0.443661757706	0.263571730417
0.440863669182	0.552430933853	0.331685039207
-0.006078856523	-0.005806193053	0.387870416704
0.888337354965	0.444508481756	0.263694453751
0.325591410815	0.333354052464	0.389994345220
0.000000000000	0.000000000000	0.000000000000
0.778242188342	0.887189123466	0.331719911409
0.888728492284	0.777540133971	0.263441236365
0.328687154314	0.669891207333	0.387940825011
0.445100894303	0.885898786435	0.330503286314
0.670794955549	0.670200442480	0.387828962753
0.003983998479	0.662926067969	0.387790496758
0.776309251152	0.555587025570	0.330916209695
0.554691026022	0.777258034304	0.263143513688
0.648176262827	0.319698303186	0.388760471319
0.222222913631	0.111111456840	0.066123067296
0.222272790145	0.778293184684	0.263547201620
0.111749281446	0.890925763106	0.331699288198
0.667967677857	-0.001323863766	0.296087550648
0.333334370426	0.666668740852	0.099184600903
0.444445827218	0.888891654437	0.231430735495
0.777780197640	0.888891654437	0.033061533648

0.888891654479	0.777780197692	0.165307668199
0.777780197640	0.888891654437	0.231430735495
-0.000151784657	0.000163788321	0.296063636728
0.216828048663	0.109940860473	0.372223090206
0.333898362366	0.000417076081	0.296742337788
0.565913462371	0.100396820673	0.368995546268
0.555557284057	0.777780197692	0.165307668199
0.666668740848	0.666668740852	0.099184600903
0.875537844170	0.441019679744	0.372712111299
0.001519168212	0.335922918658	0.296050450545
0.219642264915	0.446698757793	0.372085688700
0.331753011773	0.333725122437	0.295192503710
0.555903153449	0.449700301069	0.369666464741
0.665509748615	0.332249755248	0.296816823785
0.000143877636	0.667343038938	0.296242016996
0.226465693344	0.789018333358	0.372412730054
0.330830369971	0.664771093972	0.296105539688
0.551387885730	0.771663160973	0.371021554918
0.665118799053	0.666625892427	0.295997409853
0.444445827218	0.888891654437	0.033061533648
0.891377901436	0.774058832924	0.371247117455
0.893738192375	0.115102236518	0.370621543925
0.111111456797	0.888891654437	0.231430735495
0.111111456795	0.555557284011	0.033061533648
0.000000000004	0.666668740852	0.099184600903
0.111111456793	0.222222913585	0.033061533648
0.000000000000	0.000000000000	0.099184600903
0.222222913631	0.111111456840	0.165307668199
0.111111456793	0.222222913585	0.231430735495
0.444445827215	0.222222913585	0.033061533648
0.333334370422	0.000000000000	0.099184600903
0.555557284053	0.111111456840	0.165307668199
0.444445827215	0.222222913585	0.231430735495
0.777780197636	0.222222913585	0.033061533648
0.666668740844	0.000000000000	0.099184600903
0.222222913635	0.777780197692	0.165307668199
0.777780197636	0.222222913585	0.231430735495
0.888891654475	0.111111456840	0.165307668199
0.222222913633	0.444445827266	0.165307668199
0.111111456795	0.555557284011	0.231430735495
0.444445827216	0.555557284011	0.033061533648
0.333334370424	0.333334370426	0.099184600903
0.555557284055	0.444445827266	0.165307668199
0.444445827216	0.555557284011	0.231430735495
0.777780197638	0.555557284011	0.033061533648
0.666668740846	0.333334370426	0.099184600903

0.888891654477   0.444445827266   0.165307668199
0.777780197638   0.555557284011   0.231430735495
0.111111456797   0.888891654437   0.033061533648
0.000000000002   0.333334370426   0.099184600903
0.469050706875   0.196823715869   0.396061323746
0.305523743998   0.334859311404   0.482772131541
0.135892598992   0.256778981784   0.461497144127

5. **CH_2* on CoGa**

C Co Ga H
1.0
6.040950000000 -10.463232325983   0.000000000000
6.040950000000 10.463232325983   0.000000000000
0.000000000000   0.000000000000 24.864900000000
C Co Ga H
1 63 54   2
Selective dynamics
Direct
0.247425812962   0.298526051676   0.450663153642
0.444445827215   0.222222913585   0.132246134551
0.333334370422   0.000000000000   0.198369201847
0.666668740844   0.000000000000   0.000000000000
0.888891654475   0.111111456840   0.066123067296
0.777780197636   0.222222913585   0.132246134551
0.666668740844   0.000000000000   0.198369201847
0.000000000002   0.333334370426   0.000000000000
0.222222913633   0.444445827266   0.066123067296
0.111111456795   0.555557284011   0.132246134551
0.000000000002   0.333334370426   0.198369201847
0.333334370424   0.333334370426   0.000000000000
0.555557284055   0.444445827266   0.066123067296
0.444445827216   0.555557284011   0.132246134551
0.333334370424   0.333334370426   0.198369201847
0.666668740846   0.333334370426   0.000000000000
0.888891654477   0.444445827266   0.066123067296
0.777780197638   0.555557284011   0.132246134551
0.666668740846   0.333334370426   0.198369201847
0.000000000004   0.666668740852   0.000000000000
0.222222913635   0.777780197692   0.066123067296
0.111111456797   0.888891654437   0.132246134551
0.000000000004   0.666668740852   0.198369201847
0.333334370426   0.666668740852   0.000000000000
0.555557284057   0.777780197692   0.066123067296
0.444445827218   0.888891654437   0.132246134551
0.666668740848   0.666668740852   0.000000000000
0.888891654479   0.777780197692   0.066123067296
0.777780197640   0.888891654437   0.132246134551

0.666668740848	0.666668740852	0.198369201847
0.555557284053	0.111111456840	0.066123067296
0.333334370422	0.000000000000	0.000000000000
0.333334370426	0.666668740852	0.198369201847
0.111111456793	0.222222913585	0.132246134551
0.000000000000	0.000000000000	0.198369201847
0.222324682828	0.111457974071	0.263602592124
0.110948985918	0.222340163178	0.330779219451
0.555454942147	0.111064465020	0.263132429178
0.442989388121	0.223577251677	0.332371979714
0.333577058902	0.000434529608	0.387704619352
0.889044635015	0.111255067995	0.263456222874
0.777270418840	0.222174314166	0.331673124313
0.666672339589	-0.000551548503	0.387859582118
0.222258490562	0.444206386299	0.263622618361
0.111992483100	0.555548016522	0.332397367498
-0.002581857491	0.333474907776	0.387499915867
0.555261472237	0.444310665474	0.263234916168
0.442790945414	0.552470091717	0.332194176901
-0.001863099472	-0.002480639006	0.387514827038
0.888969035677	0.444658052961	0.263422416122
0.331664702940	0.333290925565	0.390288932988
0.000000000000	0.000000000000	0.000000000000
0.777118841266	0.888184955432	0.331614717338
0.889024419472	0.777667193782	0.263367995290
0.333235583506	0.667805282570	0.387716454640
0.444919551220	0.889120811554	0.331326037104
0.666996919154	0.666686351027	0.387461658761
0.001075625144	0.667205078666	0.387844797271
0.778535600718	0.555933768878	0.331326008358
0.555484673680	0.777757466113	0.263157451634
0.666898330136	0.333241215044	0.387516948967
0.222222913631	0.111111456840	0.066123067296
0.222452920874	0.777919280584	0.263341303337
0.111831864586	0.890229312601	0.332366731467
0.666696223082	0.000071118969	0.296131942673
0.444445827218	0.888891654437	0.231430735495
0.777780197640	0.888891654437	0.033061533648
0.333334370426	0.666668740852	0.099184600903
0.888891654479	0.777780197692	0.165307668199
0.777780197640	0.888891654437	0.231430735495
0.000032869446	-0.000135582888	0.296018758240
0.222619653754	0.112470375065	0.372982437365
0.333524544433	0.000467662484	0.296088106606
0.555658624948	0.110871057233	0.371258974330
0.555557284057	0.777780197692	0.165307668199

0.666668740848	0.666668740852	0.099184600903
0.888451781487	0.445401747391	0.371182144547
0.000170709309	0.333881639325	0.296024727355
0.222218482650	0.444586854439	0.373120678873
0.332492633450	0.332795930012	0.295621337949
0.556932511264	0.444939012623	0.370242330279
0.666015476401	0.332890928790	0.295930909262
0.001078221798	0.667322860514	0.296231526224
0.223279445622	0.779010413392	0.371203908085
0.333434980552	0.666265605773	0.296081200945
0.555114555271	0.777270637541	0.371167858208
0.666016072718	0.666385416196	0.295966387934
0.444445827218	0.888891654437	0.033061533648
0.888551457160	0.776483658924	0.371093349574
0.888517463735	0.110692929648	0.371166640670
0.111111456797	0.888891654437	0.231430735495
0.111111456795	0.555557284011	0.033061533648
0.000000000004	0.666668740852	0.099184600903
0.111111456793	0.222222913585	0.033061533648
0.000000000000	0.000000000000	0.099184600903
0.222222913631	0.111111456840	0.165307668199
0.111111456793	0.222222913585	0.231430735495
0.444445827215	0.222222913585	0.033061533648
0.333334370422	0.000000000000	0.099184600903
0.555557284053	0.111111456840	0.165307668199
0.444445827215	0.222222913585	0.231430735495
0.777780197636	0.222222913585	0.033061533648
0.666668740844	0.000000000000	0.099184600903
0.888891654475	0.111111456840	0.165307668199
0.222222913635	0.777780197692	0.165307668199
0.777780197636	0.222222913585	0.231430735495
0.222222913633	0.444445827266	0.165307668199
0.111111456795	0.555557284011	0.231430735495
0.444445827216	0.555557284011	0.033061533648
0.333334370424	0.333334370426	0.099184600903
0.555557284055	0.444445827266	0.165307668199
0.444445827216	0.555557284011	0.231430735495
0.777780197638	0.555557284011	0.033061533648
0.666668740846	0.333334370426	0.099184600903
0.888891654477	0.444445827266	0.165307668199
0.777780197638	0.555557284011	0.231430735495
0.111111456797	0.888891654437	0.033061533648
0.000000000002	0.333334370426	0.099184600903
0.319641348008	0.338531918144	0.483300810661
0.147971771278	0.250222539370	0.464540038737

6. CH* and H* on CoGa

```
C    Co   Ga   H
   1.00000000000000
     6.0409499999999996  -10.4632323259830002    0.0000000000000000
     6.0409499999999996   10.4632323259830002    0.0000000000000000
     0.0000000000000000    0.0000000000000000   24.8648999999999987
   C    Co   Ga   H
     1    63    54    2
Selective dynamics
Direct
  0.1621751227405421  0.3224346427265906  0.4045187256357927
  0.4444518345156681  0.2221800125049853  0.1320548379976714
  0.3333108923942163  0.9999637014425744  0.1981868992004578
  0.6666534905699990  0.9999020264476  0.9999089826193313
  0.8888403289486817  0.1110762798845428  0.0659886187597962
  0.7777145727034522  0.2221831144784545  0.1320891659724666
  0.6666150925427843  0.9999462971532083  0.1982304036603395
  0.9999619203606613  0.3333226055640068  0.9998865998574473
  0.2221979737564368  0.4444558700021020  0.0659260677791025
  0.1110555560057733  0.5555917887777753  0.1320332826466739
  0.9998267252007622  0.3332744054120056  0.1980503840917009
  0.3333310046162197  0.3333261531041032  0.9998699855008866
  0.5555745228890956  0.4444562936127596  0.0659673446673423
  0.4444816159046567  0.5556163913013492  0.1320295942525647
  0.3333323774123400  0.3332893567752180  0.1978380930611223
  0.6666645504591173  0.3333294447933497  0.9999015583795625
  0.8888558573355709  0.4444440774701164  0.0659835651505531
  0.7777645345795676  0.5555760804465706  0.1320917867324667
  0.6666316471892273  0.3333118727000155  0.1981751172762287
  0.9999816148798928  0.6666723624782307  0.9999010161927657
  0.2222072530735488  0.7777906449456538  0.0659904892997858
  0.1110664770452061  0.8888671636820149  0.1320934532236819
  0.9999222767020157  0.6667201528192166  0.1981693243664751
  0.3333337339466738  0.6666924388410038  0.9998927421132251
  0.5555594524277723  0.7777975211925607  0.0660002908564650
  0.4444409250082231  0.8888999366334573  0.1321067607444562
  0.6666753640287837  0.6666824295480964  0.9999033802667725
  0.8888687252085532  0.7777768022576552  0.0660053721682274
  0.7777415451055560  0.8888710878322073  0.1321187042684429
  0.6666714310499984  0.6667071863756533  0.1982061652737030
  0.5555436421199005  0.1110931046603199  0.0660023423583240
  0.3333239357776492  0.9999838477944536  0.9999029586373425
  0.3333442683024472  0.6667630985216704  0.1981080688331431
  0.1110177017089882  0.2221486718126613  0.1320039930314749
  0.9998940979501185  0.9999100812823372  0.1981852413459251
  0.2204205139460677  0.1105454461254066  0.2635703821223049
```

0.1101593850941068	0.2192532333868762	0.3371712106401549
0.5559153734688228	0.1117171918302097	0.2633587757419504
0.4431804033140558	0.2238897987268651	0.3332684518534682
0.3352031782804364	0.9894697691225820	0.3863521152399344
0.8903177173832265	0.1104971349043529	0.2636549054356269
0.7812032478421086	0.2238559426225885	0.3333157899325545
0.6551488886041095	0.9897037135630078	0.3863592841303288
0.2229520684240400	0.4458999617796823	0.2632183239581092
0.1124597400775768	0.5535347739784120	0.3306180548597455
0.0309635088131096	0.3486748771341771	0.3840288133818216
0.5553753023667474	0.4436444651614389	0.2630619816362709
0.4418762286631098	0.5536830778407975	0.3305505903552964
-0.0002817850174222	-0.0009689741280396	0.3881046248246113
0.8883852229108965	0.4435725885598889	0.2631097299960032
0.3194847003984152	0.3488770081200816	0.3838292053426713
0.9999671715826679	0.9999709260182144	0.9999000749086733
0.7752722838264386	0.8883848629977450	0.3323659167476031
0.8889583377253724	0.7777022429766264	0.2632269586123913
0.4483808048383217	0.8958448145432447	0.4188460795975629
0.4478808837279862	0.8952288203067835	0.3278036382375141
0.6657978040756762	0.6691576197042810	0.3870981666629420
0.0038539951715171	0.6690138196699125	0.3870924760208630
0.7783676954510799	0.5560273913804344	0.3315345648088154
0.5534299533131970	0.7773421215792441	0.2643534479275513
0.6732406378980226	0.3452154073610355	0.3875892265355537
0.2221926285175542	0.1110676838123226	0.0659634275685619
0.2240882134586092	0.7773053392945671	0.2643275655884618
0.1135891170263318	0.8883041704201053	0.3323274493677840
0.6698636632747770	0.0025717716946860	0.2947639084943359
0.4444464818277609	0.8888990827176571	0.2312578574248931
0.7777594290226659	0.8888822311316673	0.0329589555008951
0.3333334739426519	0.6667095854783298	0.0990169692568870
0.8888426842243220	0.7777815701391049	0.1651608399236579
0.7777089602693437	0.8888464240391230	0.2312927754339142
-0.0008914591416278	-0.0022066629441148	0.2966964528270797
0.2284618556924655	0.1120017021249945	0.3718520791835179
0.3329922705162073	0.0025649830759991	0.2947508703926133
0.5609613604621247	0.1213975199351660	0.3750143013482835
0.5555657038770079	0.7778077531902383	0.1651581977723495
0.6666750386672078	0.6666908536347691	0.0990505792205667
0.8945349711000553	0.4449736617721384	0.3722842308502863
0.0004069587614128	0.3310057427279313	0.2942383008531323
0.2655581409225017	0.5299074954541659	0.3941490530692641
0.3309685738606816	0.3311323181920038	0.2940160276304682
0.5518772509646074	0.4451213833080165	0.3720523587870954
0.6666237191927676	0.3329189187518211	0.2961135463091894

0.0001175777497079	0.6661431975136688	0.2957299652399692
0.2259021513698163	0.7663955695968638	0.3714170484273236
0.3330571598592201	0.6656891866109205	0.3002526039583393
0.5410824045649194	0.7665424180756137	0.3714275366614548
0.6664128266523155	0.6662311840216936	0.2957301707034982
0.4444407920145679	0.8888957998611104	0.0329543945874491
0.8884390448844764	0.7762596882280179	0.3716769093414780
0.8843346055734770	0.1122508001812341	0.3719853875517015
0.1110273152083252	0.8888675851858849	0.2312347189153670
0.1110855899324577	0.5555699930168885	0.0329279257144535
0.9999658015704611	0.6666821740815649	0.0990425592464348
0.1110688963398800	0.2221912337043292	0.0329124660911120
0.9999438857721046	0.9999530864194455	0.0990418167467837
0.2221581450982058	0.1110062534065577	0.1650733835080089
0.1109070883100074	0.2220463303128994	0.2310386689015829
0.4444460344378740	0.2222025591466519	0.0329308800915555
0.3333191745281212	0.9999763867936622	0.0990461704774432
0.5555318367332163	0.1110721496211013	0.1651554089427734
0.4444580708722228	0.2221394535724528	0.2311833640116845
0.7777480029584325	0.2222047930828950	0.0329479611162213
0.6666401376196802	0.9999793884038795	0.0990662781065481
0.8887851015187707	0.1110284209874450	0.1651277561754370
0.2221956514235401	0.7778171788145656	0.1651260736044478
0.7776335342803478	0.2221280822253249	0.2312130375484784
0.2221652685151270	0.4444741440208873	0.1649462414664455
0.1109581715433379	0.5556625213623505	0.2310597344492606
0.4444583320952304	0.5555829350787675	0.0329207284373254
0.3333285168696491	0.3333177037547728	0.0989335603565706
0.5556089780202100	0.4444751196912264	0.1650826430424601
0.4445203783790177	0.5556596833653416	0.2311301148030154
0.7777724542165672	0.5555634066522330	0.0329478109735533
0.6666548612916614	0.3333224456786610	0.0990416996703516
0.8888063566727880	0.4444486209996654	0.1651074910458803
0.7777421177087689	0.5556127979582399	0.2312248219190280
0.1110879531444553	0.8888760633337682	0.0329480844995800
0.9999289197815671	0.3333112384883208	0.0989984528111236
0.1501598368766136	0.2970233225773207	0.4481888974087458
0.3071385388136166	0.6126446044566464	0.4487167933847618

7. CH* on CoGa

C Co Ga H
1.0

6.040950000000	-10.463232325983	0.000000000000
6.040950000000	10.463232325983	0.000000000000
0.000000000000	0.000000000000	24.864900000000

C Co Ga H

```
  1  63  54   1
Selective dynamics
Direct
  0.345410601948  0.352757291059  0.456720814701
  0.444445827215  0.222222913585  0.132246134551
  0.333334370422  0.000000000000  0.198369201847
  0.666668740844  0.000000000000  0.000000000000
  0.888891654475  0.111111456840  0.066123067296
  0.777780197636  0.222222913585  0.132246134551
  0.666668740844  0.000000000000  0.198369201847
  0.000000000002  0.333334370426  0.000000000000
  0.222222913633  0.444445827266  0.066123067296
  0.111111456795  0.555557284011  0.132246134551
  0.000000000002  0.333334370426  0.198369201847
  0.333334370424  0.333334370426  0.000000000000
  0.555557284055  0.444445827266  0.066123067296
  0.444445827216  0.555557284011  0.132246134551
  0.333334370424  0.333334370426  0.198369201847
  0.666668740846  0.333334370426  0.000000000000
  0.888891654477  0.444445827266  0.066123067296
  0.777780197638  0.555557284011  0.132246134551
  0.666668740846  0.333334370426  0.198369201847
  0.000000000004  0.666668740852  0.000000000000
  0.222222913635  0.777780197692  0.066123067296
  0.111111456797  0.888891654437  0.132246134551
  0.000000000004  0.666668740852  0.198369201847
  0.333334370426  0.666668740852  0.000000000000
  0.555557284057  0.777780197692  0.066123067296
  0.444445827218  0.888891654437  0.132246134551
  0.666668740848  0.666668740852  0.000000000000
  0.888891654479  0.777780197692  0.066123067296
  0.777780197640  0.888891654437  0.132246134551
  0.666668740848  0.666668740852  0.198369201847
  0.555557284053  0.111111456840  0.066123067296
  0.333334370422  0.000000000000  0.000000000000
  0.333334370426  0.666668740852  0.198369201847
  0.111111456793  0.222222913585  0.132246134551
  0.000000000000  0.000000000000  0.198369201847
  0.222382622192  0.111379627405  0.263605516152
  0.113432358345  0.223471564299  0.331332282234
  0.555526384888  0.111074861910  0.263138536446
  0.443166411182  0.223580219427  0.331274922089
  0.332475682313  0.999743366508  0.387422030996
  0.888788412052  0.111169924368  0.263164062150
  0.777572503298  0.221931994853  0.331594287277
  0.666480436318  -0.000015040704   0.387709397905
```

0.222306276699	0.444355615896	0.263641870335
0.111524408141	0.555083473009	0.332471204556
-0.000624199348	0.333533294636	0.387516032883
0.555420146353	0.444351017408	0.263630728620
0.443246094289	0.553396746377	0.331102349248
0.000234339039	-0.000595327091	0.387517870723
0.888859012385	0.444533201407	0.263405187084
0.333907516188	0.334692439319	0.391918159455
0.000000000000	0.000000000000	0.000000000000
0.777640868148	0.889040380975	0.331509779378
0.888815812685	0.777700067528	0.263362919800
0.332869395142	0.666647632991	0.387473491519
0.444701258889	0.889081641036	0.331637663051
0.667602628013	0.667113983223	0.387509966182
0.000059847356	0.666801751940	0.387881075514
0.776505425943	0.555018604714	0.332480348538
0.555479981666	0.777761956327	0.263250194548
0.667298555433	0.333839060525	0.387533647648
0.222222913631	0.111111456840	0.066123067296
0.221847355996	0.777643633901	0.263348034082
0.111541434281	0.889984641721	0.332401407976
0.666560810235	0.000019485509	0.295961083417
0.444445827218	0.888891654437	0.231430735495
0.777780197640	0.888891654437	0.033061533648
0.333334370426	0.666668740852	0.099184600903
0.888891654479	0.777780197692	0.165307668199
0.777780197640	0.888891654437	0.231430735495
0.000173100035	0.000310924314	0.295956375546
0.223337118322	0.113698486931	0.373422882317
0.333436489022	0.000238679139	0.295934834420
0.555268821407	0.111382291739	0.370829028400
0.555557284057	0.777780197692	0.165307668199
0.666668740848	0.666668740852	0.099184600903
0.666283461225	0.333169302018	0.295986903905
0.000208771062	0.333382804669	0.295981078898
0.222620436982	0.443310491193	0.373581226692
0.333302109071	0.333445547029	0.295584648522
0.553806816552	0.443662081628	0.373540245208
0.888675478876	0.444337110958	0.371270396539
0.999720392264	0.666573899951	0.296300298137
0.222579567658	0.777994842425	0.371195349259
0.333198917342	0.666550143368	0.295933301715
0.555403594533	0.777468357069	0.370782037960
0.444445827218	0.888891654437	0.033061533648
0.666283414370	0.666389925388	0.296003836458
0.889052212259	0.110978768596	0.370730665907

0.111111456797   0.888891654437   0.231430735495
0.777780197636   0.222222913585   0.231430735495
0.000000000004   0.666668740852   0.099184600903
0.888862151566   0.777997768295   0.371246798495
0.111111456793   0.222222913585   0.033061533648
0.000000000000   0.000000000000   0.099184600903
0.222222913631   0.111111456840   0.165307668199
0.111111456793   0.222222913585   0.231430735495
0.444445827215   0.222222913585   0.033061533648
0.333334370422   0.000000000000   0.099184600903
0.555557284053   0.111111456840   0.165307668199
0.444445827215   0.222222913585   0.231430735495
0.777780197636   0.222222913585   0.033061533648
0.666668740844   0.000000000000   0.099184600903
0.888891654475   0.111111456840   0.165307668199
0.111111456795   0.555557284011   0.033061533648
0.000000000002   0.333334370426   0.099184600903
0.222222913633   0.444445827266   0.165307668199
0.111111456795   0.555557284011   0.231430735495
0.444445827216   0.555557284011   0.033061533648
0.333334370424   0.333334370426   0.099184600903
0.555557284055   0.444445827266   0.165307668199
0.444445827216   0.555557284011   0.231430735495
0.777780197638   0.555557284011   0.033061533648
0.666668740846   0.333334370426   0.099184600903
0.888891654477   0.444445827266   0.165307668199
0.777780197638   0.555557284011   0.231430735495
0.111111456797   0.888891654437   0.033061533648
0.222222913635   0.777780197692   0.165307668199
0.358556679403   0.373716645652   0.500225291372

8. C* and H* on CoGa

C Co Ga H
1.0
6.040950000000 -10.463232325983   0.000000000000
6.040950000000 10.463232325983   0.000000000000
0.000000000000   0.000000000000 24.864900000000
C Co Ga H
1 63 54   1
Selective dynamics
Direct
0.321937677845   0.346386586103   0.455366797053
0.444445827215   0.222222913585   0.132246134551
0.333334370422   0.000000000000   0.198369201847
0.666668740844   0.000000000000   0.000000000000
0.888891654475   0.111111456840   0.066123067296
0.777780197636   0.222222913585   0.132246134551

0.666668740844	0.000000000000	0.198369201847
0.000000000002	0.333334370426	0.000000000000
0.222222913633	0.444445827266	0.066123067296
0.111111456795	0.555557284011	0.132246134551
0.000000000002	0.333334370426	0.198369201847
0.333334370424	0.333334370426	0.000000000000
0.555557284055	0.444445827266	0.066123067296
0.444445827216	0.555557284011	0.132246134551
0.333334370424	0.333334370426	0.198369201847
0.666668740846	0.333334370426	0.000000000000
0.888891654477	0.444445827266	0.066123067296
0.777780197638	0.555557284011	0.132246134551
0.666668740846	0.333334370426	0.198369201847
0.000000000004	0.666668740852	0.000000000000
0.222222913635	0.777780197692	0.066123067296
0.111111456797	0.888891654437	0.132246134551
0.000000000004	0.666668740852	0.198369201847
0.333334370426	0.666668740852	0.000000000000
0.555557284057	0.777780197692	0.066123067296
0.444445827218	0.888891654437	0.132246134551
0.666668740848	0.666668740852	0.000000000000
0.888891654479	0.777780197692	0.066123067296
0.777780197640	0.888891654437	0.132246134551
0.666668740848	0.666668740852	0.198369201847
0.555557284053	0.111111456840	0.066123067296
0.333334370422	0.000000000000	0.000000000000
0.333334370426	0.666668740852	0.198369201847
0.111111456793	0.222222913585	0.132246134551
0.000000000000	0.000000000000	0.198369201847
0.223084536193	0.111827986513	0.263910078152
0.115793526860	0.224931981804	0.332457200576
0.555052451854	0.111708994784	0.263553124546
0.442560591636	0.224399080296	0.335456680004
0.332941109379	0.999396562950	0.387742226742
0.889396377072	0.111510436884	0.263310083998
0.777671436602	0.222470200473	0.331152106896
0.667608847794	-0.000501585850	0.388036208795
0.222393984718	0.444404877852	0.263473383818
0.111970599574	0.555283217065	0.332030428880
0.001063610065	0.334919438575	0.387538308218
0.555093665920	0.443810747755	0.263900585511
0.442028333463	0.551277752722	0.332336448800
-0.006776925875	-0.002787944069	0.387923175370
0.888654975361	0.444305373655	0.263452957105
0.336353994194	0.330983409417	0.392969319816
0.000000000000	0.000000000000	0.000000000000

0.777799381394	0.889261075092	0.331353993332
0.889109687971	0.777581393122	0.263368063024
0.332357660635	0.666076389403	0.387585384401
0.444602598857	0.889432483659	0.331163748282
0.670125531492	0.674227487288	0.387957757502
0.001121303674	0.666253818573	0.387866520808
0.779277827870	0.556427075294	0.331525668609
0.555281822339	0.777383612832	0.263315943681
0.667665152534	0.334603190266	0.387736276446
0.222222913631	0.111111456840	0.066123067296
0.222382125733	0.778042856401	0.263387076835
0.110519141191	0.887682711895	0.331543848237
0.666816759621	0.000121018871	0.296150652358
0.444445827218	0.888891654437	0.231430735495
0.777780197640	0.888891654437	0.033061533648
0.333334370426	0.666668740852	0.099184600903
0.888891654479	0.777780197692	0.165307668199
0.777780197640	0.888891654437	0.231430735495
0.000017305059	0.000224142599	0.296109359419
0.213466844010	0.104879791814	0.370492998026
0.333435786005	0.000753914959	0.296218094486
0.557157791687	0.110189020674	0.370435868167
0.555557284057	0.777780197692	0.165307668199
0.666668740848	0.666668740852	0.099184600903
0.665947789642	0.333282143800	0.296218224117
0.000496590326	0.333629427074	0.296064108003
0.223526828644	0.443641040004	0.372098406772
0.333276454997	0.333543551033	0.294408892492
0.562298002582	0.454132200326	0.370281958602
0.888802173947	0.444011531370	0.371493116129
0.999325054190	0.666089057317	0.296125470084
0.223298597912	0.778306909410	0.371471754955
0.333268253506	0.666432344516	0.296040291571
0.553966079703	0.778193997418	0.370685401243
0.444445827218	0.888891654437	0.033061533648
0.666589195270	0.666910874331	0.296165996475
0.888927746722	0.113135753258	0.370688258128
0.111111456797	0.888891654437	0.231430735495
0.777780197636	0.222222913585	0.231430735495
0.000000000004	0.666668740852	0.099184600903
0.889873237211	0.777275995284	0.371140928683
0.111111456793	0.222222913585	0.033061533648
0.000000000000	0.000000000000	0.099184600903
0.222222913631	0.111111456840	0.165307668199
0.111111456793	0.222222913585	0.231430735495
0.444445827215	0.222222913585	0.033061533648

0.333334370422   0.000000000000   0.099184600903
0.555557284053   0.111111456840   0.165307668199
0.444445827215   0.222222913585   0.231430735495
0.777780197636   0.222222913585   0.033061533648
0.666668740844   0.000000000000   0.099184600903
0.888891654475   0.111111456840   0.165307668199
0.111111456795   0.555557284011   0.033061533648
0.000000000002   0.333334370426   0.099184600903
0.222222913633   0.444445827266   0.165307668199
0.111111456795   0.555557284011   0.231430735495
0.444445827216   0.555557284011   0.033061533648
0.333334370424   0.333334370426   0.099184600903
0.555557284055   0.444445827266   0.165307668199
0.444445827216   0.555557284011   0.231430735495
0.777780197638   0.555557284011   0.033061533648
0.666668740846   0.333334370426   0.099184600903
0.888891654477   0.444445827266   0.165307668199
0.777780197638   0.555557284011   0.231430735495
0.111111456797   0.888891654437   0.033061533648
0.222222913635   0.777780197692   0.165307668199
0.420411820523   0.252449969867   0.397113748380

9. CO_2 on CoGa

C Co Ga O
1.0
6.040950000000 -10.463232325983   0.000000000000
6.040950000000 10.463232325983   0.000000000000
0.000000000000   0.000000000000 24.864900000000
C Co Ga O
1 63 54   2
Selective dynamics
Direct
0.258579157622   0.313724928496   0.467399681934
0.444445827215   0.222222913585   0.132246134551
0.333334370422   0.000000000000   0.198369201847
0.666668740844   0.000000000000   0.000000000000
0.888891654475   0.111111456840   0.066123067296
0.777780197636   0.222222913585   0.132246134551
0.666668740844   0.000000000000   0.198369201847
0.000000000002   0.333334370426   0.000000000000
0.222222913633   0.444445827266   0.066123067296
0.111111456795   0.555557284011   0.132246134551
0.000000000002   0.333334370426   0.198369201847
0.333334370424   0.333334370426   0.000000000000
0.555557284055   0.444445827266   0.066123067296
0.444445827216   0.555557284011   0.132246134551
0.333334370424   0.333334370426   0.198369201847

0.666668740846	0.333334370426	0.000000000000
0.888891654477	0.444445827266	0.066123067296
0.777780197638	0.555557284011	0.132246134551
0.666668740846	0.333334370426	0.198369201847
0.000000000004	0.666668740852	0.000000000000
0.222222913635	0.777780197692	0.066123067296
0.111111456797	0.888891654437	0.132246134551
0.000000000004	0.666668740852	0.198369201847
0.333334370426	0.666668740852	0.000000000000
0.555557284057	0.777780197692	0.066123067296
0.444445827218	0.888891654437	0.132246134551
0.666668740848	0.666668740852	0.000000000000
0.888891654479	0.777780197692	0.066123067296
0.777780197640	0.888891654437	0.132246134551
0.666668740848	0.666668740852	0.198369201847
0.555557284053	0.111111456840	0.066123067296
0.333334370422	0.000000000000	0.000000000000
0.333334370426	0.666668740852	0.198369201847
0.111111456793	0.222222913585	0.132246134551
0.000000000000	0.000000000000	0.198369201847
0.222832509326	0.111904770324	0.263302663165
0.111482631355	0.222618292122	0.330481209223
0.555852985225	0.110965387827	0.263098952122
0.445432329002	0.222054563634	0.330711454978
0.334266345866	0.001007056026	0.387327631163
0.888854894833	0.111199155515	0.263142117604
0.778731657048	0.222523972314	0.331644122382
0.666402739995	-0.000930319836	0.387691224060
0.222984958287	0.444214652101	0.263291851328
0.111551005834	0.555154879687	0.332066275170
-0.000092301846	0.333363907982	0.388087749788
0.554651354772	0.443961490165	0.263338977438
0.444767683680	0.555692754849	0.330822469174
0.000637649175	0.000187293699	0.387649147171
0.889147661175	0.444551952298	0.263288193819
0.327350355893	0.330352641284	0.395658774664
0.000000000000	0.000000000000	0.000000000000
0.778402363433	0.889193975726	0.331673381550
0.889112385655	0.777753735472	0.263299898087
0.333466646171	0.665736578619	0.387448902959
0.444981102781	0.889067421687	0.331857580478
0.666771409038	0.664046746693	0.387523863927
0.000378573139	0.666913742019	0.387846294440
0.777464137559	0.555384653816	0.332040241230
0.555745804318	0.777933415375	0.263254614236
0.667243250631	0.335673344449	0.387543343794

0.222222913631	0.111111456840	0.066123067296
0.221957419178	0.777623965645	0.263312496041
0.111242903190	0.889057421712	0.331987616416
0.666809947366	0.000123342469	0.296062072101
0.444445827218	0.888891654437	0.231430735495
0.777780197640	0.888891654437	0.033061533648
0.333334370426	0.666668740852	0.099184600903
0.888891654479	0.777780197692	0.165307668199
0.777780197640	0.888891654437	0.231430735495
0.000042788159	-0.000059899265	0.295979014959
0.223158922554	0.112723640725	0.370120074742
0.333430494500	-0.000380943173	0.295852354044
0.557169421714	0.112479460467	0.371409544438
0.555557284057	0.777780197692	0.165307668199
0.666668740848	0.666668740852	0.099184600903
0.889433109380	0.444643590564	0.371645412477
0.000200132450	0.333629100579	0.296157907900
0.222801511304	0.443147026188	0.370490367923
0.333329426801	0.332940361992	0.298714464225
0.551550432287	0.441861039269	0.370684368395
0.667305394672	0.333353310088	0.295893819643
-0.000099763979	0.666339790808	0.296209188355
0.222760949160	0.777947308768	0.371040458866
0.333326623271	0.667003261896	0.295892593405
0.556335990294	0.776751611278	0.371381265360
0.667079876100	0.666867879973	0.295953855555
0.444445827218	0.888891654437	0.033061533648
0.889305928273	0.778090673674	0.371375239715
0.889091233053	0.111092862970	0.371094791657
0.111111456797	0.888891654437	0.231430735495
0.111111456795	0.555557284011	0.033061533648
0.000000000004	0.666668740852	0.099184600903
0.111111456793	0.222222913585	0.033061533648
0.000000000000	0.000000000000	0.099184600903
0.222222913631	0.111111456840	0.165307668199
0.111111456793	0.222222913585	0.231430735495
0.444445827215	0.222222913585	0.033061533648
0.333334370422	0.000000000000	0.099184600903
0.555557284053	0.111111456840	0.165307668199
0.444445827215	0.222222913585	0.231430735495
0.777780197636	0.222222913585	0.033061533648
0.666668740844	0.000000000000	0.099184600903
0.888891654475	0.111111456840	0.165307668199
0.222222913635	0.777780197692	0.165307668199
0.777780197636	0.222222913585	0.231430735495
0.222222913633	0.444445827266	0.165307668199

```
  0.111111456795   0.555557284011   0.231430735495
  0.444445827216   0.555557284011   0.033061533648
  0.333334370424   0.333334370426   0.099184600903
  0.555557284055   0.444445827266   0.165307668199
  0.444445827216   0.555557284011   0.231430735495
  0.777780197638   0.555557284011   0.033061533648
  0.666668740846   0.333334370426   0.099184600903
  0.888891654477   0.444445827266   0.165307668199
  0.777780197638   0.555557284011   0.231430735495
  0.111111456797   0.888891654437   0.033061533648
  0.000000000002   0.333334370426   0.099184600903
  0.161545540058   0.284792098301   0.492035840094
  0.375556769328   0.348708896582   0.472906017381
```

10. CO* and O* on CoGa

```
C Co Ga O
1.0
  6.040950000000 -10.463232325983   0.000000000000
  6.040950000000  10.463232325983   0.000000000000
  0.000000000000   0.000000000000  24.864900000000
C Co Ga O
  1  63  54   2
Selective dynamics
Direct
  0.277991433942   0.315837951541   0.459202847928
  0.444445827215   0.222222913585   0.132246134551
  0.333334370422   0.000000000000   0.198369201847
  0.666668740844   0.000000000000   0.000000000000
  0.888891654475   0.111111456840   0.066123067296
  0.777780197636   0.222222913585   0.132246134551
  0.666668740844   0.000000000000   0.198369201847
  0.000000000002   0.333334370426   0.000000000000
  0.222222913633   0.444445827266   0.066123067296
  0.111111456795   0.555557284011   0.132246134551
  0.000000000002   0.333334370426   0.198369201847
  0.333334370424   0.333334370426   0.000000000000
  0.555557284055   0.444445827266   0.066123067296
  0.444445827216   0.555557284011   0.132246134551
  0.333334370424   0.333334370426   0.198369201847
  0.666668740846   0.333334370426   0.000000000000
  0.888891654477   0.444445827266   0.066123067296
  0.777780197638   0.555557284011   0.132246134551
  0.666668740846   0.333334370426   0.198369201847
  0.000000000004   0.666668740852   0.000000000000
  0.222222913635   0.777780197692   0.066123067296
  0.111111456797   0.888891654437   0.132246134551
  0.000000000004   0.666668740852   0.198369201847
```

0.333334370426	0.666668740852	0.000000000000
0.555557284057	0.777780197692	0.066123067296
0.444445827218	0.888891654437	0.132246134551
0.666668740848	0.666668740852	0.000000000000
0.888891654479	0.777780197692	0.066123067296
0.777780197640	0.888891654437	0.132246134551
0.666668740848	0.666668740852	0.198369201847
0.555557284053	0.111111456840	0.066123067296
0.333334370422	0.000000000000	0.000000000000
0.333334370426	0.666668740852	0.198369201847
0.111111456793	0.222222913585	0.132246134551
0.000000000000	0.000000000000	0.198369201847
0.223948301280	0.112295280182	0.264030963358
0.112678087038	0.220929867294	0.331072969391
0.554293417763	0.111015034236	0.263547018127
0.438590542386	0.216951026785	0.334556174276
0.328851902553	0.011696948250	0.388532973292
0.888962772423	0.111123911026	0.263032610458
0.774509632603	0.222584885278	0.331904503486
0.672375957201	0.003972096520	0.388371311804
0.222497635619	0.443117032399	0.263913357642
0.109078153867	0.550021372693	0.332490733869
0.009124398406	0.343147479583	0.387771601183
0.555340288032	0.445663520089	0.264695399598
0.444937972793	0.557676345900	0.330042259884
-0.010587746618	-0.003905538448	0.388036294181
0.887917585329	0.443498634711	0.263788479817
0.338856950848	0.313901282230	0.396115872652
0.000000000000	0.000000000000	0.000000000000
0.779863608942	0.893791868505	0.330706435929
0.890156378147	0.777493238913	0.263328695061
0.324600126983	0.656036657930	0.387764487470
0.440343393392	0.885738867393	0.330398460597
0.680711788873	0.697810677686	0.388390029024
-0.002457990894	0.658458927348	0.388157206842
0.780259145987	0.556412553582	0.330860394254
0.554806501639	0.778255218307	0.263108694661
0.652467670413	0.322230826706	0.387457630516
0.222222913631	0.111111456840	0.066123067296
0.222223091523	0.778009116908	0.263266689655
0.110912841850	0.885114107404	0.330788628311
0.665965204671	0.000084489707	0.296211185234
0.444445827218	0.888891654437	0.231430735495
0.777780197640	0.888891654437	0.033061533648
0.333334370426	0.666668740852	0.099184600903
0.888891654479	0.777780197692	0.165307668199

0.777780197640	0.888891654437	0.231430735495
0.000675347711	-0.000574281038	0.296021940013
0.210832955465	0.097684809936	0.370907935101
0.332579134292	-0.000483782403	0.296412987002
0.555053740810	0.099345954417	0.369619737611
0.555557284057	0.777780197692	0.165307668199
0.666668740848	0.666668740852	0.099184600903
0.880570655100	0.437528337116	0.372969067848
-0.001275941731	0.331438050955	0.296141491574
0.231276092591	0.435804371553	0.372200748345
0.334945209184	0.333997487589	0.298010906981
0.573048005135	0.480483851544	0.369938970519
0.664643109846	0.333766697928	0.295861188721
0.001275573603	0.664227429849	0.296225170992
0.218179515975	0.770437458268	0.370747041163
0.330507259098	0.664903083338	0.295924351774
0.546136305110	0.777743472613	0.370269196893
0.669648590433	0.671663166488	0.295877070738
0.444445827218	0.888891654437	0.033061533648
0.895608116976	0.777769079201	0.370314131278
0.892198951366	0.119312834162	0.370813332281
0.111111456797	0.888891654437	0.231430735495
0.111111456795	0.555557284011	0.033061533648
0.000000000004	0.666668740852	0.099184600903
0.111111456793	0.222222913585	0.033061533648
0.000000000000	0.000000000000	0.099184600903
0.222222913631	0.111111456840	0.165307668199
0.111111456793	0.222222913585	0.231430735495
0.444445827215	0.222222913585	0.033061533648
0.333334370422	0.000000000000	0.099184600903
0.555557284053	0.111111456840	0.165307668199
0.444445827215	0.222222913585	0.231430735495
0.777780197636	0.222222913585	0.033061533648
0.666668740844	0.000000000000	0.099184600903
0.888891654475	0.111111456840	0.165307668199
0.222222913635	0.777780197692	0.165307668199
0.777780197636	0.222222913585	0.231430735495
0.222222913633	0.444445827266	0.165307668199
0.111111456795	0.555557284011	0.231430735495
0.444445827216	0.555557284011	0.033061533648
0.333334370424	0.333334370426	0.099184600903
0.555557284055	0.444445827266	0.165307668199
0.444445827216	0.555557284011	0.231430735495
0.777780197638	0.555557284011	0.033061533648
0.666668740846	0.333334370426	0.099184600903
0.888891654477	0.444445827266	0.165307668199

0.777780197638   0.555557284011   0.231430735495
0.111111456797   0.888891654437   0.033061533648
0.000000000002   0.333334370426   0.099184600903
0.239938772125   0.314784993711   0.502549375815
0.496197205236   0.310725990418   0.404921936650

11. C* and CO_2 on CoGa

C Co Ga O
1.0
6.040950000000 -10.463232325983   0.000000000000
6.040950000000 10.463232325983   0.000000000000
0.000000000000   0.000000000000 24.864900000000
C Co Ga O
2 63 54   2
Selective dynamics
Direct
0.463674091912   0.261917908962   0.399398488029
0.588209465982   0.288550376873   0.511477855363
0.444445827215   0.222222913585   0.132246134551
0.333334370422   0.000000000000   0.198369201847
0.666668740844   0.000000000000   0.000000000000
0.888891654475   0.111111456840   0.066123067296
0.777780197636   0.222222913585   0.132246134551
0.666668740844   0.000000000000   0.198369201847
0.000000000002   0.333334370426   0.000000000000
0.222222913633   0.444445827266   0.066123067296
0.111111456795   0.555557284011   0.132246134551
0.000000000002   0.333334370426   0.198369201847
0.333334370424   0.333334370426   0.000000000000
0.555557284055   0.444445827266   0.066123067296
0.444445827216   0.555557284011   0.132246134551
0.333334370424   0.333334370426   0.198369201847
0.666668740846   0.333334370426   0.000000000000
0.888891654477   0.444445827266   0.066123067296
0.777780197638   0.555557284011   0.132246134551
0.666668740846   0.333334370426   0.198369201847
0.000000000004   0.666668740852   0.000000000000
0.222222913635   0.777780197692   0.066123067296
0.111111456797   0.888891654437   0.132246134551
0.000000000004   0.666668740852   0.198369201847
0.333334370426   0.666668740852   0.000000000000
0.555557284057   0.777780197692   0.066123067296
0.444445827218   0.888891654437   0.132246134551
0.666668740848   0.666668740852   0.000000000000
0.888891654479   0.777780197692   0.066123067296
0.777780197640   0.888891654437   0.132246134551
0.666668740848   0.666668740852   0.198369201847

0.555557284053	0.111111456840	0.066123067296
0.333334370422	0.000000000000	0.000000000000
0.333334370426	0.666668740852	0.198369201847
0.111111456793	0.222222913585	0.132246134551
0.000000000000	0.000000000000	0.198369201847
0.221583239456	0.110303532327	0.265452161096
0.110797381381	0.222927012828	0.333702430364
0.555638167488	0.110389402321	0.265242703285
0.451610382025	0.232761099635	0.322784011880
0.373407199467	0.084987054855	0.392772615111
0.889131001900	0.111200404401	0.263483210718
0.779199378195	0.222548412399	0.332969026765
0.694754927092	0.965452815671	0.389538379467
0.222686247769	0.443712930407	0.263956505207
0.107961553365	0.550173564716	0.324611835770
0.025741440638	0.381794232567	0.388936886664
0.556878540693	0.446428868992	0.264141485650
0.444206031388	0.552751562146	0.332795608915
0.934595824636	0.964215717031	0.390268538249
0.887858910056	0.443841656065	0.263743746238
0.338071998774	0.296353490971	0.388707082671
0.000000000000	0.000000000000	0.000000000000
0.776569809173	0.886982556075	0.323674679977
0.890294861273	0.777064478388	0.263933054007
0.272045859944	0.645562318121	0.388558147552
0.442440729777	0.887920378154	0.332881205265
0.692547005863	0.718909457422	0.387814693238
0.040732602959	0.647294079591	0.388836340484
0.774883807018	0.552848391638	0.332816656493
0.553149173649	0.776720143607	0.263935035687
0.625998303621	0.299585558483	0.390736160651
0.222222913631	0.111111456840	0.066123067296
0.224150346763	0.781635679346	0.265230821241
0.112516004354	0.888323651833	0.333159285501
0.666853542446	-0.000014484760	0.298077387941
0.444445827218	0.888891654437	0.231430735495
0.777780197640	0.888891654437	0.033061533648
0.333334370426	0.666668740852	0.099184600903
0.888891654479	0.777780197692	0.165307668199
0.777780197640	0.888891654437	0.231430735495
0.999872880450	0.000154846754	0.298588179825
0.171311392678	0.082799574804	0.381150755274
0.335525390286	0.004608035554	0.300577567530
0.576042953940	0.085177889291	0.378870819523
0.555557284057	0.777780197692	0.165307668199
0.666668740848	0.666668740852	0.099184600903

0.865823761146	0.434976145281	0.373917313374
0.998560171195	0.329683727802	0.297253290203
0.238918760761	0.434038723676	0.376342877073
0.330997524818	0.333432389210	0.296798657765
0.568947979259	0.472675904613	0.375103659793
0.670327349400	0.333811116374	0.297557230461
0.999913968314	0.666436683418	0.297926267550
0.249328742924	0.831100963210	0.383147304904
0.332586084436	0.665907160215	0.297876947302
0.517824157284	0.756528209500	0.377493785369
0.666306428562	0.665987927924	0.296955058335
0.444445827218	0.888891654437	0.033061533648
0.905078831737	0.756061593137	0.377752854450
0.905565781906	0.145280679531	0.378629235053
0.111111456797	0.888891654437	0.231430735495
0.111111456795	0.555557284011	0.033061533648
0.000000000004	0.666668740852	0.099184600903
0.111111456793	0.222222913585	0.033061533648
0.000000000000	0.000000000000	0.099184600903
0.222222913631	0.111111456840	0.165307668199
0.111111456793	0.222222913585	0.231430735495
0.444445827215	0.222222913585	0.033061533648
0.333334370422	0.000000000000	0.099184600903
0.555557284053	0.111111456840	0.165307668199
0.444445827215	0.222222913585	0.231430735495
0.777780197636	0.222222913585	0.033061533648
0.666668740844	0.000000000000	0.099184600903
0.888891654475	0.111111456840	0.165307668199
0.222222913635	0.777780197692	0.165307668199
0.777780197636	0.222222913585	0.231430735495
0.222222913633	0.444445827266	0.165307668199
0.111111456795	0.555557284011	0.231430735495
0.444445827216	0.555557284011	0.033061533648
0.333334370424	0.333334370426	0.099184600903
0.555557284055	0.444445827266	0.165307668199
0.444445827216	0.555557284011	0.231430735495
0.777780197638	0.555557284011	0.033061533648
0.666668740846	0.333334370426	0.099184600903
0.888891654477	0.444445827266	0.165307668199
0.777780197638	0.555557284011	0.231430735495
0.111111456797	0.888891654437	0.033061533648
0.000000000002	0.333334370426	0.099184600903
0.493130267637	0.242934149238	0.536417523955
0.684816191314	0.334892426331	0.486751692748

12. 2CO* on CoGa

C Co Ga O

```
1.0
  6.040950000000 -10.463232325983   0.000000000000
  6.040950000000  10.463232325983   0.000000000000
  0.000000000000   0.000000000000  24.864900000000
C Co Ga O
  2  63  54   2
Selective dynamics
Direct
  0.420497614163   0.212499980608   0.442777939097
  0.672263638601   0.333864881460   0.462444304875
  0.444445827215   0.222222913585   0.132246134551
  0.333334370422   0.000000000000   0.198369201847
  0.666668740844   0.000000000000   0.000000000000
  0.888891654475   0.111111456840   0.066123067296
  0.777780197636   0.222222913585   0.132246134551
  0.666668740844   0.000000000000   0.198369201847
  0.000000000002   0.333334370426   0.000000000000
  0.222222913633   0.444445827266   0.066123067296
  0.111111456795   0.555557284011   0.132246134551
  0.000000000002   0.333334370426   0.198369201847
  0.333334370424   0.333334370426   0.000000000000
  0.555557284055   0.444445827266   0.066123067296
  0.444445827216   0.555557284011   0.132246134551
  0.333334370424   0.333334370426   0.198369201847
  0.666668740846   0.333334370426   0.000000000000
  0.888891654477   0.444445827266   0.066123067296
  0.777780197638   0.555557284011   0.132246134551
  0.666668740846   0.333334370426   0.198369201847
  0.000000000004   0.666668740852   0.000000000000
  0.222222913635   0.777780197692   0.066123067296
  0.111111456797   0.888891654437   0.132246134551
  0.000000000004   0.666668740852   0.198369201847
  0.333334370426   0.666668740852   0.000000000000
  0.555557284057   0.777780197692   0.066123067296
  0.444445827218   0.888891654437   0.132246134551
  0.666668740848   0.666668740852   0.000000000000
  0.888891654479   0.777780197692   0.066123067296
  0.777780197640   0.888891654437   0.132246134551
  0.666668740848   0.666668740852   0.198369201847
  0.555557284053   0.111111456840   0.066123067296
  0.333334370422   0.000000000000   0.000000000000
  0.333334370426   0.666668740852   0.198369201847
  0.111111456793   0.222222913585   0.132246134551
  0.000000000000   0.000000000000   0.198369201847
  0.219337628262   0.109788924056   0.264313596519
  0.112743946944   0.224855279493   0.333187124001
```

0.557244748418	0.111609889652	0.264656917660
0.447472181289	0.224412573689	0.322835183697
0.362896089483	0.073249110677	0.391646715362
0.890167640092	0.112562418501	0.263456332716
0.778810928146	0.223551205815	0.331615199779
0.695690958570	0.970420942067	0.387523962787
0.223748428331	0.443610519372	0.264269778075
0.111848981115	0.556027133357	0.323358863938
0.031987280529	0.395167460207	0.388636811961
0.557332144308	0.446021027960	0.264570564460
0.443593807186	0.553921052205	0.333092970800
0.934063417051	0.968211354246	0.389302952434
0.884833549247	0.442547573767	0.264870873832
0.362785951659	0.292471869906	0.391048605276
0.000000000000	0.000000000000	0.000000000000
0.775832801549	0.888084619619	0.323708258936
0.890263544304	0.777858973219	0.263508203613
0.272148715428	0.637481005766	0.388838236487
0.443483891124	0.890431720855	0.333155568353
0.695074751462	0.725892961419	0.387610799087
0.033059585567	0.638650447575	0.388511210522
0.779031987821	0.556287521729	0.331640442908
0.553270014179	0.776774798920	0.263879406376
0.578778727127	0.289922771335	0.403924663480
0.222222913631	0.111111456840	0.066123067296
0.223883805796	0.780669343897	0.264375253296
0.112861903118	0.888859817340	0.333222111855
0.666189190741	0.000544756727	0.297307369112
0.444445827218	0.888891654437	0.231430735495
0.777780197640	0.888891654437	0.033061533648
0.333334370426	0.666668740852	0.099184600903
0.888891654479	0.777780197692	0.165307668199
0.777780197640	0.888891654437	0.231430735495
0.998955961870	-0.000228537598	0.298126347042
0.174823532708	0.088101933394	0.376998015646
0.333527279250	0.001100466286	0.298529654174
0.572885458641	0.095731944868	0.374950720022
0.555557284057	0.777780197692	0.165307668199
0.666668740848	0.666668740852	0.099184600903
0.853320050599	0.427432924998	0.377514749143
1.000198459106	0.332859810698	0.297768309874
0.243632418199	0.425547407967	0.380002002902
0.333350729258	0.333151049450	0.298259186247
0.573400339197	0.478797337405	0.375070038990
0.665766648015	0.333253099572	0.302028528738
1.000391045542	0.667774287579	0.297792653620

0.244351485549 0.821178104402 0.380777003198
0.334441519950 0.667469493505 0.298136924289
0.516774696961 0.758992478516 0.378577540124
0.666229850829 0.666168433681 0.297266426692
0.444445827218 0.888891654437 0.033061533648
0.906980142849 0.758280346167 0.378875190963
0.907202537216 0.151083285394 0.379366980611
0.111111456797 0.888891654437 0.231430735495
0.111111456795 0.555557284011 0.033061533648
0.000000000004 0.666668740852 0.099184600903
0.111111456793 0.222222913585 0.033061533648
0.000000000000 0.000000000000 0.099184600903
0.222222913631 0.111111456840 0.165307668199
0.111111456793 0.222222913585 0.231430735495
0.444445827215 0.222222913585 0.033061533648
0.333334370422 0.000000000000 0.099184600903
0.555557284053 0.111111456840 0.165307668199
0.444445827215 0.222222913585 0.231430735495
0.777780197636 0.222222913585 0.033061533648
0.666668740844 0.000000000000 0.099184600903
0.888891654475 0.111111456840 0.165307668199
0.222222913635 0.777780197692 0.165307668199
0.777780197636 0.222222913585 0.231430735495
0.222222913633 0.444445827266 0.165307668199
0.111111456795 0.555557284011 0.231430735495
0.444445827216 0.555557284011 0.033061533648
0.333334370424 0.333334370426 0.099184600903
0.555557284055 0.444445827266 0.165307668199
0.444445827216 0.555557284011 0.231430735495
0.777780197638 0.555557284011 0.033061533648
0.666668740846 0.333334370426 0.099184600903
0.888891654477 0.444445827266 0.165307668199
0.777780197638 0.555557284011 0.231430735495
0.111111456797 0.888891654437 0.033061533648
0.000000000002 0.333334370426 0.099184600903
0.387226279138 0.198365700877 0.489495487167
0.726602048032 0.357917285490 0.503453834454

13. C-C-C* and CO_2 on CoGa

C Co Ga O
1.00000000000000
12.0818999999999992 0.0000000000000000 0.0000000000000000
-6.0409499999999996 10.4632323259831992 0.0000000000000000
0.0000000000000015 0.0000000000000026 24.8648999999999987
C Co Ga O
3 63 54 1
Selective dynamics

Direct

0.4510738879401358	0.2385036133605823	0.4062213446409965
0.3288836760279653	0.1720286820286905	0.4259321487915212
0.2137265985735579	0.1629560518501962	0.4331997769524302
0.2220834468705062	0.1103188473423589	0.2634105999411660
0.1161920516138617	0.2219350473528224	0.3308313968049855
0.8089981700467689	0.9090617865990892	0.4166594724077905
0.5536158706856581	0.1126361581702201	0.2645711226350319
0.4453161173236558	0.2256463409230677	0.3294267107762294
0.2127652087239187	0.0931487784833484	0.3612500551393218
0.8907903346782592	0.1098500583976792	0.2646832514253721
0.7768628265949867	0.2221158191177722	0.3310207343354171
0.6732454274681863	0.9891438581254032	0.3866579843590282
0.2222725248845425	0.4439752222053700	0.2632266786801127
0.1097605115984410	0.5523115437539906	0.3321748124385782
0.0081691475155898	0.3418809724521027	0.3870013358349734
0.5564988889598692	0.4437697082288944	0.2641262756102385
0.4421726566286344	0.5510929201223786	0.3329599898104835
0.3314907537115370	0.3180582346834984	0.3896950447984950
0.8853569745047446	0.4422225896301989	0.2652526355149883
0.7788544916340367	0.5610404056681119	0.3318567247110465
0.6235374018902519	0.3035010905541586	0.4001928121373475
0.2242590868844328	0.7804931860671372	0.2637947438577750
0.1119753593475684	0.8882641869055564	0.3286828321355817
0.0176707206375225	0.6765867057838701	0.3865248685642558
0.5544158309661815	0.7765557765499813	0.2631314814820693
0.4443345745786873	0.8860608207000482	0.3306693120070459
0.3201045315072056	0.6678632116175091	0.3880262397217990
0.8898147930300633	0.7805818522422614	0.2646229780208735
0.7706058292335586	0.8854903037315011	0.3267373711061678
0.6848958830896598	0.6913227022590667	0.3867786647030594
0.0000000000000000	0.0000000000000000	0.0000000000000000
0.2222229136311569	0.1111114568404403	0.0661230672956634
0.1111114567926563	0.2222229135853127	0.1322461345511172
0.0000000000000000	0.0000000000000000	0.1983692018467806
0.3333343704218734	0.0000000000000000	0.0000000000000000
0.5555572840530303	0.1111114568404403	0.0661230672956634
0.4444458272145226	0.2222229135853127	0.1322461345511172
0.3333343704218734	0.0000000000000000	0.1983692018467806
0.6666687408437397	0.0000000000000000	0.0000000000000000
0.8888916544748966	0.1111114568404403	0.0661230672956634
0.7777801976363961	0.2222229135853127	0.1322461345511172
0.6666687408437397	0.0000000000000000	0.1983692018467806
0.0000000000019398	0.3333343704257530	0.0000000000000000
0.2222229136330967	0.4444458272661933	0.0661230672956634
0.1111114567945961	0.5555572840110656	0.1322461345511172

0.0000000000019398	0.3333343704257530	0.1983692018467806
0.3333343704238132	0.3333343704257530	0.0000000000000000
0.5555572840549701	0.4444458272661933	0.0661230672956634
0.4444458272164695	0.5555572840110656	0.1322461345511172
0.3333343704238132	0.3333343704257530	0.1983692018467806
0.6666687408456795	0.3333343704257530	0.0000000000000000
0.8888916544768364	0.4444458272661933	0.0661230672956634
0.7777801976383358	0.5555572840110656	0.1322461345511172
0.6666687408456795	0.3333343704257530	0.1983692018467806
0.0000000000038796	0.6666687408515060	0.0000000000000000
0.2222229136350364	0.7777801976919463	0.0661230672956634
0.1111114567965359	0.8888916544368186	0.1322461345511172
0.0000000000038796	0.6666687408515060	0.1983692018467806
0.3333343704257530	0.6666687408515060	0.0000000000000000
0.5555572840569099	0.7777801976919463	0.0661230672956634
0.4444458272184093	0.8888916544368186	0.1322461345511172
0.3333343704257530	0.6666687408515060	0.1983692018467806
0.6666687408476264	0.6666687408515060	0.0000000000000000
0.8888916544787833	0.7777801976919463	0.0661230672956634
0.7777801976402756	0.8888916544368186	0.1322461345511172
0.6666687408476264	0.6666687408515060	0.1983692018467806
0.1111114567926563	0.2222229135853127	0.0330615336478317
0.0000000000000000	0.0000000000000000	0.0991846009032784
0.2222229136311569	0.1111114568404403	0.1653076681989489
0.1111114567926563	0.2222229135853127	0.2314307354946123
0.4444458272145226	0.2222229135853127	0.0330615336478317
0.3333343704218734	0.0000000000000000	0.0991846009032784
0.5555572840530303	0.1111114568404403	0.1653076681989489
0.4444458272145226	0.2222229135853127	0.2314307354946123
0.7777801976363961	0.2222229135853127	0.0330615336478317
0.6666687408437397	0.0000000000000000	0.0991846009032784
0.8888916544748966	0.1111114568404403	0.1653076681989489
0.7777801976363961	0.2222229135853127	0.2314307354946123
0.1111114567945961	0.5555572840110656	0.0330615336478317
0.0000000000019398	0.3333343704257530	0.0991846009032784
0.2222229136330967	0.4444458272661933	0.1653076681989489
0.1111114567945961	0.5555572840110656	0.2314307354946123
0.4444458272164695	0.5555572840110656	0.0330615336478317
0.3333343704238132	0.3333343704257530	0.0991846009032784
0.5555572840549701	0.4444458272661933	0.1653076681989489
0.4444458272164695	0.5555572840110656	0.2314307354946123
0.7777801976383358	0.5555572840110656	0.0330615336478317
0.6666687408456795	0.3333343704257530	0.0991846009032784
0.8888916544768364	0.4444458272661933	0.1653076681989489
0.7777801976383358	0.5555572840110656	0.2314307354946123
0.1111114567965359	0.8888916544368186	0.0330615336478317

0.0000000000038796 0.6666687408515060 0.0991846009032784
0.2222229136350364 0.7777801976919463 0.1653076681989489
0.1111114567965359 0.8888916544368186 0.2314307354946123
0.4444458272184093 0.8888916544368186 0.0330615336478317
0.3333343704257530 0.6666687408515060 0.0991846009032784
0.5555572840569099 0.7777801976919463 0.1653076681989489
0.4444458272184093 0.8888916544368186 0.2314307354946123
0.7777801976402756 0.8888916544368186 0.0330615336478317
0.6666687408476264 0.6666687408515060 0.0991846009032784
0.8888916544787833 0.7777801976919463 0.1653076681989489
0.7777801976402756 0.8888916544368186 0.2314307354946123
0.0014304612600898 0.0009111362494300 0.3021676141076428
0.0281516872571500 0.0393915904022298 0.4288223260987488
0.3347540262820646 0.0035367732485971 0.3030145724778481
0.5516637567145550 0.0938730860516249 0.3686520456395699
0.6657276983916823 0.0008953069619172 0.2946617410677837
0.8943105463670626 0.1184732542526919 0.3693227564283600
0.0004189672705266 0.3334853067822326 0.2958810099098316
0.2289633376963309 0.4449500744276678 0.3729232994120678
0.3337114984874225 0.3335971239937867 0.2959158520461547
0.5631473067884830 0.4631689737522138 0.3734751172251127
0.6683099974700243 0.3364973768293668 0.3013066197886728
0.8876431120831518 0.4495555079813846 0.3772660221212925
0.9996245463553910 0.6637427429889476 0.2954951926012216
0.2358851774877850 0.8012041665000550 0.3718573031698813
0.3315220983315817 0.6639922357191024 0.2963337646824568
0.5424286348460772 0.7678874248382207 0.3739168823731989
0.6642778931580621 0.6650587671966754 0.2951101939360184
0.9061774309772248 0.7894659520056136 0.3750383378769210
0.7642196416076856 0.3703559068397260 0.4353883337955914

14. C-C* and 2CO* on CoGa

C Co Ga O
1.00000000000000
12.0818999999999992 0.0000000000000000 0.0000000000000000
-6.0409499999999996 10.4632323259831992 0.0000000000000000
0.0000000000000015 0.0000000000000026 24.8648999999999987
C Co Ga O
3 63 54 1
Selective dynamics
Direct
0.6528495353091299 0.3488040313360959 0.4603165094817600
0.3468105800758990 0.1784937069693400 0.4050164545449118
0.2406240108460946 0.1598199653361840 0.4314791137707251
0.2185113080523028 0.1082492439234855 0.2608686675014906
0.1132709278531297 0.2239966682644579 0.3301874879392679
0.8206772944919243 0.9116754939846412 0.4166796920788798

0.5526089617463263	0.1123088209632383	0.2653630533875486
0.4499565199998007	0.2220296395095751	0.3450284074760758
0.2113369541947188	0.0982504721634169	0.3552514855293313
0.8906551768713346	0.1105024374466748	0.2646862114906114
0.7773720763335813	0.2247685757399422	0.3316711170791232
0.6744162348860245	0.9923685376687252	0.3874080529269018
0.2235978676439491	0.4441924994707565	0.2633468102056439
0.1137056957023222	0.5574885624288183	0.3313479831424704
0.0018131442965947	0.3372843401721257	0.3868788155124761
0.5552042713256889	0.4426631832482422	0.2641189938614467
0.4429597579416045	0.5475888090862147	0.3341480334323164
0.3260116990975023	0.3229969802219899	0.3887302615510273
0.8876858748369882	0.4437618071525435	0.2637151354066478
0.7787884447629966	0.5591401594476730	0.3320545733048394
0.6722582846569310	0.3306469204713767	0.3921235436295041
0.2254651656685908	0.7813251762430723	0.2637876258863616
0.1116053649133521	0.8888367107214769	0.3278448193673600
0.0139121100946407	0.6759406640662533	0.3864167533571445
0.5556985733413620	0.7774659968805181	0.2627763736480858
0.4463373572545151	0.8906499708990206	0.3318035991649083
0.3234309488313851	0.6708054698227558	0.3876377437665393
0.8888113219337145	0.7796047835380139	0.2648319695126728
0.7690319337226028	0.8840592340399769	0.3271856422111767
0.6827768736406866	0.6882594323606083	0.3874100875050394
0.0000000000000000	0.0000000000000000	0.0000000000000000
0.2222229136311569	0.1111114568404403	0.0661230672956634
0.1111114567926563	0.2222229135853127	0.1322461345511172
0.0000000000000000	0.0000000000000000	0.1983692018467806
0.3333343704218734	0.0000000000000000	0.0000000000000000
0.5555572840530303	0.1111114568404403	0.0661230672956634
0.4444458272145226	0.2222229135853127	0.1322461345511172
0.3333343704218734	0.0000000000000000	0.1983692018467806
0.6666687408437397	0.0000000000000000	0.0000000000000000
0.8888916544748966	0.1111114568404403	0.0661230672956634
0.7777801976363961	0.2222229135853127	0.1322461345511172
0.6666687408437397	0.0000000000000000	0.1983692018467806
0.0000000000019398	0.3333343704257530	0.0000000000000000
0.2222229136330967	0.4444458272661933	0.0661230672956634
0.1111114567945961	0.5555572840110656	0.1322461345511172
0.0000000000019398	0.3333343704257530	0.1983692018467806
0.3333343704238132	0.3333343704257530	0.0000000000000000
0.5555572840549701	0.4444458272661933	0.0661230672956634
0.4444458272164695	0.5555572840110656	0.1322461345511172
0.3333343704238132	0.3333343704257530	0.1983692018467806
0.6666687408456795	0.3333343704257530	0.0000000000000000
0.8888916544768364	0.4444458272661933	0.0661230672956634

0.7777801976383358	0.5555572840110656	0.1322461345511172
0.6666687408456795	0.3333343704257530	0.1983692018467806
0.0000000000038796	0.6666687408515060	0.0000000000000000
0.2222229136350364	0.7777801976919463	0.0661230672956634
0.1111114567965359	0.8888916544368186	0.1322461345511172
0.0000000000038796	0.6666687408515060	0.1983692018467806
0.3333343704257530	0.6666687408515060	0.0000000000000000
0.5555572840569099	0.7777801976919463	0.0661230672956634
0.4444458272184093	0.8888916544368186	0.1322461345511172
0.3333343704257530	0.6666687408515060	0.1983692018467806
0.6666687408476264	0.6666687408515060	0.0000000000000000
0.8888916544787833	0.7777801976919463	0.0661230672956634
0.7777801976402756	0.8888916544368186	0.1322461345511172
0.6666687408476264	0.6666687408515060	0.1983692018467806
0.1111114567926563	0.2222229135853127	0.0330615336478317
0.0000000000000000	0.0000000000000000	0.0991846009032784
0.2222229136311569	0.1111114568404403	0.1653076681989489
0.1111114567926563	0.2222229135853127	0.2314307354946123
0.4444458272145226	0.2222229135853127	0.0330615336478317
0.3333343704218734	0.0000000000000000	0.0991846009032784
0.5555572840530303	0.1111114568404403	0.1653076681989489
0.4444458272145226	0.2222229135853127	0.2314307354946123
0.7777801976363961	0.2222229135853127	0.0330615336478317
0.6666687408437397	0.0000000000000000	0.0991846009032784
0.8888916544748966	0.1111114568404403	0.1653076681989489
0.7777801976363961	0.2222229135853127	0.2314307354946123
0.1111114567945961	0.5555572840110656	0.0330615336478317
0.0000000000019398	0.3333343704257530	0.0991846009032784
0.2222229136330967	0.4444458272661933	0.1653076681989489
0.1111114567945961	0.5555572840110656	0.2314307354946123
0.4444458272164695	0.5555572840110656	0.0330615336478317
0.3333343704238132	0.3333343704257530	0.0991846009032784
0.5555572840549701	0.4444458272661933	0.1653076681989489
0.4444458272164695	0.5555572840110656	0.2314307354946123
0.7777801976383358	0.5555572840110656	0.0330615336478317
0.6666687408456795	0.3333343704257530	0.0991846009032784
0.8888916544768364	0.4444458272661933	0.1653076681989489
0.7777801976383358	0.5555572840110656	0.2314307354946123
0.1111114567965359	0.8888916544368186	0.0330615336478317
0.0000000000038796	0.6666687408515060	0.0991846009032784
0.2222229136350364	0.7777801976919463	0.1653076681989489
0.1111114567965359	0.8888916544368186	0.2314307354946123
0.4444458272184093	0.8888916544368186	0.0330615336478317
0.3333343704257530	0.6666687408515060	0.0991846009032784
0.5555572840569099	0.7777801976919463	0.1653076681989489
0.4444458272184093	0.8888916544368186	0.2314307354946123

0.7777801976402756 0.8888916544368186 0.0330615336478317
0.6666687408476264 0.6666687408515060 0.0991846009032784
0.8888916544787833 0.7777801976919463 0.1653076681989489
0.7777801976402756 0.8888916544368186 0.2314307354946123
0.9987672428272910 0.0006488806333979 0.3015516669237256
0.0475950734454547 0.0432537334360821 0.4271647136782276
0.3368694293674054 0.0073029697673873 0.3035203092731374
0.5572961767124225 0.1038592272221371 0.3741916542949737
0.6661847338036401 0.0024938148757747 0.2954438255876569
0.8949657936020206 0.1159070767697285 0.3700691165372140
0.0005768835026859 0.3355038059933386 0.2956474450807812
0.2237208663073676 0.4471004140439204 0.3721959535448303
0.3356732295027769 0.3296265385038371 0.2962476369908846
0.5646119981469758 0.4532560421035947 0.3754505968905353
0.6664044899166005 0.3351596747047531 0.2971525594386071
0.8922618169796016 0.4504562798930852 0.3730950906439126
0.9995248620212670 0.6640631104280739 0.2951855159916811
0.2327245716667879 0.7980888229029708 0.3705234065710640
0.3352910121612868 0.6657609217106576 0.2961582929095185
0.5453535407769393 0.7706253047494157 0.3737234470909778
0.6631905976186659 0.6631564720437666 0.2957541801943874
0.9039595171881298 0.7883992076854516 0.3728530815513359
0.6397777686718396 0.3607053820280373 0.5061851159330337

C. $CoGa_3$(111)

1. CH_4 on $CoGa_3$

C Co Ga H
1.0
17.550000000000 0.000000000000 0.000000000000
-8.774985327665 15.455371163103 0.000000000000
0.000000000000 0.000000000000 21.997800000000
C Co Ga H
1 32 96 4
Selective dynamics
Direct
0.472724517264 0.689051018022 0.403169859792
0.602222909280 0.954447898101 0.153925148651
0.821302877904 0.892608699359 0.090931261035
0.538804210476 0.673528328899 0.122428204866
0.884721541188 0.673528328899 0.122428204866
0.102223876630 0.954447898101 0.153925148651
0.038805177827 0.673528328899 0.122428204866
0.384722508596 0.673528328899 0.122428204866
0.602222873760 0.454447958440 0.153925148651
0.821302842352 0.392608759632 0.090931261035
0.538804174957 0.173528389237 0.122428204866

0.884721505669	0.173528389237	0.122428204866
0.102223841111	0.454447958440	0.153925148651
0.321303809760	0.392608759632	0.090931261035
0.038805142307	0.173528389237	0.122428204866
0.384722473076	0.173528389237	0.122428204866
0.321303845255	0.892608699359	0.090931261035
0.652068698333	0.554506176407	0.252234396596
0.930037171346	0.610525380648	0.313698507685
0.223434386229	0.335792574587	0.278569192318
0.362118683195	0.335946667482	0.278528808728
0.152007988503	0.054289331451	0.252285515417
0.724183903718	0.335984264578	0.278827822612
0.861485014852	0.336081803194	0.278764664041
0.652026954286	0.054387985586	0.252394541701
0.430120778064	0.110639951462	0.313604774129
0.223979607316	0.835917479316	0.278884928128
0.361287239617	0.836391286404	0.278632379369
0.152162094345	0.554485923764	0.252266636692
0.431006906903	0.612189196501	0.315668745644
0.724693583320	0.836497672140	0.278537634756
0.930299530986	0.110693202449	0.313611180488
0.861504299887	0.835943418308	0.278898730184
0.357028963080	0.465427876810	0.307939086264
0.439670848716	0.129341631019	0.212869391880
0.225587472304	0.201326191815	0.281447396867
0.610495772197	0.248466463150	0.217043850407
0.787991770121	0.085853801155	0.300223277984
0.887911558085	0.248722654012	0.216950461629
0.307767172466	0.366047681957	0.193547784202
0.667077973169	0.425567103103	0.251210097655
0.547688241060	0.085959711389	0.300174469231
0.008235920174	0.425536567442	0.251243570226
0.592659691446	0.587353521905	0.164186825228
0.387342638597	0.247769876919	0.217055527087
0.287837402579	0.086075900986	0.300268610960
0.047942935460	0.086154223929	0.300198063829
0.109888701784	0.247642450953	0.217207073541
0.744691397315	0.587353521905	0.164186825228
0.914102457676	0.926176209160	0.000000000000
0.762070751807	0.926176209160	0.000000000000
0.895640134309	0.541283592139	0.112839694288
0.614060280228	0.978122679583	0.048499474675
0.508589400972	0.424742218999	0.251229576668
0.165996923721	0.424876995266	0.251315534670
0.809230636457	0.368791368654	0.193995309703
0.787887389348	0.586117953873	0.300305010677

0.939241246449	0.129290589068	0.212873095204
0.725442848074	0.701305454363	0.281218961937
0.939370161634	0.629232163460	0.212903304618
0.857384980139	0.964625728721	0.312951107374
0.810089030195	0.869452999893	0.193979865709
0.508330492425	0.925473191743	0.251562265985
0.666696241378	0.925471310987	0.251451908904
0.887874817814	0.748515846961	0.217093741922
0.527885617323	0.805773065595	0.132016715444
0.546863373023	0.585712208957	0.297486956187
0.611722337468	0.749711757624	0.216157800517
0.225454280840	0.701219317025	0.281179689850
0.440015552958	0.630815969294	0.213520940504
0.356894513758	0.964583339439	0.312804109021
0.308914944259	0.869032007112	0.193943323108
0.008286354448	0.925522819769	0.251458337547
0.166747543268	0.925422732673	0.251407492330
0.387686316842	0.749738458305	0.216308835722
0.288278792045	0.585855897945	0.298621865336
0.047608643675	0.585869817070	0.300204683099
0.110196052645	0.748253056388	0.217153441570
0.725510427657	0.201089724641	0.281172477607
0.856845006689	0.464419232767	0.312938953079
0.978876531798	0.707756665470	0.032170109784
0.178835321757	0.759703135893	0.080669584458
0.830866060219	0.759703135893	0.080669584458
0.978876496246	0.207756725744	0.032170109784
0.678834318798	0.259703196167	0.080669584458
0.830866024667	0.259703196167	0.080669584458
0.546468836393	0.092939763002	0.040334792252
0.961762374381	0.423528359036	0.122428204866
0.711762858028	0.423528359036	0.122428204866
0.796468352746	0.092939763002	0.040334792252
0.244692329146	0.087353582244	0.164186825228
0.414103389507	0.426176269498	0.000000000000
0.092660623334	0.087353582244	0.164186825228
0.262071683695	0.426176269498	0.000000000000
0.395641066197	0.041283652477	0.112839694288
0.114061212059	0.478122739921	0.048499474675
0.027886549154	0.305773125933	0.132016715444
0.478877463654	0.207756725744	0.032170109784
0.178835286206	0.259703196167	0.080669584458
0.330866992018	0.259703196167	0.080669584458
0.046469803800	0.092939763002	0.040334792252
0.461763341732	0.423528359036	0.122428204866
0.211763825435	0.423528359036	0.122428204866

0.296469320097   0.092939763002   0.040334792252
0.678834354350   0.759703135893   0.080669584458
0.614060244708   0.478122739921   0.048499474675
0.527885581804   0.305773125933   0.132016715444
0.762070716287   0.426176269498   0.000000000000
0.546468871912   0.592939702663   0.040334792252
0.961762409844   0.923528298698   0.122428204866
0.711762893547   0.923528298698   0.122428204866
0.796468388209   0.592939702663   0.040334792252
0.244692364665   0.587353521905   0.164186825228
0.414103425026   0.926176209160   0.000000000000
0.092660658853   0.587353521905   0.164186825228
0.262071719214   0.926176209160   0.000000000000
0.395641101716   0.541283592139   0.112839694288
0.895640098790   0.041283652477   0.112839694288
0.114061247578   0.978122679583   0.048499474675
0.478877499206   0.707756665470   0.032170109784
0.330867027569   0.759703135893   0.080669584458
0.046469839320   0.592939702663   0.040334792252
0.461763377251   0.923528298698   0.122428204866
0.211763860955   0.923528298698   0.122428204866
0.296469355616   0.592939702663   0.040334792252
0.744691361795   0.087353582244   0.164186825228
0.914102422156   0.426176269498   0.000000000000
0.592659655926   0.087353582244   0.164186825228
0.027886584674   0.805773065595   0.132016715444
0.487207574418   0.724970643645   0.359250763262
0.431180301779   0.706119283409   0.430221831930
0.534922045055   0.707487035302   0.425944638451
0.436368086814   0.615935641565   0.399140577901

2. CH_3* and H* on $CoGa_3$

C Co Ga H
1.0
17.550000000000   0.000000000000   0.000000000000
-8.774985327665 15.455371163103   0.000000000000
0.000000000000   0.000000000000 21.997800000000
C Co Ga H
1 32 96   4
Selective dynamics
Direct
0.383556486942   0.653488328584   0.384535111508
0.602222909280   0.954447898101   0.153925148651
0.821302877904   0.892608699359   0.090931261035
0.538804210476   0.673528328899   0.122428204866
0.884721541188   0.673528328899   0.122428204866
0.102223876630   0.954447898101   0.153925148651

0.038805177827	0.673528328899	0.122428204866
0.384722508596	0.673528328899	0.122428204866
0.602222873760	0.454447958440	0.153925148651
0.821302842352	0.392608759632	0.090931261035
0.538804174957	0.173528389237	0.122428204866
0.884721505669	0.173528389237	0.122428204866
0.102223841111	0.454447958440	0.153925148651
0.321303809760	0.392608759632	0.090931261035
0.038805142307	0.173528389237	0.122428204866
0.384722473076	0.173528389237	0.122428204866
0.321303845255	0.892608699359	0.090931261035
0.651228828935	0.554916726497	0.252743017066
0.930237365888	0.610800332494	0.313425262534
0.221837617399	0.334138627462	0.278459793842
0.362216914033	0.334235828165	0.278675714409
0.151669214251	0.053827658887	0.252248313876
0.724149175164	0.336432742569	0.278664380155
0.862028421306	0.336484097060	0.278731483776
0.652112267545	0.054634221316	0.252378471401
0.430078568574	0.110632227182	0.313581878734
0.223889358008	0.836049502049	0.278978969229
0.361304622782	0.836546870207	0.278212117909
0.153800514317	0.555111108482	0.252933082501
0.428747238440	0.607979602760	0.316876143672
0.724126006498	0.836255867716	0.278666805917
0.930086087597	0.110511563924	0.313547479130
0.861223979290	0.835813130993	0.279013755423
0.355159960843	0.461691358543	0.306031222707
0.439895261478	0.128425224359	0.212811121003
0.225279334716	0.200721893080	0.281468654038
0.610437938640	0.248518027500	0.217073957130
0.787891244403	0.086176650927	0.300435977501
0.887890685310	0.248794279659	0.216929535278
0.306503041465	0.362757743656	0.193348864202
0.666835401426	0.425929495439	0.251243705948
0.547862125081	0.086201212272	0.300384585767
0.008965119911	0.425931106614	0.251399634454
0.592659691446	0.587353521905	0.164186825228
0.387526288826	0.246617164172	0.217200460653
0.287825525369	0.086272560129	0.300271074070
0.047961791194	0.086278328327	0.300160279742
0.109137752468	0.246467083771	0.216949775282
0.744691397315	0.587353521905	0.164186825228
0.914102457676	0.926176209160	0.000000000000
0.762070751807	0.926176209160	0.000000000000
0.895640134309	0.541283592139	0.112839694288

0.614060280228	0.978122679583	0.048499474675
0.508247155857	0.424622739032	0.251642450033
0.165647544867	0.424153353144	0.251607244950
0.809285992252	0.368719158267	0.193944638885
0.787729233757	0.586472536034	0.300231098199
0.938508234344	0.128834716991	0.212807267564
0.724836050867	0.701294590951	0.282523456061
0.939482345500	0.629455414976	0.212786553177
0.857126324233	0.964470940967	0.312694312303
0.809796524546	0.869133466465	0.194063015503
0.508396839033	0.925730357423	0.251579250346
0.666820370348	0.925809306488	0.251550772483
0.887376462042	0.748493995722	0.216967798073
0.527885617323	0.805773065595	0.132016715444
0.546231295885	0.585848792426	0.296690635434
0.612225301721	0.750513444284	0.215404146555
0.225506111457	0.701565076986	0.281513947801
0.440037197246	0.629987848784	0.213699835173
0.356770301664	0.964569784729	0.312840674050
0.308731033566	0.869628618252	0.193956933681
0.008039760769	0.925402326581	0.251582155835
0.166516541352	0.925350467819	0.251492572681
0.387765291997	0.750670629643	0.215102037761
0.290826340097	0.586307231597	0.296507688277
0.048222518929	0.586128619286	0.300467407091
0.110239841321	0.748418029985	0.217156011120
0.725704677370	0.201573939142	0.281199674517
0.857067067336	0.464712610702	0.312190844795
0.978876531798	0.707756665470	0.032170109784
0.178835321757	0.759703135893	0.080669584458
0.830866060219	0.759703135893	0.080669584458
0.978876496246	0.207756725744	0.032170109784
0.678834318798	0.259703196167	0.080669584458
0.830866024667	0.259703196167	0.080669584458
0.546468836393	0.092939763002	0.040334792252
0.961762374381	0.423528359036	0.122428204866
0.711762858028	0.423528359036	0.122428204866
0.796468352746	0.092939763002	0.040334792252
0.244692329146	0.087353582244	0.164186825228
0.414103389507	0.426176269498	0.000000000000
0.092660623334	0.087353582244	0.164186825228
0.262071683695	0.426176269498	0.000000000000
0.395641066197	0.041283652477	0.112839694288
0.114061212059	0.478122739921	0.048499474675
0.027886549154	0.305773125933	0.132016715444
0.478877463654	0.207756725744	0.032170109784

0.178835286206 0.259703196167 0.080669584458
0.330866992018 0.259703196167 0.080669584458
0.046469803800 0.092939763002 0.040334792252
0.461763341732 0.423528359036 0.122428204866
0.211763825435 0.423528359036 0.122428204866
0.296469320097 0.092939763002 0.040334792252
0.678834354350 0.759703135893 0.080669584458
0.614060244708 0.478122739921 0.048499474675
0.527885581804 0.305773125933 0.132016715444
0.762070716287 0.426176269498 0.000000000000
0.546468871912 0.592939702663 0.040334792252
0.961762409844 0.923528298698 0.122428204866
0.711762893547 0.923528298698 0.122428204866
0.796468388209 0.592939702663 0.040334792252
0.244692364665 0.587353521905 0.164186825228
0.414103425026 0.926176209160 0.000000000000
0.092660658853 0.587353521905 0.164186825228
0.262071719214 0.926176209160 0.000000000000
0.395641101716 0.541283592139 0.112839694288
0.895640098790 0.041283652477 0.112839694288
0.114061247578 0.978122679583 0.048499474675
0.478877499206 0.707756665470 0.032170109784
0.330867027569 0.759703135893 0.080669584458
0.046469839320 0.592939702663 0.040334792252
0.461763377251 0.923528298698 0.122428204866
0.211763860955 0.923528298698 0.122428204866
0.296469355616 0.592939702663 0.040334792252
0.744691361795 0.087353582244 0.164186825228
0.914102422156 0.426176269498 0.000000000000
0.592659655926 0.087353582244 0.164186825228
0.027886584674 0.805773065595 0.132016715444
0.442493558349 0.718363842918 0.382065243549
0.327438126733 0.665727023203 0.386263580663
0.382643429245 0.621089003414 0.427154393464
0.495667871781 0.608011606421 0.364841805934

3. CH_3* on $CoGa_3$

C Co Ga H
1.0
17.550000000000 0.000000000000 0.000000000000
-8.774985327665 15.455371163103 0.000000000000
0.000000000000 0.000000000000 21.997800000000
C Co Ga H
1 32 96 3
Selective dynamics
Direct
0.449306409840 0.644441427587 0.403253073697

0.602222909280	0.954447898101	0.153925148651
0.821302877904	0.892608699359	0.090931261035
0.538804210476	0.673528328899	0.122428204866
0.884721541188	0.673528328899	0.122428204866
0.102223876630	0.954447898101	0.153925148651
0.038805177827	0.673528328899	0.122428204866
0.384722508596	0.673528328899	0.122428204866
0.602222873760	0.454447958440	0.153925148651
0.821302842352	0.392608759632	0.090931261035
0.538804174957	0.173528389237	0.122428204866
0.884721505669	0.173528389237	0.122428204866
0.102223841111	0.454447958440	0.153925148651
0.321303809760	0.392608759632	0.090931261035
0.038805142307	0.173528389237	0.122428204866
0.384722473076	0.173528389237	0.122428204866
0.321303845255	0.892608699359	0.090931261035
0.651971222291	0.554417975492	0.252257563509
0.930094239424	0.610419480665	0.313714214764
0.223773470916	0.335856925752	0.278870060353
0.361753209926	0.335986568530	0.278831205588
0.152082924333	0.054369796027	0.252320270417
0.724084018142	0.335818708089	0.278877202950
0.861403497830	0.335907390753	0.278846575241
0.652094693218	0.054435723867	0.252321235376
0.430192262857	0.110270845280	0.313609704204
0.224093627183	0.835773004301	0.278892546450
0.361459830960	0.835786580512	0.278570710219
0.152132277023	0.554338025016	0.252202896392
0.429803511951	0.609780908522	0.318579371619
0.723925412840	0.835759365558	0.278622929789
0.929970221880	0.110265677260	0.313622939761
0.861362326502	0.835856522794	0.278868174382
0.356878992804	0.464450441167	0.311691157213
0.439527848823	0.129413049327	0.212934530121
0.225607115679	0.201292236559	0.281327420644
0.610245899571	0.248224343601	0.217176628192
0.787715501512	0.085469610670	0.300403617933
0.887892905065	0.248563173052	0.217075753957
0.308561994861	0.367437318060	0.193899844594
0.667033818588	0.425480930936	0.251425181396
0.547711202976	0.085645138483	0.300404742944
0.008168135993	0.425507775846	0.251492908379
0.592659691446	0.587353521905	0.164186825228
0.387483115491	0.248137595285	0.217051932844
0.288133175714	0.086174050999	0.300232616153
0.047884442229	0.086157708362	0.300223641408

0.110202591058	0.247752337300	0.217161341947
0.744691397315	0.587353521905	0.164186825228
0.914102457676	0.926176209160	0.000000000000
0.762070751807	0.926176209160	0.000000000000
0.895640134309	0.541283592139	0.112839694288
0.614060280228	0.978122679583	0.048499474675
0.508459133172	0.423875533493	0.251143466551
0.164975321338	0.423676785944	0.250986650771
0.808999115222	0.368381805761	0.193974769206
0.787806481144	0.586046268875	0.300331555007
0.939443840298	0.129433050643	0.212933714481
0.724960963799	0.700976835197	0.281742773653
0.939566468510	0.629574792490	0.212958704251
0.857001811133	0.964188622888	0.312878856440
0.809621088963	0.869124320340	0.194017621166
0.508358375521	0.925509097005	0.251356180170
0.666682308924	0.925336526508	0.251281940885
0.887704309569	0.748709346026	0.216817839186
0.527885617323	0.805773065595	0.132016715444
0.545451597284	0.584648578102	0.297732224387
0.611320433625	0.749103161030	0.215944006885
0.225970973027	0.700914041031	0.281570881129
0.438578650747	0.627836565244	0.214005753812
0.356842306226	0.964167210079	0.312900829013
0.309178598692	0.869176077301	0.194037719415
0.008330651225	0.925498058163	0.251589832998
0.166878026515	0.925468658753	0.251622769348
0.387758196066	0.749502895142	0.215771332032
0.288475395178	0.584185609686	0.298199653117
0.047975559422	0.586069761489	0.300341385070
0.110568881204	0.748443200606	0.216959607236
0.725491274045	0.200973405590	0.281503938506
0.856923324543	0.464292998286	0.312772338252
0.978876531798	0.707756665470	0.032170109784
0.330867027569	0.759703135893	0.080669584458
0.830866060219	0.759703135893	0.080669584458
0.978876496246	0.207756725744	0.032170109784
0.678834318798	0.259703196167	0.080669584458
0.830866024667	0.259703196167	0.080669584458
0.546468836393	0.092939763002	0.040334792252
0.961762374381	0.423528359036	0.122428204866
0.711762858028	0.423528359036	0.122428204866
0.796468352746	0.092939763002	0.040334792252
0.244692329146	0.087353582244	0.164186825228
0.414103389507	0.426176269498	0.000000000000
0.092660623334	0.087353582244	0.164186825228

0.262071683695	0.426176269498	0.000000000000
0.395641066197	0.041283652477	0.112839694288
0.114061212059	0.478122739921	0.048499474675
0.027886549154	0.305773125933	0.132016715444
0.478877463654	0.207756725744	0.032170109784
0.178835286206	0.259703196167	0.080669584458
0.330866992018	0.259703196167	0.080669584458
0.046469803800	0.092939763002	0.040334792252
0.461763341732	0.423528359036	0.122428204866
0.211763825435	0.423528359036	0.122428204866
0.296469320097	0.092939763002	0.040334792252
0.527885581804	0.305773125933	0.132016715444
0.678834354350	0.759703135893	0.080669584458
0.614060244708	0.478122739921	0.048499474675
0.762070716287	0.426176269498	0.000000000000
0.546468871912	0.592939702663	0.040334792252
0.961762409844	0.923528298698	0.122428204866
0.711762893547	0.923528298698	0.122428204866
0.796468388209	0.592939702663	0.040334792252
0.244692364665	0.587353521905	0.164186825228
0.414103425026	0.926176209160	0.000000000000
0.092660658853	0.587353521905	0.164186825228
0.262071719214	0.926176209160	0.000000000000
0.395641101716	0.541283592139	0.112839694288
0.895640098790	0.041283652477	0.112839694288
0.114061247578	0.978122679583	0.048499474675
0.478877499206	0.707756665470	0.032170109784
0.178835321757	0.759703135893	0.080669584458
0.046469839320	0.592939702663	0.040334792252
0.461763377251	0.923528298698	0.122428204866
0.211763860955	0.923528298698	0.122428204866
0.296469355616	0.592939702663	0.040334792252
0.744691361795	0.087353582244	0.164186825228
0.914102422156	0.426176269498	0.000000000000
0.592659655926	0.087353582244	0.164186825228
0.027886584674	0.805773065595	0.132016715444
0.495187903347	0.631404535352	0.428863887417
0.390573579601	0.626348889062	0.430727113453
0.481375709884	0.714640526228	0.392267455810

4. CH_2* and H* on $CoGa_3$

C Co Ga H

1.0

17.550000000000	0.000000000000	0.000000000000
-8.774985327665	15.455371163103	0.000000000000
0.000000000000	0.000000000000	21.997800000000

C Co Ga H

```
  1  32  96   3
Selective dynamics
Direct
  0.373175799003   0.641644431288   0.374309434626
  0.602222909280   0.954447898101   0.153925148651
  0.821302877904   0.892608699359   0.090931261035
  0.538804210476   0.673528328899   0.122428204866
  0.884721541188   0.673528328899   0.122428204866
  0.102223876630   0.954447898101   0.153925148651
  0.038805177827   0.673528328899   0.122428204866
  0.384722508596   0.673528328899   0.122428204866
  0.602222873760   0.454447958440   0.153925148651
  0.821302842352   0.392608759632   0.090931261035
  0.538804174957   0.173528389237   0.122428204866
  0.884721505669   0.173528389237   0.122428204866
  0.102223841111   0.454447958440   0.153925148651
  0.321303809760   0.392608759632   0.090931261035
  0.038805142307   0.173528389237   0.122428204866
  0.384722473076   0.173528389237   0.122428204866
  0.321303845255   0.892608699359   0.090931261035
  0.651761723750   0.554182307913   0.252647685471
  0.929613757603   0.610346013028   0.313489151054
  0.223074354101   0.335097516835   0.278449115296
  0.362677750590   0.334500882821   0.278515552215
  0.151597568720   0.054287941300   0.252268500201
  0.724446887754   0.335814938505   0.278721033903
  0.862038155703   0.336174519124   0.278718761068
  0.651906488194   0.054326176486   0.252361783561
  0.430168824772   0.110993199175   0.313586460651
  0.223646537896   0.836816272744   0.278922382942
  0.361335040626   0.836313491978   0.278263890384
  0.152114124046   0.554885032743   0.252797978421
  0.430741011717   0.607014779788   0.318200257046
  0.723624734205   0.835539657599   0.278476601623
  0.929503513688   0.109911691113   0.313642382524
  0.860981349904   0.835695956771   0.278952919793
  0.355918803634   0.461985459089   0.306615225074
  0.440103154041   0.128364176181   0.212813464590
  0.225291617617   0.201113349937   0.281339126921
  0.610733602934   0.248583757519   0.216850749065
  0.787352839849   0.085353227776   0.300564806391
  0.887680634923   0.247967790553   0.217242859796
  0.306802728123   0.362626348882   0.193178246885
  0.667866121749   0.425716562379   0.251159980467
  0.547700101080   0.086050291203   0.300396601455
  0.008845036128   0.424286749758   0.250987607722
```

0.592659691446	0.587353521905	0.164186825228
0.387502775782	0.246108484311	0.217384909550
0.287731744624	0.086226211628	0.300494507393
0.047618868093	0.086183669101	0.300145289514
0.109665923357	0.248220677607	0.216940018796
0.744691397315	0.587353521905	0.164186825228
0.914102457676	0.926176209160	0.000000000000
0.762070751807	0.926176209160	0.000000000000
0.895640134309	0.541283592139	0.112839694288
0.614060280228	0.978122679583	0.048499474675
0.509035909349	0.422490196225	0.251224712261
0.170432065863	0.428404868651	0.251922781124
0.809493687017	0.368725139185	0.193910221678
0.787676461534	0.586246445608	0.300689100348
0.939315008061	0.129035101063	0.212948066028
0.724687452216	0.700821393890	0.281964627430
0.939454977913	0.629461455703	0.212826328082
0.856368619126	0.963908103738	0.312923928342
0.809460393015	0.868915973772	0.193984864489
0.508023327274	0.925408510558	0.251382442224
0.666399340707	0.925275183282	0.251374677921
0.887641838490	0.748487804796	0.217107174011
0.527885617323	0.805773065595	0.132016715444
0.545974830294	0.583758427269	0.296294292260
0.611083043697	0.749583782912	0.215657614819
0.224288453091	0.702675073285	0.281258941734
0.437832719623	0.628974534062	0.214065497298
0.356811349576	0.964671586009	0.312429820528
0.308929561709	0.869560924910	0.194013849520
0.007891287410	0.925389146438	0.251507557787
0.166635727210	0.926026052674	0.251585841796
0.386261978946	0.748056263003	0.216168972721
0.279717532405	0.587513185121	0.307024932587
0.046996943352	0.586000487320	0.298975752254
0.110099940849	0.749206936896	0.217069582591
0.725642827412	0.201047720034	0.281195453744
0.856611179450	0.464226389264	0.312656717070
0.978876531798	0.707756665470	0.032170109784
0.330867027569	0.759703135893	0.080669584458
0.830866060219	0.759703135893	0.080669584458
0.978876496246	0.207756725744	0.032170109784
0.678834318798	0.259703196167	0.080669584458
0.830866024667	0.259703196167	0.080669584458
0.546468836393	0.092939763002	0.040334792252
0.961762374381	0.423528359036	0.122428204866
0.711762858028	0.423528359036	0.122428204866

0.796468352746	0.092939763002	0.040334792252
0.244692329146	0.087353582244	0.164186825228
0.414103389507	0.426176269498	0.000000000000
0.092660623334	0.087353582244	0.164186825228
0.262071683695	0.426176269498	0.000000000000
0.395641066197	0.041283652477	0.112839694288
0.114061212059	0.478122739921	0.048499474675
0.027886549154	0.305773125933	0.132016715444
0.478877463654	0.207756725744	0.032170109784
0.178835286206	0.259703196167	0.080669584458
0.330866992018	0.259703196167	0.080669584458
0.046469803800	0.092939763002	0.040334792252
0.461763341732	0.423528359036	0.122428204866
0.211763825435	0.423528359036	0.122428204866
0.296469320097	0.092939763002	0.040334792252
0.527885581804	0.305773125933	0.132016715444
0.678834354350	0.759703135893	0.080669584458
0.614060244708	0.478122739921	0.048499474675
0.762070716287	0.426176269498	0.000000000000
0.546468871912	0.592939702663	0.040334792252
0.961762409844	0.923528298698	0.122428204866
0.711762893547	0.923528298698	0.122428204866
0.796468388209	0.592939702663	0.040334792252
0.244692364665	0.587353521905	0.164186825228
0.414103425026	0.926176209160	0.000000000000
0.092660658853	0.587353521905	0.164186825228
0.262071719214	0.926176209160	0.000000000000
0.395641101716	0.541283592139	0.112839694288
0.895640098790	0.041283652477	0.112839694288
0.114061247578	0.978122679583	0.048499474675
0.478877499206	0.707756665470	0.032170109784
0.178835321757	0.759703135893	0.080669584458
0.046469839320	0.592939702663	0.040334792252
0.461763377251	0.923528298698	0.122428204866
0.211763860955	0.923528298698	0.122428204866
0.296469355616	0.592939702663	0.040334792252
0.744691361795	0.087353582244	0.164186825228
0.914102422156	0.426176269498	0.000000000000
0.592659655926	0.087353582244	0.164186825228
0.027886584674	0.805773065595	0.132016715444
0.470082137123	0.588774995469	0.373273047729
0.348489327498	0.614532903599	0.419483445827
0.400239631548	0.712456062072	0.372888440984

5. CH_2* on $CoGa_3$

C Co Ga H

1.0

17.550000000000   0.000000000000   0.000000000000
-8.774985327665 15.455371163103   0.000000000000
0.000000000000   0.000000000000 21.997800000000
C Co Ga H
1 32 96   2
Selective dynamics
Direct
0.364675821757   0.612684767562   0.382992409385
0.602222909280   0.954447898101   0.153925148651
0.821302877904   0.892608699359   0.090931261035
0.538804210476   0.673528328899   0.122428204866
0.884721541188   0.673528328899   0.122428204866
0.102223876630   0.954447898101   0.153925148651
0.038805177827   0.673528328899   0.122428204866
0.384722508596   0.673528328899   0.122428204866
0.602222873760   0.454447958440   0.153925148651
0.821302842352   0.392608759632   0.090931261035
0.538804174957   0.173528389237   0.122428204866
0.884721505669   0.173528389237   0.122428204866
0.102223841111   0.454447958440   0.153925148651
0.321303809760   0.392608759632   0.090931261035
0.038805142307   0.173528389237   0.122428204866
0.384722473076   0.173528389237   0.122428204866
0.321303845255   0.892608699359   0.090931261035
0.651182688700   0.554797096813   0.252734468217
0.929802376886   0.610526987426   0.313481230262
0.223225235549   0.335201919477   0.278393776355
0.362624370534   0.333856650145   0.278751416979
0.151568793706   0.054016779713   0.252239090179
0.724298928118   0.336185564751   0.278756270539
0.861958450476   0.336295028492   0.278658902287
0.652035323810   0.054535365840   0.252348767242
0.430039762743   0.110675021887   0.313516096425
0.224079027973   0.836715978771   0.278623577203
0.361500784873   0.836069783209   0.278145387142
0.152380820869   0.554694737851   0.252937207203
0.424055826327   0.604470604645   0.317706880285
0.724013974469   0.836063476440   0.278651542552
0.929804508986   0.110322502928   0.313646778648
0.861007760142   0.835861403428   0.279103769888
0.357361788203   0.459828405844   0.310180615190
0.440043893857   0.128469958772   0.212786531580
0.225141256261   0.201066898028   0.281032553321
0.610628555824   0.248588843300   0.216911715415
0.787595973943   0.085892703460   0.300600433235
0.887538972210   0.247886253073   0.217306732926

0.308222677481	0.364972452841	0.193764351274
0.667098304031	0.425813809678	0.251322287941
0.547652744068	0.085877392795	0.300366385873
0.008606162399	0.424742470181	0.250698111991
0.592659691446	0.587353521905	0.164186825228
0.387167664950	0.245907036425	0.217159415405
0.287585235316	0.086055276209	0.300398535566
0.047838188014	0.086334479904	0.300117295684
0.109961228373	0.247720236281	0.216939315416
0.744691397315	0.587353521905	0.164186825228
0.914102457676	0.926176209160	0.000000000000
0.762070751807	0.926176209160	0.000000000000
0.895640134309	0.541283592139	0.112839694288
0.614060280228	0.978122679583	0.048499474675
0.508558388601	0.423324693849	0.251008955409
0.168874574936	0.426581187966	0.251260722198
0.809308139574	0.368753154743	0.193930700031
0.787804123261	0.586713063045	0.300010770921
0.939354017812	0.129134550161	0.212919546445
0.723824575800	0.700966522224	0.282683912178
0.939294154009	0.629592097528	0.212774618912
0.856697913913	0.964323464702	0.313101878315
0.809802218158	0.869243488399	0.194059482846
0.508342306165	0.925524366929	0.251387658870
0.666971453465	0.925843748523	0.251467096027
0.887403045598	0.748735132706	0.216950012349
0.527885617323	0.805773065595	0.132016715444
0.543028813288	0.584396980729	0.296119130215
0.612759885607	0.750857757347	0.214822320603
0.224689466136	0.702432258724	0.280200831486
0.440371929632	0.629102469930	0.213920888700
0.356882315146	0.964489935468	0.312837170754
0.309436159202	0.870166213989	0.193993292763
0.007897808998	0.925468573297	0.251568179143
0.166624863668	0.925783050340	0.251447787179
0.386978396650	0.749074078955	0.215547814647
0.278520906104	0.584524677335	0.306217018764
0.047079625864	0.585703846094	0.299677274490
0.109978838149	0.749016405881	0.217146769798
0.725500044490	0.201350398414	0.281231236213
0.856654165094	0.464392503420	0.312642202267
0.978876531798	0.707756665470	0.032170109784
0.330867027569	0.759703135893	0.080669584458
0.830866060219	0.759703135893	0.080669584458
0.978876496246	0.207756725744	0.032170109784
0.678834318798	0.259703196167	0.080669584458

0.830866024667	0.259703196167	0.080669584458
0.546468836393	0.092939763002	0.040334792252
0.961762374381	0.423528359036	0.122428204866
0.711762858028	0.423528359036	0.122428204866
0.796468352746	0.092939763002	0.040334792252
0.244692329146	0.087353582244	0.164186825228
0.414103389507	0.426176269498	0.000000000000
0.092660623334	0.087353582244	0.164186825228
0.262071683695	0.426176269498	0.000000000000
0.395641066197	0.041283652477	0.112839694288
0.114061212059	0.478122739921	0.048499474675
0.027886549154	0.305773125933	0.132016715444
0.478877463654	0.207756725744	0.032170109784
0.178835286206	0.259703196167	0.080669584458
0.330866992018	0.259703196167	0.080669584458
0.046469803800	0.092939763002	0.040334792252
0.461763341732	0.423528359036	0.122428204866
0.211763825435	0.423528359036	0.122428204866
0.296469320097	0.092939763002	0.040334792252
0.527885581804	0.305773125933	0.132016715444
0.678834354350	0.759703135893	0.080669584458
0.614060244708	0.478122739921	0.048499474675
0.762070716287	0.426176269498	0.000000000000
0.546468871912	0.592939702663	0.040334792252
0.961762409844	0.923528298698	0.122428204866
0.711762893547	0.923528298698	0.122428204866
0.796468388209	0.592939702663	0.040334792252
0.244692364665	0.587353521905	0.164186825228
0.414103425026	0.926176209160	0.000000000000
0.092660658853	0.587353521905	0.164186825228
0.262071719214	0.926176209160	0.000000000000
0.395641101716	0.541283592139	0.112839694288
0.114061247578	0.978122679583	0.048499474675
0.027886584674	0.805773065595	0.132016715444
0.478877499206	0.707756665470	0.032170109784
0.178835321757	0.759703135893	0.080669584458
0.046469839320	0.592939702663	0.040334792252
0.461763377251	0.923528298698	0.122428204866
0.211763860955	0.923528298698	0.122428204866
0.296469355616	0.592939702663	0.040334792252
0.744691361795	0.087353582244	0.164186825228
0.914102422156	0.426176269498	0.000000000000
0.592659655926	0.087353582244	0.164186825228
0.895640098790	0.041283652477	0.112839694288
0.318551703948	0.567493080913	0.416642456399
0.394184435580	0.680763312893	0.398299723387

6. CH* and H* on $CoGa_3$

C Co Ga H
1.0
 17.550000000000   0.000000000000   0.000000000000
 -8.774985327665 15.455371163103   0.000000000000
  0.000000000000   0.000000000000 21.997800000000
C Co Ga H
  1 32 96   2
Selective dynamics
Direct
  0.491802418767 0.649488612093 0.376967650575
  0.602222909280 0.954447898101 0.153925148651
  0.821302877904 0.892608699359 0.090931261035
  0.538804210476 0.673528328899 0.122428204866
  0.884721541188 0.673528328899 0.122428204866
  0.102223876630 0.954447898101 0.153925148651
  0.038805177827 0.673528328899 0.122428204866
  0.384722508596 0.673528328899 0.122428204866
  0.602222873760 0.454447958440 0.153925148651
  0.821302842352 0.392608759632 0.090931261035
  0.538804174957 0.173528389237 0.122428204866
  0.884721505669 0.173528389237 0.122428204866
  0.102223841111 0.454447958440 0.153925148651
  0.321303809760 0.392608759632 0.090931261035
  0.038805142307 0.173528389237 0.122428204866
  0.384722473076 0.173528389237 0.122428204866
  0.321303845255 0.892608699359 0.090931261035
  0.652315951361 0.555671483921 0.252143221657
  0.930882644118 0.610852822323 0.313313216411
  0.222536554521 0.334567845059 0.278739752556
  0.362103338916 0.335590799202 0.278896209723
  0.151810940616 0.053680780631 0.252207868171
  0.723763162976 0.336645520341 0.278861268534
  0.861752109850 0.336409610562 0.278545252888
  0.652089789602 0.054605953527 0.252368662878
  0.429962044126 0.110211370082 0.313707527995
  0.224415957276 0.835650518552 0.278945531123
  0.361561505533 0.836301110224 0.278516358609
  0.153991673780 0.554763974651 0.252927405197
  0.433531850049 0.611970675865 0.314178430120
  0.724387527598 0.836667778065 0.278567253938
  0.930166854508 0.110646149779 0.313702432679
  0.860754108806 0.835848271688 0.278800185649
  0.356532105898 0.464205582663 0.306614295654
  0.439424743397 0.129169994936 0.212925891826
  0.225818018982 0.200941958055 0.280963069831

0.610208711364	0.248202730340	0.217125538128
0.787743010599	0.085881957235	0.300474110266
0.887575317438	0.248481678320	0.216969281378
0.306796190576	0.364097115144	0.193511725730
0.665178949969	0.425138941643	0.251249527586
0.547814984248	0.085946754334	0.300436299761
0.008873424939	0.425628811973	0.251128436387
0.592659691446	0.587353521905	0.164186825228
0.389031633523	0.249314639356	0.216984983479
0.287894395922	0.085971835810	0.300042615019
0.048291153528	0.086628751861	0.300126879681
0.109817114392	0.247398656959	0.216882579427
0.744691397315	0.587353521905	0.164186825228
0.914102457676	0.926176209160	0.000000000000
0.762070751807	0.926176209160	0.000000000000
0.895640134309	0.541283592139	0.112839694288
0.614060280228	0.978122679583	0.048499474675
0.507443216877	0.431046272682	0.252322534234
0.166621180964	0.424879946312	0.251911802991
0.808752279443	0.368502732756	0.193899798776
0.788844622081	0.587590514232	0.299016219697
0.938775383251	0.128936654722	0.212888637247
0.723075206326	0.701148427644	0.280197373775
0.939634315024	0.629871759912	0.212726519131
0.857298671429	0.964692949684	0.313038137681
0.809345246838	0.869158675980	0.193915351033
0.508506418746	0.925504499537	0.251288480778
0.666538903843	0.925455829147	0.251352266780
0.887867521458	0.749227039949	0.216698767769
0.527885617323	0.805773065595	0.132016715444
0.553963494607	0.577922453740	0.324646991773
0.609003077061	0.747198242199	0.218254254055
0.226048583427	0.700897627025	0.281657222980
0.439393530347	0.627252878981	0.211896370071
0.356880626065	0.964343550463	0.313021985101
0.308969673384	0.868290352888	0.193830200496
0.007984079863	0.925399171982	0.251747453554
0.166669403470	0.924922181047	0.251331503348
0.389205370946	0.748753295397	0.217086308747
0.292371930442	0.586974416065	0.298624268517
0.048486164493	0.585629214829	0.300606749941
0.110270362957	0.748354093387	0.217268071103
0.725564127865	0.201632580389	0.281156270434
0.857232891278	0.464768410931	0.311782273403
0.978876531798	0.707756665470	0.032170109784
0.330867027569	0.759703135893	0.080669584458

0.830866060219	0.759703135893	0.080669584458
0.978876496246	0.207756725744	0.032170109784
0.678834318798	0.259703196167	0.080669584458
0.830866024667	0.259703196167	0.080669584458
0.546468836393	0.092939763002	0.040334792252
0.961762374381	0.423528359036	0.122428204866
0.711762858028	0.423528359036	0.122428204866
0.796468352746	0.092939763002	0.040334792252
0.244692329146	0.087353582244	0.164186825228
0.414103389507	0.426176269498	0.000000000000
0.092660623334	0.087353582244	0.164186825228
0.262071683695	0.426176269498	0.000000000000
0.395641066197	0.041283652477	0.112839694288
0.114061212059	0.478122739921	0.048499474675
0.027886549154	0.305773125933	0.132016715444
0.478877463654	0.207756725744	0.032170109784
0.178835286206	0.259703196167	0.080669584458
0.330866992018	0.259703196167	0.080669584458
0.046469803800	0.092939763002	0.040334792252
0.461763341732	0.423528359036	0.122428204866
0.211763825435	0.423528359036	0.122428204866
0.296469320097	0.092939763002	0.040334792252
0.527885581804	0.305773125933	0.132016715444
0.678834354350	0.759703135893	0.080669584458
0.614060244708	0.478122739921	0.048499474675
0.762070716287	0.426176269498	0.000000000000
0.546468871912	0.592939702663	0.040334792252
0.961762409844	0.923528298698	0.122428204866
0.711762893547	0.923528298698	0.122428204866
0.796468388209	0.592939702663	0.040334792252
0.244692364665	0.587353521905	0.164186825228
0.414103425026	0.926176209160	0.000000000000
0.092660658853	0.587353521905	0.164186825228
0.262071719214	0.926176209160	0.000000000000
0.395641101716	0.541283592139	0.112839694288
0.114061247578	0.978122679583	0.048499474675
0.027886584674	0.805773065595	0.132016715444
0.478877499206	0.707756665470	0.032170109784
0.178835321757	0.759703135893	0.080669584458
0.046469839320	0.592939702663	0.040334792252
0.461763377251	0.923528298698	0.122428204866
0.211763860955	0.923528298698	0.122428204866
0.296469355616	0.592939702663	0.040334792252
0.744691361795	0.087353582244	0.164186825228
0.914102422156	0.426176269498	0.000000000000
0.592659655926	0.087353582244	0.164186825228

0.895640098790   0.041283652477   0.112839694288
0.576853526946   0.561639883469   0.392840558758
0.527882585983   0.670247130818   0.420229299097

7. CH* on $CoGa_3$

C Co Ga H
1.0
17.550000000000   0.000000000000   0.000000000000
-8.774985327665 15.455371163103   0.000000000000
0.000000000000   0.000000000000 21.997800000000
C Co Ga H
1 32 96   1
Selective dynamics
Direct
0.457386934295   0.660816971069   0.382455163991
0.602222909280   0.954447898101   0.153925148651
0.821302877904   0.892608699359   0.090931261035
0.538804210476   0.673528328899   0.122428204866
0.884721541188   0.673528328899   0.122428204866
0.102223876630   0.954447898101   0.153925148651
0.038805177827   0.673528328899   0.122428204866
0.384722508596   0.673528328899   0.122428204866
0.602222873760   0.454447958440   0.153925148651
0.821302842352   0.392608759632   0.090931261035
0.538804174957   0.173528389237   0.122428204866
0.884721505669   0.173528389237   0.122428204866
0.102223841111   0.454447958440   0.153925148651
0.321303809760   0.392608759632   0.090931261035
0.038805142307   0.173528389237   0.122428204866
0.384722473076   0.173528389237   0.122428204866
0.321303845255   0.892608699359   0.090931261035
0.650724220597   0.555040264337   0.252972754813
0.930216200339   0.610652408319   0.313429243785
0.222732396409   0.333898203708   0.278770530892
0.360780550593   0.333944825104   0.278735284978
0.151679940208   0.053489344198   0.252180777441
0.724134077455   0.336501522666   0.278662350671
0.862176921610   0.336573366967   0.278689017394
0.652277545494   0.054810651689   0.252424065232
0.430250293336   0.110802572804   0.313708262882
0.224026106756   0.836014355577   0.279151253091
0.361271151259   0.837081209884   0.278219834314
0.154068041787   0.554974133815   0.252961228929
0.430199061160   0.610350480976   0.317265236559
0.725514257194   0.837220687659   0.278241623989
0.930430029822   0.110849896615   0.313698881545
0.861775939463   0.836072743697   0.279157265295

0.308069496612	0.366545463468	0.193947849629
0.355420038651	0.461534458051	0.311163784226
0.439679700376	0.129339980011	0.212904227859
0.224891786721	0.200025852312	0.280900306346
0.547962929799	0.086307329847	0.300411357284
0.788140647830	0.086361654779	0.300538537479
0.009099457652	0.425896248849	0.251215711833
0.887813012324	0.248590494561	0.217036320087
0.610483831472	0.248429252616	0.217070540412
0.165897769854	0.423798853142	0.252005618814
0.762070751807	0.926176209160	0.000000000000
0.287979538411	0.086116933809	0.300225912745
0.047933756892	0.086143643371	0.300119609594
0.110412125180	0.247773462071	0.216241362984
0.744691397315	0.587353521905	0.164186825228
0.914102457676	0.926176209160	0.000000000000
0.592659691446	0.587353521905	0.164186825228
0.895640134309	0.541283592139	0.112839694288
0.614060280228	0.978122679583	0.048499474675
0.666541917289	0.425862504506	0.251269030921
0.387219028977	0.247966751876	0.216233268736
0.507609142525	0.423929617297	0.252021768337
0.787654874195	0.586321018202	0.300182346385
0.856970767438	0.464582088757	0.312404299971
0.939613943608	0.629650029421	0.212786266786
0.857618757957	0.964846375988	0.313362582600
0.811368896097	0.870440733782	0.194074837552
0.508685876148	0.925832614876	0.251690765358
0.666953111103	0.925939309138	0.251641213109
0.887877199010	0.748822808048	0.216908226095
0.527885617323	0.805773065595	0.132016715444
0.544406795748	0.585062580431	0.297622093245
0.614282503179	0.751863585366	0.214351462465
0.226170073775	0.701466615882	0.281556317066
0.441280318064	0.632743004867	0.213533136936
0.356900791058	0.964831414981	0.313305051039
0.308797353182	0.870297301050	0.194022542721
0.008302711978	0.925213842009	0.251583595624
0.166659055624	0.925171243686	0.251539444433
0.387361513754	0.751673213904	0.214464897893
0.290537327278	0.584961711807	0.297592523825
0.048141505645	0.586025617868	0.300147557752
0.110459683122	0.748546138759	0.217107161758
0.725816978323	0.201751370644	0.280913285346
0.939319374794	0.129336370083	0.212888032103
0.809334157971	0.368831840994	0.193950267868

0.978876531798	0.707756665470	0.032170109784
0.046469839320	0.592939702663	0.040334792252
0.830866060219	0.759703135893	0.080669584458
0.978876496246	0.207756725744	0.032170109784
0.678834318798	0.259703196167	0.080669584458
0.830866024667	0.259703196167	0.080669584458
0.546468836393	0.092939763002	0.040334792252
0.961762374381	0.423528359036	0.122428204866
0.711762858028	0.423528359036	0.122428204866
0.796468352746	0.092939763002	0.040334792252
0.244692329146	0.087353582244	0.164186825228
0.414103389507	0.426176269498	0.000000000000
0.092660623334	0.087353582244	0.164186825228
0.262071683695	0.426176269498	0.000000000000
0.395641066197	0.041283652477	0.112839694288
0.114061212059	0.478122739921	0.048499474675
0.027886549154	0.305773125933	0.132016715444
0.478877463654	0.207756725744	0.032170109784
0.178835286206	0.259703196167	0.080669584458
0.330866992018	0.259703196167	0.080669584458
0.046469803800	0.092939763002	0.040334792252
0.461763341732	0.423528359036	0.122428204866
0.211763825435	0.423528359036	0.122428204866
0.296469320097	0.092939763002	0.040334792252
0.527885581804	0.305773125933	0.132016715444
0.678834354350	0.759703135893	0.080669584458
0.614060244708	0.478122739921	0.048499474675
0.762070716287	0.426176269498	0.000000000000
0.546468871912	0.592939702663	0.040334792252
0.961762409844	0.923528298698	0.122428204866
0.711762893547	0.923528298698	0.122428204866
0.796468388209	0.592939702663	0.040334792252
0.244692364665	0.587353521905	0.164186825228
0.414103425026	0.926176209160	0.000000000000
0.092660658853	0.587353521905	0.164186825228
0.262071719214	0.926176209160	0.000000000000
0.395641101716	0.541283592139	0.112839694288
0.114061247578	0.978122679583	0.048499474675
0.027886584674	0.805773065595	0.132016715444
0.478877499206	0.707756665470	0.032170109784
0.178835321757	0.759703135893	0.080669584458
0.330867027569	0.759703135893	0.080669584458
0.725154966955	0.701619938424	0.281585957803
0.461763377251	0.923528298698	0.122428204866
0.211763860955	0.923528298698	0.122428204866
0.296469355616	0.592939702663	0.040334792252

```
  0.744691361795   0.087353582244   0.164186825228
  0.914102422156   0.426176269498   0.000000000000
  0.592659655926   0.087353582244   0.164186825228
  0.895640098790   0.041283652477   0.112839694288
  0.473180034161   0.688257038980   0.428735622400
```

8. C* and H* on $CoGa_3$

```
C Co Ga H
1.0
 17.550000000000   0.000000000000   0.000000000000
 -8.774985327665  15.455371163103   0.000000000000
  0.000000000000   0.000000000000  21.997800000000
C Co Ga H
  1  32  96   1
Selective dynamics
Direct
  0.434876444289   0.661465466556   0.349657587950
  0.602222909280   0.954447898101   0.153925148651
  0.821302877904   0.892608699359   0.090931261035
  0.538804210476   0.673528328899   0.122428204866
  0.884721541188   0.673528328899   0.122428204866
  0.102223876630   0.954447898101   0.153925148651
  0.038805177827   0.673528328899   0.122428204866
  0.384722508596   0.673528328899   0.122428204866
  0.602222873760   0.454447958440   0.153925148651
  0.821302842352   0.392608759632   0.090931261035
  0.538804174957   0.173528389237   0.122428204866
  0.884721505669   0.173528389237   0.122428204866
  0.102223841111   0.454447958440   0.153925148651
  0.321303809760   0.392608759632   0.090931261035
  0.038805142307   0.173528389237   0.122428204866
  0.384722473076   0.173528389237   0.122428204866
  0.321303845255   0.892608699359   0.090931261035
  0.651979294134   0.554073438521   0.251656493871
  0.930327261776   0.610086766391   0.313426607556
  0.222783513953   0.332474047828   0.278767902772
  0.358158402952   0.333771062076   0.279035975002
  0.151734886656   0.052982760605   0.252078725329
  0.722936141100   0.335792029924   0.278976677958
  0.860238985175   0.335487918754   0.278467124926
  0.651902160087   0.053774912448   0.252374683146
  0.429756946742   0.109420855769   0.313881643833
  0.223758958223   0.834562125983   0.279356780302
  0.360461228853   0.835843318641   0.278079677253
  0.152303244873   0.553107838448   0.252732298396
  0.418807029859   0.581256785913   0.308629492068
  0.725217343062   0.835842624879   0.278365640370
```

0.930099319615	0.109603509687	0.313761343281
0.861951537546	0.835833964476	0.279015838838
0.315288137035	0.381516384418	0.196721696429
0.344674193194	0.438411445279	0.338783478093
0.438352456156	0.129810577347	0.213251393143
0.224742747563	0.198673511907	0.278459385011
0.547415723203	0.085001994389	0.300439184037
0.787653634264	0.084819045651	0.300235795266
0.006712790246	0.425829790911	0.251298240748
0.887235995355	0.248333210401	0.216856955907
0.609356670416	0.247179981261	0.217458997658
0.158878068301	0.416769554208	0.249755058565
0.762070751807	0.926176209160	0.000000000000
0.287721263115	0.084573278932	0.300055742041
0.046972929821	0.083949842863	0.300628844080
0.110589421574	0.246755177180	0.215422527628
0.744691397315	0.587353521905	0.164186825228
0.914102457676	0.926176209160	0.000000000000
0.592659691446	0.587353521905	0.164186825228
0.895640134309	0.541283592139	0.112839694288
0.614060280228	0.978122679583	0.048499474675
0.663238374287	0.423482862307	0.250794861451
0.390063193294	0.252841127616	0.214935421176
0.506029665890	0.432939653625	0.256805332167
0.787569097823	0.584817743918	0.298846080682
0.857434089051	0.464072831396	0.312295241554
0.939603998756	0.629469656546	0.212831181143
0.856706177033	0.963578999366	0.314179184341
0.811053726801	0.870194953018	0.194100324719
0.508202367715	0.924971352208	0.251885590331
0.666219749136	0.924108538456	0.251233500536
0.887960439787	0.748615430853	0.216853520489
0.527885617323	0.805773065595	0.132016715444
0.557654023902	0.587124712116	0.320714994646
0.614642231900	0.747903535873	0.215361924737
0.227406254248	0.699398745180	0.281983237551
0.443508958071	0.635547265527	0.211865074873
0.356921850401	0.963440033964	0.314541262505
0.308394414883	0.870168012044	0.194064256212
0.008260982423	0.924371805387	0.251042772766
0.166266338781	0.923930713614	0.251451344690
0.386200301624	0.752358555315	0.212900330208
0.281150652962	0.569830784756	0.297270912473
0.048788875649	0.586246266519	0.300894971707
0.110431505078	0.747637140982	0.217169396512
0.724922157658	0.200521522381	0.280995807027

0.939172241509	0.129262879588	0.213133687458
0.807879024188	0.368128083533	0.193882485657
0.978876531798	0.707756665470	0.032170109784
0.046469839320	0.592939702663	0.040334792252
0.830866060219	0.759703135893	0.080669584458
0.978876496246	0.207756725744	0.032170109784
0.678834318798	0.259703196167	0.080669584458
0.830866024667	0.259703196167	0.080669584458
0.546468836393	0.092939763002	0.040334792252
0.961762374381	0.423528359036	0.122428204866
0.711762858028	0.423528359036	0.122428204866
0.796468352746	0.092939763002	0.040334792252
0.244692329146	0.087353582244	0.164186825228
0.414103389507	0.426176269498	0.000000000000
0.092660623334	0.087353582244	0.164186825228
0.262071683695	0.426176269498	0.000000000000
0.395641066197	0.041283652477	0.112839694288
0.114061212059	0.478122739921	0.048499474675
0.027886549154	0.305773125933	0.132016715444
0.478877463654	0.207756725744	0.032170109784
0.178835286206	0.259703196167	0.080669584458
0.330866992018	0.259703196167	0.080669584458
0.046469803800	0.092939763002	0.040334792252
0.461763341732	0.423528359036	0.122428204866
0.211763825435	0.423528359036	0.122428204866
0.296469320097	0.092939763002	0.040334792252
0.527885581804	0.305773125933	0.132016715444
0.678834354350	0.759703135893	0.080669584458
0.614060244708	0.478122739921	0.048499474675
0.762070716287	0.426176269498	0.000000000000
0.546468871912	0.592939702663	0.040334792252
0.961762409844	0.923528298698	0.122428204866
0.711762893547	0.923528298698	0.122428204866
0.796468388209	0.592939702663	0.040334792252
0.244692364665	0.587353521905	0.164186825228
0.414103425026	0.926176209160	0.000000000000
0.092660658853	0.587353521905	0.164186825228
0.262071719214	0.926176209160	0.000000000000
0.395641101716	0.541283592139	0.112839694288
0.114061247578	0.978122679583	0.048499474675
0.027886584674	0.805773065595	0.132016715444
0.478877499206	0.707756665470	0.032170109784
0.178835321757	0.759703135893	0.080669584458
0.330867027569	0.759703135893	0.080669584458
0.725848763439	0.700332588732	0.281143702508
0.461763377251	0.923528298698	0.122428204866

0.211763860955	0.923528298698	0.122428204866
0.296469355616	0.592939702663	0.040334792252
0.744691361795	0.087353582244	0.164186825228
0.914102422156	0.426176269498	0.000000000000
0.592659655926	0.087353582244	0.164186825228
0.895640098790	0.041283652477	0.112839694288
0.605645861783	0.631040733693	0.383997056342
